# MRI‐Based Grading Systems for Assessing Lumbar Disc Degeneration: A Scoping Review

**DOI:** 10.1002/jsp2.70113

**Published:** 2025-09-15

**Authors:** Dean Esposito, Benjamin Brown, Mark Jonathan Hancock, Samuel Stuart Graham King, Isaac Gerard Tom Searant, Hazel Jenkins

**Affiliations:** ^1^ Faculty of Medicine, Health and Human Sciences, Macquarie University Sydney Australia

**Keywords:** degenerative disc disease, intervertebral disc, low back pain, lumbar, magnetic resonance imaging, MRI

## Abstract

**Background Context:**

An array of different MRI (magnetic resonance imaging) based grading systems is used to measure disc degeneration (DD) in the lumbar spine. It is currently unclear which grading systems are most commonly used to assess lumbar DD and how these grading systems are applied and reported.

**Purpose:**

The aim of this scoping review was to describe different MRI‐based grading systems for DD in the lumbar spine and report which grading systems have been assessed for measurement properties such as reliability, validity, and sensitivity to change.

**Study Design/Setting:**

Scoping review.

**Methods:**

A search was conducted in EMBASE, Medline, and CINAHL for studies related to MRI‐based grading systems for DD in the lumbar spine, conducted in living humans. Data were extracted from each study, including the description of the grading system, which levels of the lumbar spine were graded, who graded the degeneration, how the degeneration was scored for analysis, and whether measurement properties such as reliability, validity, and sensitivity to change were assessed.

**Results:**

The search identified 569 studies that graded DD. Ninety‐three different grading systems were identified, including 63 subjective systems, 25 quantitative systems, and 5 that were unspecified. The Pfirrmann method was used in over 50% of all reports. A range of grading components was used to measure DD, with disc signal intensity (DSI), disc height (DH), and the assessment of the distinctiveness between the annulus and nucleus being most common. Sensitivity to change was rarely assessed.

**Conclusion:**

A large number of DD grading systems were identified in this review, many of which were infrequently used. Variability in methods of assessing DD on MRI and how the MRI data is synthesized may influence reported associations between DD and low back pain (LBP).

## Graphical abstract

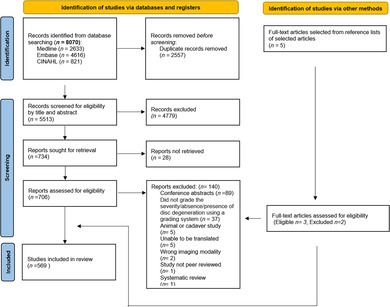

## 1 Introduction

Low back pain (LBP) is a leading cause of global disability [1] with an average lifetime prevalence of between 38% and 80% [2]. Despite this significant burden, limited progress has been made with regard to effective management of LBP [3, 4]. This may be partly due to the inherent difficulty in identifying specific pain‐generating structure/s that could serve as a target for treatment [5]. Morphological changes in the lumbar spine are commonly identified on magnetic resonance imaging (MRI) in patients with LBP [5]. However, these same morphological changes are often observed in asymptomatic populations [6, 7]. Therefore, the clinical importance of morphological changes observed on MRI in patients with LBP remains unclear.

Disc degeneration (DD) is an example of a morphological change that can be identified on MRI that may be associated with LBP. DD is an umbrella term used to represent a range of intervertebral disc changes, which most commonly include narrowing of the intervertebral disc space and alterations in disc signal intensity (DSI) [8, 9]. The clinical relevance of DD for LBP patients is currently uncertain [8]. This may be, in part, due to how changes to the intervertebral disc are measured on MRI [8].

Many different grading systems are used to measure DD in the lumbar spine. These are commonly ordinal‐based scales that employ a subjective assessment of different MRI findings to determine the degree of DD. One example is the Pfirrmann method, where DD is subjectively categorized on a five‐point scale from I (no degeneration) through to V (severe degeneration) [10, 11]. Despite their widespread use and ease of application, subjective grading systems have fundamental limitations; namely, relatively poor inter‐rater reliability and sensitivity to change (the ability for the grading system to identify changes in DD over time) [12, 13, 14]. These grading systems are often used for research purposes rather than within clinical settings.

Quantitative grading systems, on the other hand, measure changes to the intervertebral disc more objectively. Most quantitative grading systems measure DSI and/or disc height (DH) to assess DD [9, 15, 16, 17]. Although these methods provide a reliable measure of DSI and DH, it is unclear whether these measurements reflect the true severity of DD. For example, measurements of DSI and DH can be impacted by diurnal variation, vertebral level, patient age, and height, which may limit their usefulness as measures of between‐person severity [18]. Consequently, the variability of factors unrelated to DD on DSI and DH may influence the grading system's ability to measure the true underlying degenerative process. Many different grading systems exist that use either subjective or quantitative measurements of DD, with many different variations and modifications. It is currently unclear which grading systems are used to assess lumbar DD and how the grading system is summarized, reported, and coded for analysis. Thus, a comprehensive charting of DD grading systems and the methods of synthesis used is required.

The aim of this review is to describe different MRI‐based grading systems for DD in the lumbar spine. This manuscript will focus on how each grading system was summarized for analysis, if measurement properties have been assessed for each grading system, and whether associations have been made between DD and clinical variables such as current and future LBP, and sensitivity to change.

## 2 Materials and Methods

### 2.1 Search Strategy

This scoping review was conducted in accordance with recommendations outlined by the Joanna Briggs Institute (JBI) [19] and reported in accordance with the PRISMA‐ScR (Preferred Reporting Items for Systematic Reviews and Meta‐Analyses) guidelines for systematic reviews [20]. The protocol for this scoping review has been published on the Open Science Framework [21].

An electronic database search was conducted in EMBASE, Medline, and CINAHL from inception to April 5, 2023, for studies relating to MRI‐based grading systems for DD in the lumbar spine. The search strategy was developed in conjunction with a faculty librarian at Macquarie University and adapted for each database (Supporting Information S1). Backward citation tracking was used to identify studies that described a grading system that had been identified in the primary search.

### 2.2 Inclusion and Exclusion Criteria

To be included, studies needed to have used a grading system to assess lumbar spine DD on MRI in living humans. For the purposes of this scoping review, a grading system was defined as any subjective or quantitative system that described the presence or absence of DD or the degree/extent of DD. A subjective grading system was defined as any system that reported on visible intervertebral disc changes that could indicate DD. A quantitative grading system was defined as any system that objectively measured MRI‐based components/features of the intervertebral disc on a continuous scale. A number of specialized quantitative MRI techniques and sequences were categorized together and defined as grading systems that measured the water content and tissue composition within the disc using specific sequences such as T2 mapping. Studies were only included if the authors explicitly stated they were using a grading system to measure DD. This decision was made due to inconsistency/uncertainty in the literature regarding whether certain discal changes (e.g., disc herniation) directly reflected the presence or extent of DD. Studies were excluded if they were unable to be retrieved or translated. We also excluded reviews and studies that were not peer‐reviewed. Conference abstracts were excluded as they did not typically provide a sufficient description of the grading system. The original development/descriptive papers of a particular grading system were not included in the review.

One author (D.E.) screened titles in EndNote [22] and removed duplicates and any overtly ineligible citations. The title, abstract, and full text screening were performed by two authors (D.E., H.Z., M.H., B.B., I.S., S.K.) independently. Abstracts were screened in endnote [22] and full studies were screened in Covidence [23]. Any disagreements at the title, abstract, and full text screening were discussed between authors. A third author was consulted if a consensus regarding an article's eligibility could not be achieved.

### 2.3 Data Extraction

The data extraction tool was adapted from JBI recommendations [19]. One author (D.E.) completed the extraction in Covidence [23], with 10% of the extraction conducted by a second independent author for consistency. We extracted the following data from each study including the year of publication; the country in which the study was conducted study setting; and population characteristics. For each grading system we extracted: (1) the name and description of the grading system; (2) how the MRI was performed (supine or weight‐bearing); (3) which levels of the lumbar spine were graded; (4) who graded the degeneration; (5) how the disc degeneration was summarized (worst level, each disc level collected and analyzed, sum of all levels, average of all levels); and (6) how the grading system was reported (continuous, ordinal, collected as ordinal but analyzed as dichotomous, collected and analyzed as dichotomous). Finally, we extracted details regarding whether assessment of measurement properties such as reliability (intra‐rater, inter‐rater), validity (comparison with another grading system, measured association between DD and other variables, including current and future LBP) and sensitivity to change (reporting a change score of the grading system over time) were performed (yes/no).

### 2.4 Data Synthesis

The extracted results were exported from Covidence [23] to Excel [24] for data cleaning and synthesis. Descriptive statistics (frequency counts and proportions) were calculated for the year of publication and key sample characteristics including age, location, sample population, and setting. The total number of annual publications was calculated for 1986 to 2022, to ensure a full year of data in the most recent year. The year of publication and key sample characteristics were plotted using a cumulative frequency curve and histogram, respectively. The extracted studies were categorized as either a subjective or a quantitative grading system. Descriptive statistics (proportions) were calculated for how the grading systems were used/reported (method of synthesis) in different studies, including the proportion of studies that: used different graders of DD (radiologists, surgeons or not specified); assessed different levels of the lumbar spine (the entire spine, singular levels or other); and reported/summarized the grading system differently (each level individually, worst level, sum of all levels, average across all levels or not specified). The results were tabulated into the categories as listed above, and a summary was created for each grading system.

## 3 Results

### 3.1 Included Studies

**FIGURE 1:**
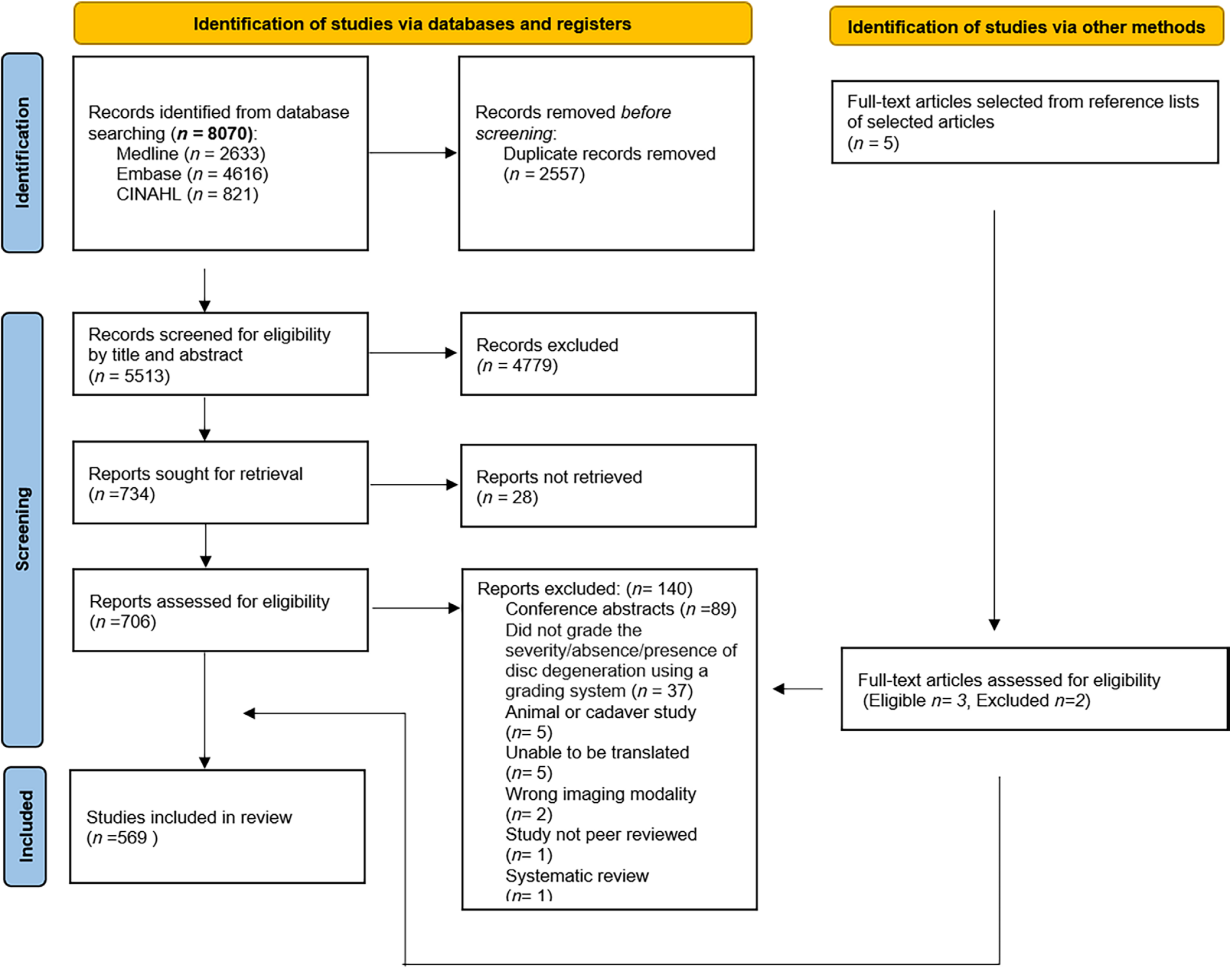
Preferred Reporting Items for Systematic Reviews and Meta‐Analyses flow chart.

A total of 8070 studies were identified from the literature search, with 569 studies included after full‐text screening (Figure 1). The main reasons for full‐text exclusion were that a study was only available in conference abstract form (*n* = 89), or that the study did not grade the severity/presence or absence of disc degeneration using a grading system (*n* = 37). Three studies were identified from backward citation tracking. See Supporting Information S2 for a complete list of all 569 included studies.

**FIGURE 2:**
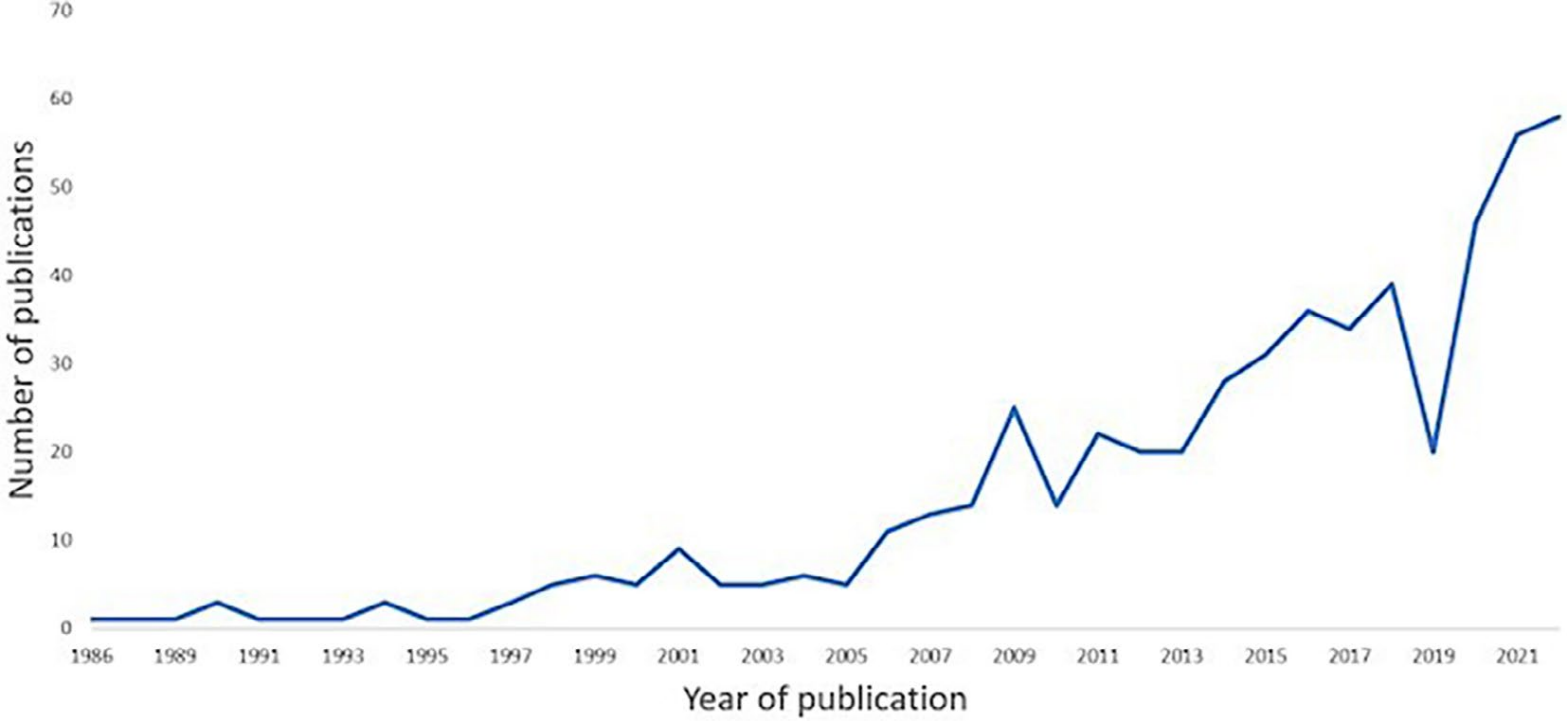
Annual publication counts of studies that used a grading system to assess lumbar spine disc degeneration on MRI in living humans between 1986 and 2022.

**FIGURE 3:**
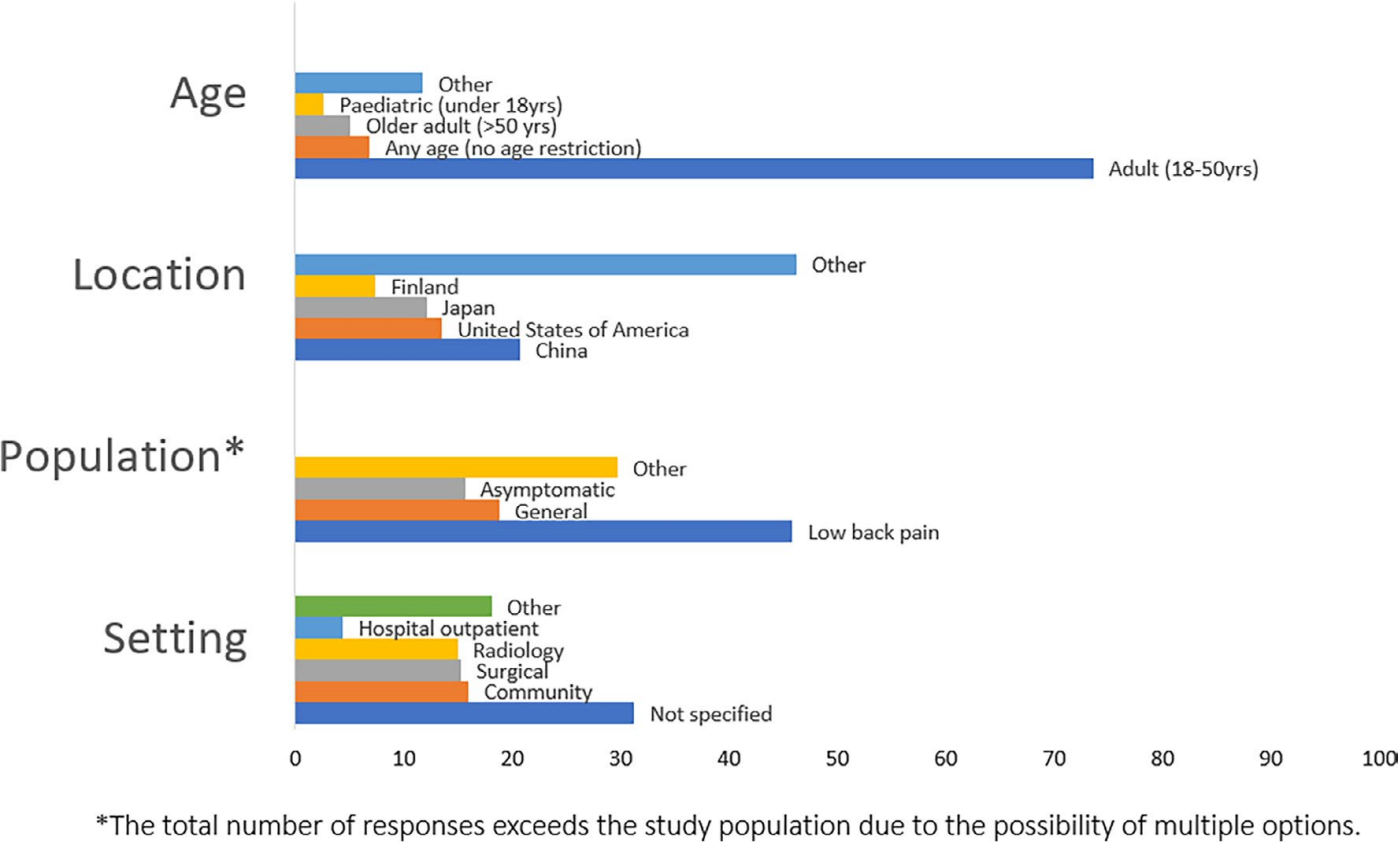
Age, location, sample population, and setting of included studies that used a grading system to assess lumbar spine disc degeneration based on MRI in living humans. *The total number of responses exceeds the study population due to the possibility of multiple options.

The majority of studies were published after 2010 (443/569, 77.9%) (Figure 2). Studies commonly included adults (419/569, 73.6%) from LBP populations (261/569, 46%) (Figure 3), while the study setting was not clearly reported in 31.3% (178/569) of studies. The studies took place in a variety of different countries, including Finland, Japan, and the United States of America; however, the most commonly reported study location was China (118/569, 20.7%).

**FIGURE 4:**
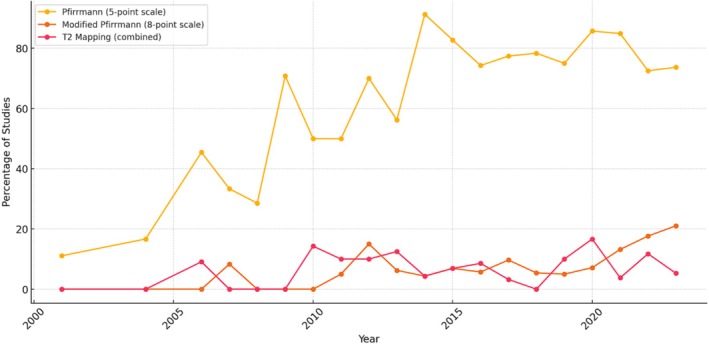
Percentage of studies using selected grading systems over time.

Over time, the Pfirrmann grading system was consistently used, particularly between the early 2000s and mid‐2010s, where it was used in over 50% of studies. In contrast, T2 mapping showed minimal use in early years but increased steadily over time, particularly from 2010 to onwards. This trend reflects a gradual shift toward incorporating more objective, quantitative imaging methods in DD research. See Figure 4 for more details.

### 3.2 Description of Grading Systems

In total, there were 668 reports of grading system use across the 569 studies, as multiple grading systems could be reported within a single study. Ninety‐three different grading systems were identified. Of these, 63/93 (67.7%) were classified as subjective grading systems and 25/93 (26.9%) were quantitative. The remaining 5/93 (5.4%) grading systems were categorized as ‘unspecified’ as the systems lacked a clear classification or description.

Subjective grading systems were used more frequently than quantitative systems (556/668, 83.2% vs. 112/668, 16.8%). The most widely used subjective grading system was the Pfirrmann method [10] (370/668, 55.4%), followed by the Modified Pfirrmann method [11] (42/668, 6.3%) and the Schneiderman classification [25] (30/668, 4.5%). Many of the identified grading systems (60/93, 64.5%) were only reported in single studies. A number of specialized quantitative MRI techniques and sequencing approaches were categorized together and made up 10.8% (72/668) of the reports of grading system use. The most commonly used quantitative MRI techniques and sequences included T2 mapping (29/72, 40.3%), ADC (Apparent Diffusion Coefficient) (13/72, 18.1%), and T1 relaxation (8/72, 11.1%). See Supporting Information S3 and S4 for descriptions of all 93 grading systems.

The components within each grading system that were used to assess for DD varied considerably (Table 1). The most common components used across all grading systems to measure DD were DSI and DH; however, DH was rarely used as a stand‐alone component (seven grading systems used in 1.0% (7/668) of reports of all grading systems). In the reports of subjective grading systems (*n* = 556), combinations of DSI, DH, structural changes to the disc, and the distinction between the boundary between the annulus fibrosis and nucleus pulposus (e.g., Pfirrmann, Modified Pfirrmann) were most commonly used. These features were used in nine grading systems, accounting for 77.5% (431/556) of such reports. Another 14 grading systems, used within 5.9% (33/556) of reports, used subjective assessment of additional grading components such as endplate changes, Modic changes, and high intensity zones (HIZ) as part of the assessment of DD.

**TABLE 1:** The proportion of grading systems reported to be used to assess disc degeneration with different methods of synthesis, stratified by the grading system components used to assess for disc degeneration.

		DD grading performed by^b^		L‐spine levels reported				Method of synthesis I^b^					Method of synthesis II			
Grading system components	Proportion of reported use of grading systems % (n/N)	Radiologist % (n/N)	Surgeon % (n/N)	Not specified % (n/N)	Lumbar spine reported (T12‐S1)* % (n/N)	Single level reported % (n/N)	Other^a^ % (n/N)	Each level individually % (n/N)	Worst level % (n/N)	Sum of all levels % (n/N)	Average across all levels % (n/N)	Not specified % (n/N)	Continuous % (n/N)	Ordinal % (n/N)	Collected as ordinal but analyzed as dichotomous % (n/N)	Collected and analyzed as dichotomous % (n/N)
**Subjective grading systems (all)**	**83.2** **(556/668)**	**42.4** **(236/556)**	**24.1** **(134/556)**	**31.8** **(177/556)**	**53.2** **(296/556)**	**12.6** **(70/556)**	**34.4** **(191/556)**	**58.1** **(323/556)**	**6.1** **(34/556)**	**14.4** **(80/556)**	**3.8** **(21/556)**	**21.8** **(121/556)**	**9.4** **(52/556)**	**58.5** **(325/556)**	**27.5** **(153/556)**	**4.7** **(26/556)**
**DSI**	3.8 (21/556)	57.1 (12/21)	19.0 (4/21)	38.1 (8/21)	33.3 (7/21)	19.0 (4/21)	47.7 (10/21)	71.4 (15/21)	4.8 (1/21)	0.0 (0/21)	0.0 (0/21)	23.8 (5/21)	0.0 (0/21)	42.9 (9/21)	14.3 (3/21)	42.9 (9/21)
**DH**	0.7 (4/556)	75.0 (3/4)	75.0 (3/4)	0.0 (0/4)	75.0 (3/4)	25.0 (1/4)	0.0 (0/4)	50.0 (2/4)	25.0 (1/4)	0.0 (0/4)	25.0 (1/4)	0.0 (0/4)	0.0 (0/4)	75.0 (3/4)	25.0 (1/4)	0.0 (0/4)
**DSI and DH**	7.9 (44/556)	43.2 (19/44)	18.2 (8/44)	31.8 (14/44)	75.0 (33/44)	4.5 (2/44)	22.7 (10/44)	45.5 (20/44)	4.5 (2/44)	43.2 (19/44)	2.3 (1/44)	11.4 (5/44)	25.0 (11/44)	43.2 (19/44)	25.0 (11/44)	6.8 (3/44)
**DSI and/or DH and/or disc bulging and herniation**	4.1 (23/556)	60.9 (14/23)	30.4 (7/23)	13.0 (3/23)	43.5 (10/23)	0.0 (0/23)	56.5 (13/23)	34.8 (8/23)	8.7 (2/23)	34.8 (8/23)	8.7 (2/23)	21.7 (5/23)	17.4 (4/23)	60.9 (14/23)	0.0 (0/23)	21.7 (5/23)
**DSI and/or DH and/or structural changes, and distinction between AF and NP**	77.5 (431/556)	40.4 (174/431)	23.9 (103/431)	32.9 (142/431)	52.4 (226/431)	11.1 (60/431)	33.6 (145/431)	61.3 (264/431)	6.5 (28/431)	9.0 (39/431)	3.7 (16/431)	23.0 (99/431)	5.6 (24/431)	61.3 (264/431)	31.3 (135/431)	1.9 (8/431)
**DSI and/or DH and/or osteophytes, end‐plate changes, Modic changes and high intensity zones**	5.9 (33/556)	42.4 (14/33)	27.3 (9/33)	30.3 (10/33)	51.5 (17/33)	9.1 (3/33)	39.4 (13/33)	42.4 (14/33)	0.0 (0/33)	42.4 (14/33)	3.0 (1/33)	21.2 (7/33)	39.4 (13/33)	48.5 (16/33)	9.1 (3/33)	3.0 (1/33)
**Quantitative grading systems (all)**	**16.8 (112/668)**	**33.0** **(37/112)**	**11.6** **(13/112)**	**54.5** **(61/112)**	**60.7** **(68/112)**	**3.6** **(4/112)**	**35.7** **(40/112)**	**76.8** **(86/112)**	**2.7** **(3/112)**	**3.6** **(4/112)**	**6.3** **(7/112)**	**13.4 (15/112)**	**92.0 (103/112)**	**6.3** **(7/112)**	**0.0** **(0/112)**	**3.6** **(4/122)**
**DSI**	17.9 (20/112)	25.0 (5/20)	20.0 (4/20)	65.0 (13/20)	50.0 (10/20)	5.0 (1/20)	45.0 (9/20)	65.0 (13/20)	5.0 (1/20)	0.0 (0/20)	15.0 (3/20)	25.0 (5/20)	75.0 (15/20)	15.0 (3/20)	0.0 (0/20)	10.0 (2/20)
**DH**	2.7 (3/112)	0.0 (0/3)	0.0 (0/3)	100.0 (3/3)	66.7 (2/3)	0.0 (0/3)	33.3 (1/3)	100.0 (3/3)	0.0 (0/3)	0.0 (0/3)	33.3 (1/3)	0.0 (0/3)	100.0 (3/3)	0.0 (0/3)	0.0 (0/3)	0.0 (0/3)
**Disc bulging**	3.6 (4/112)	75.0 (3/4)	0.0 (0/4)	25.0 (1/4)	25.0 (1/4)	0.0 (0/4)	75.0 (3/4)	50.0 (2/4)	0.0 (0/4)	0.0 (0/4)	25.0 (1/4)	25.0 (1/4)	50.0 (2/4)	0.0 (0/4)	0.0 (0/4)	50.0 (2/4)
**DSI and DH**	2.7 (3/112)	66.7 (2/3)	33.3 (1/3)	0.0 (0/3)	66.7 (2/3)	0.0 (0/3)	33.3 (1/3)	66.7 (2/3)	0.0 (0/3)	33.3 (1/3)	0.0 (0/3)	0.0 (0/3)	100.0 (3/3)	0.0 (0/3)	0.0 (0/3)	0.0 (0/3)
**DSI, DH, and disc bulging**	8.9 (10/112)	20.0 (2/10)	20.0 (2/10)	30.0 (3/10)	80.0 (8/10)	10.0 (1/10)	10.0 (1/10)	70.0 (7/10)	0.0 (0/10)	10.0 (1/10)	0.0 (0/10)	20.0 (2/10)	80.0 (8/10)	20.0 (2/10)	0.0 (0/10)	0.0 (0/10)
**Specialized quantitative MRI techniques and sequences**	64.3 (72/112)	34.8 (25/72)	8.3 (6/72)	56.9 (41/72)	62.5 (45/72)	2.8 (2/72)	34.7 (25/72)	81.9 (59/72)	2.8 (2/72)	2.8 (2/72)	2.8 (2/72)	9.7 (7/72)	97.2 (70/72)	2.8 (2/72)	0.0 (0/72)	0.0 (0/72)
**Summary of subjective and quantitative grading systems**	**668**	**40.9 (273/668)**	**22.0 (147/668)**	**35.6 (238/668)**	**54.5 (364/668)**	**11.1 (74/668)**	**34.6 (231/668)**	**61.2 (409/668)**	**5.5 (37/668)**	**12.6 (84/668)**	**4.2 (28/668)**	**20.4 (136/668)**	**23.2 (155/668)**	**49.7 (332/668)**	**22.9 (153/668)**	**4.5 (30/668)**

Abbreviations: AF, annulus fibrosis; DD, disc degeneration; DH, disc height; DSI, disc signal intensity; LBP, low back pain; MRI, magnetic resonance imaging; NP, nucleus pulposus.

^a^ Included combinations of T12‐L5, T12‐S1, L1‐L5, and L5‐S1. Other category includes unspecified and all other combinations reported.

^b^ The total number of responses may exceed the number of reports of grading system use due to the possibility of multiple options.

Of studies reporting the use of quantitative grading systems (*n* = 112), specialized quantitative MRI techniques and sequences (e.g., T2 mapping, T1 relaxation) were most commonly used (72/112, 64.3%). Ten grading systems, in 17.9% (20/112) of reports of quantitative grading systems, utilized measurements of DSI to grade DD, and six grading systems, in 8.9% (10/112) of reports of quantitative grading systems, used a combination of quantitative DSI, DH, and disc bulging.

### 3.3 Methods Used to Assess and Report the Degree of Disc Degeneration

The methods of synthesis used to report DD grading are presented in Table 1. Radiologists most commonly performed the assessment of DD (273/668, 40.9% across all reports and 236/556, 42.4% for subjective grading systems). However, for the reports of the use of quantitative grading systems, the grader was mostly unspecified (61/112, 54.5%). Disc degeneration was usually assessed across all lumbar spine levels (364/668, 54.5%) for both subjective and quantitative grading systems.

A number of different methods were used to synthesize the DD findings for analysis. The grading systems were commonly analyzed at each individual level (409/668, 61.2%) regardless of the type of grading system used. For subjective grading systems, results across multiple levels were sometimes synthesized as the sum of all the levels (80/556, 14.4%) or as the worst score at any level (34/556, 6.1%). It was uncommon for quantitative grading systems to analyze DD using the worst level (3/112, 2.7%), the sum of all levels (4/112, 3.6%) or the average across all levels (7/112, 6.3%). Of the 183 reports of grading systems using dichotomous summary measures, almost all used a subjective grading system (179/183, 97.8%) and collected the data at an ordinal level before transforming it into a dichotomous variable at each level (153/183, 83.6%). See Supporting Information S5 for more detail.

### 3.4 Assessment of the Measurement Properties of the Grading Systems

The measurement properties that were assessed for the various MRI‐based grading systems are presented in Table 2. Intra‐rater (204/668, 30.5%) and inter‐rater reliability (232/668, 34.7%) were commonly reported across both subjective and quantitative grading systems. Of the 93 grading systems identified, 33.3% (31/93) had not been assessed for any type of reliability. Sensitivity to change was rarely reported for subjective (61/556, 11.0%) or quantitative grading systems (11/112, 9.8%).

**TABLE 2:** The proportion of grading systems reported to be assessed for measurement properties, stratified by the grading system components used to assess for degenerative disc disease.

		Reliability		Sensitivity to change		Validity	
Grading system components	Proportion of reported use of grading systems % (n/N)	Intra‐rater reliability % (n/N)	Inter‐rater reliability % (n/N)	Use of a change score % (n/N)	Comparative evaluation with another grading system % (n/N)	Measured associations between DD and other variables % (n/N)	Measured associations between DD and LBP % (n/N)
**Subjective grading systems (all)**	**83.2** **(556/668)**	**28.1 (156/556)**	**34.5 (192/556)**	**11.0 (61/556)**	**14.6 (81/556)**	**46.2 (257/556)**	**18.0 (100/556)**
DSI	3.8 (21/556)	23.8 (5/21)	33.3 (7/21)	0.0 (0/21)	9.5 (2/21)	47.6 (10/21)	28.6 (6/21)
DH	0.7 (4/556)	25.0 (1/4)	50.0 (2/4)	0.0 (0/4)	0.0 (0/4)	75.0 (3/4)	50.0 (2/4)
DSI and DH	7.9 (44/556)	31.8 (14/44)	45.5 (20/44)	11.4 (5/44)	11.4 (5/44)	54.5 (24/44)	31.8 (14/44)
DSI and/or DH and/or disc bulging and herniation	4.1 (23/556)	30.4 (7/23)	39.1 (9/23)	13.0 (3/23)	17.4 (4/23)	52.2 (12/23)	34.8 (8/23)
DSI and/or DH and/or structural changes, and distinction between AF and NP	77.5 (431/556)	26.7 (115/431)	33.4 (144/431)	10.9 (47/431)	16.0 (69/431)	43.6 (188/431)	13.9 (60/431)
DSI and/or DH and/or osteophytes, end‐plate changes, Modic changes and high intensity zones	5.9 (33/556)	42.4 (14/33)	30.3 (10/33)	18.2 (6/33)	3.0 (1/33)	60.6 (20/33)	30.3 (10/33)
**Quantitative grading systems (all)**	**16.8 (112/668)**	**42.9 (48/112)**	**35.7 (40/112)**	**9.8 (11/112)**	**61.6 (69/112)**	**33.9 (38/112)**	**10.7 (12/112)**
DSI	17.9 (20/112)	30.0 (6/20)	20.0 (4/20)	25.0 (5/20)	15.0 (3/20)	55.0 (11/20)	25.0 (5/20)
DH	2.7 (3/112)	33.3 (1/3)	0.0 (0/3)	0.0 (0/3)	33.3 (1/3)	66.7 (2/3)	0.0 (0/3)
Disc bulging	3.6 4/112)	75.0 (3/4)	50.0 (2/4)	0.0 (0/4)	0.0 (0/4)	100.0 (4/4)	50.0 (2/4)
DSI and DH	2.7 (3/112)	0.0 (0/3)	33.3 (1/3)	33.3 (1/3)	33.3 (1/3)	66.7 (2/3)	33.3 (1/3)
DSI, DH, and disc bulging	8.9 (10/112)	70.0 (7/10)	70.0 (7/10)	20.0 (2/10)	20.0 (2/10)	60.0 (6/10)	10.0 (1/10)
Specialized quantitative MRI techniques and sequences	64.3 (72/112)	43.1 (31/72)	36.1 (26/72)	4.2 (3/72)	86.1 (62/72)	18.1 (13/72)	4.2 (3/72)
**Summary of subjective and quantitative grading systems**	**668**	**30.5 (204/668)**	**34.7 (232/668)**	**10.8 (72/668)**	**22.5 (150/668)**	**44.2 (295/668)**	**16.8 (112/668)**

Abbreviations: AF, annulus fibrosis; DD, disc degeneration; DH, disc height; DSI, disc signal intensity; LBP, low back pain; MRI, magnetic resonance imaging; NP, nucleus pulposus.

Validity was the most commonly reported measurement property assessed. In subjective grading systems, just under half (257/556, 46.2%) reported associations between DD and other variables including other imaging findings (e.g., degenerative spondylolisthesis, adolescent scoliosis, and Modic changes) and patient‐level data (e.g., age, occupation and genetic factors). While it was less common for quantitative grading systems to measure associations with other variables (38/112, 33.9%), reports of quantitative grading systems were more commonly assessed for validity using a comparative evaluation with another grading system at a single disc level (69/112, 61.6%). The association between LBP and DD was investigated in 16.8% (112/668) of the reports of grading system use. More specifically, 83/668 (12.4%) of reports investigated the association between DD and current LBP, and 29/668 (4.3%) with future LBP. Subjective grading systems were more commonly used to investigate associations between DD and LBP (100/112, 89.2%) when compared with quantitative grading systems (12/112, 10.7%). See Supporting Information S6 for more detail.

## 4 Discussion

### 4.1 Key Findings

This scoping review comprehensively charted the MRI‐based grading systems that measure lumbar DD. We identified 569 studies that reported using MRI‐based grading systems to assess for DD. Ninety‐three different grading systems were identified, including 63 subjective systems, 25 quantitative systems, and 5 that were unspecified. The subjective MRI‐based grading system proposed by Pfirrmann [10] was used more than half the time. Many grading systems (60/93, 64.5%) were only reported once.

There was substantial heterogeneity in the components used to grade DD. Subjective grading systems most commonly used combinations of DSI, DH, structural changes, and the distinctiveness of the annulus‐nucleus boundary to grade DD, while quantitative grading systems commonly used specialized quantitative MRI techniques and sequences.

A variety of measurement properties of the grading systems were assessed. Intra‐rater and inter‐rater reliability were assessed in approximately one‐third of reports. Thirty‐one of the total 93 grading systems were not assessed for any form of reliability. With regard to validity, studies that used subjective grading systems commonly reported measured associations between DD and other clinical variables such as other imaging findings (degenerative spondylolisthesis, adolescent scoliosis, and Modic changes) and patient‐level data (age, occupation, and genetic factors). Studies that used quantitative grading systems were more likely to report a comparative evaluation with another grading system or imaging modality at a single disc level. When the association between DD and LBP was assessed, most studies used a subjective grading system and assessed the association with current LBP. Sensitivity to change was rarely assessed. Studies investigating sensitivity to change were longitudinal in nature and required repeated imaging over time. These are more resource‐intensive, which may explain their scarcity compared to cross‐sectional designs.

### 4.2 Comparison to Previous Literature

To the authors' knowledge, there are no previous scoping reviews that map the literature on MRI‐based grading systems for DD in the lumbar spine. A previous systematic review was conducted to identify and evaluate a range of different grading systems for cervical and lumbar degeneration in the disc and facet joints [26]. Unlike our study, many different imaging modalities were considered, including macroscopic, histological, plain radiography, MRI, and discography [26]. The review found five different grading systems that measured lumbar DD on MRI [26]. A substantially smaller number of grading systems were identified compared to our study, as only studies presenting the original grading system were included in the review. Furthermore, only statistically recommended grading systems were additionally evaluated with respect to their clinical feasibility, clinical relevance, and academic value in the previous review [25]. This explained only some of the difference in the number of grading systems identified.

A scoping review of grading systems for lumbar facet joints on MRI was conducted by Acosta [27] to map the grading systems used to assess inflammatory changes to the lumbar facet joints. Like our study, it found a large variation in the components and scales used to grade facet inflammation. The review identified six grading systems, which had undergone assessment of reliability [27].

### 4.3 Strengths and Limitations

The key strength of this study was the inclusive nature of the methodological design. As part of the scoping review, a wide spectrum of grading systems was identified and included in the analysis. Specifically, our study identified grading systems regardless of whether the system had been evaluated for any measurement properties. The inclusion criteria included any subjective or quantitative system that described the presence or absence of disc degeneration or the degree/extent of DD, and therefore focused on the reported use of DD grading systems to more clearly map which grading systems were most commonly used.

One of the limitations of the study was the challenge in defining when a study was considered to have used a grading system to measure DD and therefore met our inclusion criteria. Studies were only included if it was explicitly stated that DD (or a similar term) was being assessed. Some studies described changes to the disc (e.g., disc herniation) without clearly stating that the changes measured were for the purposes of measuring DD. Therefore, this may have resulted in some grading systems being omitted from the review.

Categorizing the specialized quantitative MRI techniques and sequences used to grade DD into more specific categories was challenging. As these specialized quantitative MRI techniques and sequences were commonly used, some nuances regarding how these systems are reported and measured may have been lost by combining them.

### 4.4 Implications for Future Research

A large number of grading systems were identified in this review, many of which have been infrequently used or assessed. There was substantial heterogeneity in the components used in the grading systems, the thresholds for determining the presence of DD, and the method of synthesis. As a result, the comparison of results across different studies is difficult and may impact the way the grading system is used when making associations with LBP. For example, of those studies using the Pfirrmann method [10], 46 studies used a grade higher than three to dichotomize the presence of DD at a single level, while 44 studies used a grade higher than two. In five studies, a grade higher than one was used to demarcate the presence of DD. A more standardized threshold is recommended for systems like the Pfirrmann [10] method when being used to measure for associations with clinical variables such as LBP.

Some of the observed variation in the method of synthesis may also be due to study‐specific aims and study designs. For example, if DD was compared to a patient‐level outcome, such as LBP, a summary measure across disc levels may be required, whereas comparisons between two alternative grading systems may be assessed at the individual disc level. A wide range of approaches were taken to calculate a summary measure across disc levels, including using the sum of all levels, averaging across all levels, and the worst level in different studies. Using different summary measures to make associations with LBP may also impact the accuracy of these associations. Given that different summary measures are used in a variety of study designs and for a range of different aims, choosing the appropriate method of synthesis may also contribute to the generation of a more robust association between DD and LBP.

The choice between using a subjective or quantitative grading system comes with an inherent trade‐off between reliability and ease of use. For example, subjective systems are widely used due to their simplicity and rapid application; however, they are limited primarily by their insufficient discriminatory capacity and relatively poor inter‐rater reliability [14]. In contrast, quantitative grading systems are a more reliable measure of DD, as they can be used to measure changes to the intervertebral disc more objectively [15]. However, these techniques often require specialized software, additional time for analysis, and may fail to account for inter‐patient variability.

Advances in artificial intelligence and machine learning could address current limitations by automating grading, reducing the subjectivity of reporting, and improving normalization for confounding variables (e.g., age, disc level, etc.). Future developments in AI‐assisted grading could improve the efficiency and reliability of how these grading systems are used.

There were no quantitative grading systems that were identified in this review that systematically normalized DD scores for patient‐level factors such as age and disc level. There is preliminary evidence to suggest that normalized quantitative measures of DSI and DH may measure the degenerative process more accurately compared to the raw non‐normalized quantitative measures [28]. Further research is required to investigate the association between normalized quantitative measures and LBP [28].

## 5 Conclusion

In this review, we identified a large number of grading systems, many of which were infrequently used. In total, 93 MRI‐based grading systems for assessing lumbar DD were identified, including 63 subjective grading systems, 25 quantitative grading systems, and five that were unspecified. Subjective grading systems were widely utilized, with the Pfirrmann method used in over 50% of reports. A significant number of grading systems were only reported in single studies. There was substantial heterogeneity in the components used in the grading systems; however, the most common grading components were DSI, DH, and the distinctiveness of the annulus‐nucleus boundary. There were also significant differences in the methods of synthesis used across studies. The measurement properties of the grading systems (such as reliability) were commonly assessed across the grading systems, while sensitivity to change was rarely examined. When an association with LBP was made, it was usually between a subjective grading system and current LBP. The variability described in both the components used to measure DD and the methods used to synthesize the data may hinder the ability to draw clear associations with LBP.

## Author Contributions

**Dean Esposito:** substantial contribution to the study design, analysis and interpretation of data, and drafting the paper or revising it critically. **Mark Jonathan Hancock:** substantial contribution to the study design, data acquisition, analysis and interpretation of data, and drafting the paper and revising it critically. **Benjamin Brown:** substantial contribution to the study design, analysis and interpretation of data and drafting the paper, and revising it critically. **Samuel Stuart Graham King:** substantial contribution to the study design, data acquisition, and drafting of the paper and revising it critically. **Isaac Gerard Tom Searant:** substantial contribution to data acquisition and drafting of the paper, and revising it critically. **Hazel Jenkins:** substantial contribution to the study design, interpretation of data and drafting the paper and revising it critically. All authors have read and approved the final version of the manuscript to be published.

## Conflicts of Interest

The authors declare no conflicts of interest.

## Supporting information


**Data S1:** Supporting Information.

**Data S2:** Supporting Information.

**Data S3:** Supporting Information.

**Data S4:** Supporting Information.

**Data S5:** Supporting Information.

**Data S6:** Supporting Information.
