# Supplementary material for: MRI‐Based Grading Systems for Assessing Lumbar Disc Degeneration: A Scoping Review
Source: JOR Spine. 2025 Sep 15;8(3):e70113. doi: 10.1002/jsp2.70113 (PMC12435304; doi:10.1002/jsp2.70113)
Supplement: Supplementary file 1 — Data S1: Supporting Information. [file JSP2-8-e70113-s003.docx]

**Online Resource 1: Search strategy**

For each database search terms were used for each of the three key domains: magnetic resonance imaging; intervertebral disc degeneration; lumbar vertebrae

Terms with each of the domains were combined with ‘or’

The four key domains were combined with ‘and’

**Search Terms**

**Medline**

1 magnetic resonance imaging/

2 magnetic resonance imaging.mp.

3 magnetic resonance.mp.

4 MRI findings.mp.

5 MR imaging.mp.

6 MRI.mp.

7 1 or 2 or 3 or 4 or 5 or 6

8 intervertebral disc degeneration/

9 intervertebral disc degeneration.mp.

10 intervertebral disk degeneration.mp.

11 ((disc or disk) adj3 (degenerat* or degradat* or disease*)).mp.

12 degenera* disc.mp.

13 degenerat* disk.mp.

14 Disc signal intensity.mp.

15 Disk signal intensity.mp.

16 disc height.mp.

17 disk height.mp.

18 spondylosis.mp.

19 8 or 9 or 10 or 11 or 12 or 13 or 14 or 15 or 16 or 17 or 18

20 lumbar vertebrae/

21 lumbar vertebrae.mp.

22 lumbar vertebra.mp.

23 (lumbar adj2 (spine or vertebrae)).mp.

24 Low back pain.mp.

25 LBP.mp.

26 20 or 21 or 22 or 23 or 24 or 25

27 7 and 19 and 26

**EMBASE**

1 nuclear magnetic resonance imaging/

2 magnetic resonance imaging.mp.

3 magnetic resonance.mp.

4 MRI findings.mp.

5 MR imaging.mp.

6 MRI.mp.

7 1 or 2 or 3 or 4 or 5 or 6

8 intervertebral disk degeneration/

9 intervertebral disc degeneration.mp.

10 intervertebral disk degeneration.mp.

11 ((disc or disk) adj3 (degenerat* or degradat* or disease*)).mp.

12 degenera* disc.mp.

13 degenerat* disk.mp.

14 Disc signal intensity.mp.

15 Disk signal intensity.mp.

16 disc height.mp.

17 disk height.mp.

18 spondylosis.mp.

19 8 or 9 or 10 or 11 or 12 or 13 or 14 or 15 or 16 or 17 or 18

20 lumbar vertebra/

21 lumbar vertebrae.mp.

22 lumbar vertebra.mp.

23 (lumbar adj2 (spine or vertebrae)).mp.

24 Low back pain.mp.

25 LBP.mp.

26 20 or 21 or 22 or 23 or 24 or 25

27 7 and 19 and 26

**CINAHL**

S1 (MH "Magnetic Resonance Imaging")

S2 (TI "magnetic resonance imaging" or AB "magnetic resonance imaging")

S3 (TI "MR imaging" or AB "MR imaging")

S4 (TI MRI or AB MRI)

S5 (TI "Magnetic resonance" or AB "magnetic resonance

S6 S1 OR S2 OR S3 OR S4 OR S5

S7 TI (disk or disc) N3 (degenerat* or degrad* or disease*) or AB (disk or disc) N3 (degenerat* or degrad* or disease*)

S8 (TI "degenera* disc" or ab "degenera* disc)

S9 (TI "degenerat* disk" or AB "degenerat* disk")

S10 (TI "intervertebral disc degeneration" or AB "intervertebral disc degeneration")

S11 (TI "disc changes" or AB "disc changes" or (TI "disk changes" or AB "disk changes")

S12 (TI "disc signal intensity" or AB "disc signal intensity") or (TI "disc height" or AB "disc height") or (TI "disk height" or AB "disk height")

S13 S7 OR S8 OR S9 OR S10 OR S11 OR S12

S14 (MH "Lumbar Vertebrae")

S15 (TI (lumbar) N2 (spine or vertebrae) or AB (lumbar) N2 (spine or vertebrae)

S16 (TI LBP or AB LBP) or (TI "low back pain" or AB "low back pain")

S17 S14 OR S15 OR S16

S18 S6 AND S13 AND S17
