# Supplementary material for: MRI‐Based Grading Systems for Assessing Lumbar Disc Degeneration: A Scoping Review
Source: JOR Spine. 2025 Sep 15;8(3):e70113. doi: 10.1002/jsp2.70113 (PMC12435304; doi:10.1002/jsp2.70113)
Supplement: Supplementary file 2 — Data S2: Supporting Information. [file JSP2-8-e70113-s001.docx]

**Online Resource 2. Complete list of references for all 569 studies included in scoping review**

| **Grading system name** | **References** |
| --- | --- |
| **Subjective grading systems** | [1-4] [5-7] [8, 9] [10-80] [49, 50, 81-86] [51, 87-332] [74-76, 99, 178, 212, 333-509] [447, 449, 510-540] |
| Disc signal intensity | [1-21] |
| Gibson | [1-4] |
| Decandido | [5-7] |
| Luoma | [8, 9] |
| Other | [10-21] |
| Disc height | [11, 12, 20, 541] |
| Disc height and disc signal intensity | [22-51, 56-69] |
| Schneidermann | [22-51] |
| Jensen | [56-60] |
| Luoma | [61, 62] |
| Other | [63-69] |
| DSI and/or DH and/or disc bulging and herniation | [49, 50, 52-55, 70-86] |
| Fardon | [70-73] |
| Solovieva | [52-55] |
| Witwit | [74-76] |
| Battie | [77, 78] |
| Horton and Daftari | [79, 80] |
| Kanamori | [49, 50] |
| Videman | [81, 82] |
| Other | [83-86] |
| DSI and/or DH and/or herniation, structural changes, and distinction between annulus fibrosis and nucleus fibrosus | [51, 87-332] [74-76, 333-452] [99, 178, 212, 434, 453-490] [491-499] [500-502] [503, 504] [503, 504] [505, 506] [507-509] |
| Pfirrmann | [51, 87-332] [74-76, 333-452] |
| Modified Pfirrmann | [99, 178, 212, 434, 453-490] |
| Thompson | [491-499] |
| Buirski | [500-502] |
| Modified Pearce | [503, 504] |
| Woodend Classification | [505, 506] |
| Other | [507-509] |
| DSI and/or DH and/or osteophytes, end-plate changes, Modic changes and high intensity zones (HIZ) | [447, 449, 510-540] |
| Jarosz Atlas | [447, 449, 510-519] |
| Pearce | [520-525] |
| Battie | [526, 527] |
| Benneker | [528, 529] |
| Tuft degenerative disc classification | [530, 531] |
| Other | [532-540] |
| **Quantitative grading systems** | [8, 9, 22, 62, 77, 78, 81, 83, 89, 222, 315, 350, 407, 452, 483, 527, 528, 536, 541-559] [23, 24, 51, 73, 92, 104, 106, 113, 114, 119, 120, 122, 137, 162, 165, 170, 172, 185, 186, 199, 212, 222, 225, 226, 228, 254, 261, 264, 270, 271, 275-277, 286, 293, 295, 299, 317, 318, 341, 360, 369, 375, 376, 402, 409, 418-420, 423, 426, 431, 433, 437, 441, 442, 445, 448, 450, 451, 455, 463, 469, 475, 477, 487, 508, 560-564] |
| Disc signal intensity | [22, 78, 81, 83, 89, 222, 315, 407, 483, 541-551] |
| Videman | [78, 81, 541-543] |
| Paajanen | [544-547] |
| Battie | [83, 548] |
| Luoma | [483, 549] |
| Nagashima | [315, 550] |
| Other | [22, 89, 222, 407, 551] |
| Disc height | [222, 548, 549] |
| Disc bulging | [8, 9, 62, 552] |
| Luoma | [8, 9, 62] |
| Other | [552] |
| Disc signal intensity and disc height | [350, 528, 553] |
| Disc signal intensity, disc height, and disc bulging | [77, 452, 527, 536, 554-559] |
| Battie | [77, 527, 554] |
| Feng | [555-557] |
| Other | [452, 536, 558, 559] |
| Specialised quantitative MRI techniques and sequences | [23, 24, 51, 73, 92, 104, 106, 113, 114, 119, 120, 122, 137, 162, 165, 170, 172, 185, 186, 199, 212, 222, 225, 226, 228, 254, 261, 264, 270, 271, 275-277, 286, 293, 295, 299, 317, 318, 341, 360, 369, 375, 376, 402, 409, 418-420, 423, 426, 431, 433, 437, 441, 442, 445, 448, 450, 451, 455, 463, 469, 475, 477, 487, 508, 560-564] |
| Unspecified | [565-569] |

**Reference List**

1. Dai LY. Orientation and Tropism of Lumbar Facet Joints in Degenerative Spondylolisthesis. *International Orthopaedics*. 2001;25(1):40-2.

2. Gibson MJ, Buckley J, Mawhinney R, Mulholland RC, Worthington BS. Magnetic Resonance Imaging and Discography in the Diagnosis of Disc Degeneration. A Comparative Study of 50 Discs. *Journal of Bone and Joint Surgery - Series B*. 1986;68(3):369-373. doi:https://dx.doi.org/10.1302/0301-620x.68b3.3733797

3. Hyodo H, Sato T, Sasaki H, Tanaka Y. Discogenic Pain in Acute Nonspecific Low-Back Pain. *European Spine Journal*. Aug 2005;14(6):573-7.

4. Dai LY. Disc Degeneration in Patients with Lumbar Spondylolysis. *Journal of Spinal Disorders*. Dec 2000;13(6):478-86.

5. Decandido P, Reinig JW, Dwyer AJ, Thompson KJ, Ducker TB. Magnetic Resonance Assessment of the Distribution of Lumbar Spine Disc Degenerative Changes. *Journal of Spinal Disorders*. 1988;1(1):9-15.

6. Dimitriadis A, Smith F, Mavrogenis AF, Pope MH, Papagelopoulos PJ, Karantanas A, et al. Effect of Two Sitting Postures on Lumbar Sagittal Alignment and Intervertebral Discs in Runners. *Radiologia Medica*. Jun 2012;117(4):654-68. doi:https://dx.doi.org/10.1007/s11547-011-0748-8

7. Iida T, Abumi K, Kotani Y, Kaneda K. Effects of Aging and Spinal Degeneration on Mechanical Properties of Lumbar Supraspinous and Interspinous Ligaments. *Spine Journal: Official Journal of the North American Spine Society*. Mar-Apr 2002;2(2):95-100.

8. Luoma K, Riihimaki H, Luukkonen R, Raininko R, Viikari-Juntura E, Lamminen A. Low Back Pain in Relation to Lumbar Disc Degeneration. Research Support, Non-U.S. Gov't. *Spine*. Feb 15 2000;25(4):487-92.

9. Luoma K, Riihimaki H, Raininko R, Luukkonen R, Lamminen A, Viikari-Juntura E. Lumbar Disc Degeneration in Relation to Occupation. Comparative Study Research Support, Non-U.S. Gov't. *Scandinavian Journal of Work, Environment & Health*. Oct 1998;24(5):358-66.

10. Dimar JR, 2nd, Glassman SD, Carreon LY. Juvenile Degenerative Disc Disease: A Report of 76 Cases Identified by Magnetic Resonance Imaging. Research Support, Non-U.S. Gov't. *Spine Journal: Official Journal of the North American Spine Society*. May-Jun 2007;7(3):332-7.

11. Dragsbaek L, Kjaer P, Hancock M, Jensen TS. An Exploratory Study of Different Definitions and Thresholds for Lumbar Disc Degeneration Assessed by Mri and Their Associations with Low Back Pain Using Data from a Cohort Study of a General Population. *BMC Musculoskeletal Disorders*. Apr 17 2020;21(1):253. doi:https://dx.doi.org/10.1186/s12891-020-03268-4

12. Fu MC, Buerba RA, Long WD, 3rd, Blizzard DJ, Lischuk AW, Haims AH, et al. Interrater and Intrarater Agreements of Magnetic Resonance Imaging Findings in the Lumbar Spine: Significant Variability across Degenerative Conditions. *Spine Journal: Official Journal of the North American Spine Society*. Oct 01 2014;14(10):2442-8. doi:https://dx.doi.org/10.1016/j.spinee.2014.03.010

13. Kotilainen E, Alanen A, Erkintalo M, Valtonen S, Kormano M. Association between Decreased Disc Signal Intensity in Preoperative T2-Weighted Mri and a 5-Year Outcome after Lumbar Minimally Invasive Discectomy. Research Support, Non-U.S. Gov't. *Minimally Invasive Neurosurgery*. Mar 2001;44(1):31-6.

14. Linson MA, Crowe CH. Comparison of Magnetic Resonance Imaging and Lumbar Discography in the Diagnosis of Disc Degeneration. Comparative Study. *Clinical Orthopaedics & Related Research*. Jan 1990;(250):160-3.

15. Liuke M, Solovieva S, Lamminen A, Luoma K, Leino-Arjas P, Luukkonen R, et al. Disc Degeneration of the Lumbar Spine in Relation to Overweight. Research Support, Non-U.S. Gov't. *International Journal of Obesity*. Aug 2005;29(8):903-8.

16. Madan SS, Rai A, Harley JM. Interobserver Error in Interpretation of the Radiographs for Degeneration of the Lumbar Spine. Comparative Study. *Iowa Orthopaedic Journal*. 2003;23:51-6.

17. Tertti M, Paajanen H, Kujala UM, Alanen A, Salmi TT, Kormano M. Disc Degeneration in Young Gymnasts. A Magnetic Resonance Imaging Study. *American Journal of Sports Medicine*. Mar-Apr 1990;18(2):206-8.

18. Heithoff KB, Gundry CR, Burton CV, Winter RB, Heithoff KB, Gundry CR, et al. Juvenile Discogenic Disease. *Spine (03622436)*. 1994;19(3):335-340.

19. Evans W, Jobe W, Seibert C, Evans W, Jobe W, Seibert C. A Cross-Sectional Prevalence Study of Lumbar Disc Degeneration in a Working Population. *Spine (03622436)*. 1989;14(1):60-64.

20. Ito M, Incorvaia KM, Yu SF, Fredrickson BE, Yuan HA, Rosenbaum AE. Predictive Signs of Discogenic Lumbar Pain on Magnetic Resonance Imaging with Discography Correlation. Comparative Study. *Spine*. Jun 01 1998;23(11):1252-8; discussion 1259-60.

21. Maurer M, Soder RB, Baldisserotto M. Spine Abnormalities Depicted by Magnetic Resonance Imaging in Adolescent Rowers. Comparative Study. *American Journal of Sports Medicine*. Feb 2011;39(2):392-7. doi:https://dx.doi.org/10.1177/0363546510381365

22. Lund T, Schlenzka D, Lohman M, Ristolainen L, Kautiainen H, Klemetti E, et al. The Intervertebral Disc During Growth: Signal Intensity Changes on Magnetic Resonance Imaging and Their Relevance to Low Back Pain. Research Support, Non-U.S. Gov't. *PLoS ONE [Electronic Resource]*. 2022;17(10):e0275315. doi:https://dx.doi.org/10.1371/journal.pone.0275315

23. Pang H, Bow C, Cheung JPY, Zehra U, Borthakur A, Karppinen J, et al. The Ute Disc Sign on Mri: A Novel Imaging Biomarker Associated with Degenerative Spine Changes, Low Back Pain, and Disability. *Spine (03622436)*. 2017;42(15):N.PAG-N.PAG. doi:10.1097/BRS.0000000000002369

24. Pang H, Bow C, Cheung JPY, Zehra U, Borthakur A, Karppinen J, et al. The Ute Disc Sign on Mri: A Novel Imaging Biomarker Associated with Degenerative Spine Changes, Low Back Pain, and Disability. *Spine*. 04 01 2018;43(7):503-511. doi:https://dx.doi.org/10.1097/BRS.0000000000002369

25. Bakr KI, Sadiq IM, Nooruldeen SA. Lumbosacral Mri Findings in Chronic Lower Back Pain. *Indian Journal of Public Health Research and Development*. November 2019;10(11):2035-2040. doi:https://dx.doi.org/10.5958/0976-5506.2019.03856.7

26. Cheung KM, Chan D, Karppinen J, Chen Y, Jim JJ, Yip SP, et al. Association of the Taq I Allele in Vitamin D Receptor with Degenerative Disc Disease and Disc Bulge in a Chinese Population. Multicenter Study Research Support, Non-U.S. Gov't. *Spine*. May 01 2006;31(10):1143-8.

27. Cheung KM, Samartzis D, Karppinen J, Mok FP, Ho DW, Fong DY, et al. Intervertebral Disc Degeneration: New Insights Based on "Skipped" Level Disc Pathology. Research Support, Non-U.S. Gov't. *Arthritis & Rheumatism*. Aug 2010;62(8):2392-400. doi:https://dx.doi.org/10.1002/art.27523

28. Cheung KMC, Karppinen J, Chan D, Ho DWH, Song YQ, Sham P, et al. Prevalence and Pattern of Lumbar Magnetic Resonance Imaging Changes in a Population Study of One Thousand Forty-Three Individuals. *Spine*. 20 Apr 2009;34(9):934-940. doi:https://dx.doi.org/10.1097/BRS.0b013e3181a01b3f

29. Higashino K, Matsui Y, Yagi S, Takata Y, Goto T, Sakai T, et al. The Alpha2 Type Ix Collagen Tryptophan Polymorphism Is Associated with the Severity of Disc Degeneration in Younger Patients with Herniated Nucleus Pulposus of the Lumbar Spine. Research Support, Non-U.S. Gov't. *International Orthopaedics*. Feb 2007;31(1):107-11.

30. Kanayama M, Togawa D, Takahashi C, Terai T, Hashimoto T. Cross-Sectional Magnetic Resonance Imaging Study of Lumbar Disc Degeneration in 200 Healthy Individuals. *Journal of Neurosurgery Spine*. Oct 2009;11(4):501-7. doi:https://dx.doi.org/10.3171/2009.5.SPINE08675

31. Kawaguchi Y, Kanamori M, Ishihara H, Ohmori K, Matsui H, Kimura T. The Association of Lumbar Disc Disease with Vitamin-D Receptor Gene Polymorphism. Research Support, Non-U.S. Gov't. *Journal of Bone & Joint Surgery - American Volume*. Nov 2002;84(11):2022-8.

32. Kawaguchi Y, Osada R, Kanamori M, Ishihara H, Ohmori K, Matsui H, et al. Association between an Aggrecan Gene Polymorphism and Lumbar Disc Degeneration. *Spine*. Dec 01 1999;24(23):2456-60.

33. Law T, Anthony MP, Chan Q, Samartzis D, Kim M, Cheung KMC, et al. Ultrashort Time-to-Echo Mri of the Cartilaginous Endplate: Technique and Association with Intervertebral Disc Degeneration. *Journal of Medical Imaging and Radiation Oncology*. August 2013;57(4):427-434. doi:https://dx.doi.org/10.1111/1754-9485.12041

34. Lin WP, Lin JH, Chen XW, Wu CY, Zhang LQ, Huang ZD, et al. Interleukin-10 Promoter Polymorphisms Associated with Susceptibility to Lumbar Disc Degeneration in a Chinese Cohort. Research Support, Non-U.S. Gov't. *Genetics & Molecular Research*. 2011;10(3):1719-27.

35. Makino H, Kawaguchi Y, Seki S, Nakano M, Yasuda T, Suzuki K, et al. Lumbar Disc Degeneration Progression in Young Women in Their 20's: A Prospective Ten-Year Follow Up. *Journal of Orthopaedic Science*. Jul 2017;22(4):635-640. doi:https://dx.doi.org/10.1016/j.jos.2017.03.015

36. Marchiori DM, Mclean I, Firth R, Tatum R. A Comparison of Radiographic Signs of Degeneration to Corresponding Mri Signal Intensities in the Lumbar Spine. Comparative Study. *Journal of Manipulative & Physiological Therapeutics*. May 1994;17(4):238-45.

37. Matsui H, Kanamori M, Ishihara H, Yudoh K, Naruse Y, Tsuji H. Familial Predisposition for Lumbar Degenerative Disc Disease. A Case-Control Study. *Spine*. May 01 1998;23(9):1029-34.

38. Mok FP, Samartzis D, Karppinen J, Fong DY, Luk KD, Cheung KM. Modic Changes of the Lumbar Spine: Prevalence, Risk Factors, and Association with Disc Degeneration and Low Back Pain in a Large-Scale Population-Based Cohort. Research Support, Non-U.S. Gov't. *Spine Journal: Official Journal of the North American Spine Society*. Jan 01 2016;16(1):32-41. doi:https://dx.doi.org/10.1016/j.spinee.2015.09.060

39. Mok FP, Samartzis D, Karppinen J, Luk KD, Fong DY, Cheung KM. Issls Prize Winner: Prevalence, Determinants, and Association of Schmorl Nodes of the Lumbar Spine with Disc Degeneration: A Population-Based Study of 2449 Individuals. Research Support, Non-U.S. Gov't. *Spine*. Oct 01 2010;35(21):1944-52. doi:https://dx.doi.org/10.1097/BRS.0b013e3181d534f3

40. Samartzis D, Karppinen J, Chan D, Luk KD, Cheung KM. The Association of Lumbar Intervertebral Disc Degeneration on Magnetic Resonance Imaging with Body Mass Index in Overweight and Obese Adults: A Population-Based Study. Research Support, Non-U.S. Gov't. *Arthritis & Rheumatism*. May 2012;64(5):1488-96. doi:https://dx.doi.org/10.1002/art.33462

41. Samartzis D, Karppinen J, Mok F, Fong DY, Luk KD, Cheung KM. A Population-Based Study of Juvenile Disc Degeneration and Its Association with Overweight and Obesity, Low Back Pain, and Diminished Functional Status. Research Support, Non-U.S. Gov't. *Journal of Bone & Joint Surgery - American Volume*. Apr 06 2011;93(7):662-70. doi:https://dx.doi.org/10.2106/JBJS.I.01568

42. Samartzis D, Mok FPS, Karppinen J, Fong DYT, Luk KDK, Cheung KMC. Classification of Schmorl's Nodes of the Lumbar Spine and Association with Disc Degeneration: A Large-Scale Population-Based Mri Study. *Osteoarthritis & Cartilage*. 10 2016;24(10):1753-1760. doi:https://dx.doi.org/10.1016/j.joca.2016.04.020

43. Schistad EI, Bjorland S, Roe C, Gjerstad J, Vetti N, Myhre K, et al. Five-Year Development of Lumbar Disc Degeneration-a Prospective Study. *Skeletal Radiology*. Jun 2019;48(6):871-879. doi:https://dx.doi.org/10.1007/s00256-018-3062-x

44. Song YQ, Ho DW, Karppinen J, Kao PY, Fan BJ, Luk KD, et al. Association between Promoter -1607 Polymorphism of Mmp1 and Lumbar Disc Disease in Southern Chinese. Research Support, Non-U.S. Gov't. *BMC Medical Genetics*. Apr 28 2008;9:38. doi:https://dx.doi.org/10.1186/1471-2350-9-38

45. Sun ZM, Ling M, Huo Y, Chang Y, Li Y, Qin H, et al. Caspase 9 Gene Polymorphism and Susceptibility to Lumbar Disc Disease in the Han Population in Northern China. *Connective Tissue Research*. June 2011;52(3):198-202. doi:https://dx.doi.org/10.3109/03008207.2010.510914

46. Sun ZM, Miao L, Zhang YG, Ming L. Association between the -1562 C/T Polymorphism of Matrix Metalloproteinase-9 Gene and Lumbar Disc Disease in the Young Adult Population in North China. Research Support, Non-U.S. Gov't. *Connective Tissue Research*. 2009;50(3):181-5. doi:https://dx.doi.org/10.1080/03008200802585630

47. Watanabe T, Otani K, Sekiguchi M, Konno SI. Relationship between Lumbar Disc Degeneration on Mri and Low Back Pain: A Cross-Sectional Community Study. *Fukushima Journal of Medical Science*. Aug 18 2022;68(2):97-107. doi:https://dx.doi.org/10.5387/fms.2022-17

48. Zehra U, Cheung JPY, Bow C, Lu W, Samartzis D. Multidimensional Vertebral Endplate Defects Are Associated with Disc Degeneration, Modic Changes, Facet Joint Abnormalities, and Pain. *Journal of Orthopaedic Research*. 05 2019;37(5):1080-1089. doi:https://dx.doi.org/10.1002/jor.24195

49. Kanamori M, Nobukiyo M, Suzuki K, Yasuda T, Hori T. Clinical Validity of a New T2-Weighted Mri-Based Grading System for Lumbar Disc Degeneration. *International Medical Journal*. August 2013;20(4):466-469.

50. Masahiko K, Masanori N, Kayo S, Taketoshi Y, Takeshi H. Clinical Validity of a New T2-Weighted Mri-Based Grading System for Lumbar Disc Degeneration. *International Medical Journal*. 2013;20(4):466-469.

51. Nagy SA, Juhasz I, Komaromy H, Pozsar K, Zsigmond I, Perlaki G, et al. A Statistical Model for Intervertebral Disc Degeneration: Determination of the Optimal T2 Cut-Off Values. Research Support, Non-U.S. Gov't. *Clinical Neuroradiology*. Dec 2014;24(4):355-63. doi:https://dx.doi.org/10.1007/s00062-013-0266-2

52. Solovieva S, Kouhia S, Leino-Arjas P, Ala-Kokko L, Luoma K, Raininko R, et al. Interleukin 1 Polymorphisms and Intervertebral Disc Degeneration. Comparative Study Research Support, Non-U.S. Gov't Research Support, U.S. Gov't, P.H.S. *Epidemiology*. Sep 2004;15(5):626-33.

53. Solovieva S, Lohiniva J, Leino-Arjas P, Raininko R, Luoma K, Ala-Kokko L, et al. Intervertebral Disc Degeneration in Relation to the Col9a3 and the Il-1ss Gene Polymorphisms. Research Support, N.I.H., Extramural Research Support, Non-U.S. Gov't. *European Spine Journal*. May 2006;15(5):613-9.

54. Solovieva S, Lohiniva J, Leino-Arjas P, Raininko R, Luoma K, Ala-Kokko L, et al. Col9a3 Gene Polymorphism and Obesity in Intervertebral Disc Degeneration of the Lumbar Spine: Evidence of Gene-Environment Interaction. *Spine (03622436)*. 2002;27(23):2691-2696.

55. Solovieva S, Noponen N, Mannikko M, Leino-Arjas P, Luoma K, Raininko R, et al. Association between the Aggrecan Gene Variable Number of Tandem Repeats Polymorphism and Intervertebral Disc Degeneration. Research Support, N.I.H., Extramural Research Support, Non-U.S. Gov't. *Spine*. Jul 15 2007;32(16):1700-5.

56. Jensen RK, Jensen TS, Kjaer P, Kent P. Can Pathoanatomical Pathways of Degeneration in Lumbar Motion Segments Be Identified by Clustering Mri Findings. *BMC Musculoskeletal Disorders*. 2013;14 (no pagination)198. doi:https://dx.doi.org/10.1186/1471-2474-14-198

57. Jensen RK, Kent P, Hancock M. Do Mri Findings Identify Patients with Chronic Low Back Pain and Modic Changes Who Respond Best to Rest or Exercise: A Subgroup Analysis of a Randomised Controlled Trial. *Chiropractic & manual therapies*. 2015;23:26. doi:https://dx.doi.org/10.1186/s12998-015-0071-x

58. Jensen RK, Kent P, Jensen TS, Kjaer P. The Association between Subgroups of Mri Findings Identified with Latent Class Analysis and Low Back Pain in 40-Year-Old Danes. Observational Study Research Support, Non-U.S. Gov't. *BMC Musculoskeletal Disorders*. 02 20 2018;19(1):62. doi:https://dx.doi.org/10.1186/s12891-018-1978-x

59. Jensen RK, Kjaer P, Jensen TS, Albert H, Kent P. Degenerative Pathways of Lumbar Motion Segments--a Comparison in Two Samples of Patients with Persistent Low Back Pain. Research Support, Non-U.S. Gov't. *PLoS ONE [Electronic Resource]*. 2016;11(1):e0146998. doi:https://dx.doi.org/10.1371/journal.pone.0146998

60. Jensen TS, Bendix T, Sorensen JS, Manniche C, Korsholm L, Kjaer P. Characteristics and Natural Course of Vertebral Endplate Signal (Modic) Changes in the Danish General Population. Research Support, Non-U.S. Gov't. *BMC Musculoskeletal Disorders*. Jul 03 2009;10:81. doi:https://dx.doi.org/10.1186/1471-2474-10-81

61. Luoma K, Vehmas T, Gronblad M, Kerttula L, Kaapa E. Mri Follow-up of Subchondral Signal Abnormalities in a Selected Group of Chronic Low Back Pain Patients. Research Support, Non-U.S. Gov't. *European Spine Journal*. Oct 2008;17(10):1300-8. doi:https://dx.doi.org/10.1007/s00586-008-0716-8

62. Luoma K, Vehmas T, Kerttula L, Gronblad M, Rinne E. Chronic Low Back Pain in Relation to Modic Changes, Bony Endplate Lesions, and Disc Degeneration in a Prospective Mri Study. *European Spine Journal*. 09 2016;25(9):2873-81. doi:https://dx.doi.org/10.1007/s00586-016-4715-x

63. Borenstein DG, O'mara JW, Jr., Boden SD, Lauerman WC, Jacobson A, Platenberg C, et al. The Value of Magnetic Resonance Imaging of the Lumbar Spine to Predict Low-Back Pain in Asymptomatic Subjects : A Seven-Year Follow-up Study. *Journal of Bone & Joint Surgery - American Volume*. Sep 2001;83(9):1306-11.

64. Buttermann GR, Mullin WJ. Pain and Disability Correlated with Disc Degeneration Via Magnetic Resonance Imaging in Scoliosis Patients. *European Spine Journal*. Feb 2008;17(2):240-9.

65. Karppinen J, Paakko E, Paassilta P, Lohiniva J, Kurunlahti M, Tervonen O, et al. Radiologic Phenotypes in Lumbar Mr Imaging for a Gene Defect in the Col9a3 Gene of Type Ix Collagen. *Radiology*. 01 Apr 2003;227(1):143-148. doi:https://dx.doi.org/10.1148/radiol.2271011821

66. Lakadamyali H, Tarhan NC, Ergun T, Cakir B, Agildere AM. Stir Sequence for Depiction of Degenerative Changes in Posterior Stabilizing Elements in Patients with Lower Back Pain. *AJR. American Journal of Roentgenology*. Oct 2008;191(4):973-9. doi:https://dx.doi.org/10.2214/AJR.07.2829

67. Sabnis AB, Chamoli U, Diwan AD. Is L5-S1 Motion Segment Different from the Rest? A Radiographic Kinematic Assessment of 72 Patients with Chronic Low Back Pain. Research Support, Non-U.S. Gov't. *European Spine Journal*. 05 2018;27(5):1127-1135. doi:https://dx.doi.org/10.1007/s00586-017-5400-4

68. Throckmorton TW, Hilibrand AS, Mencio GA, Hodge A, Spengler DM. The Impact of Adjacent Level Disc Degeneration on Health Status Outcomes Following Lumbar Fusion. *Spine*. Nov 15 2003;28(22):2546-50.

69. Leboeuf-Yde C, Kjaer P, Bendix T, Manniche C. Self-Reported Hard Physical Work Combined with Heavy Smoking or Overweight May Result in So-Called Modic Changes. Comparative Study Research Support, Non-U.S. Gov't. *BMC Musculoskeletal Disorders*. Jan 14 2008;9:5. doi:https://dx.doi.org/10.1186/1471-2474-9-5

70. Irurhe NK, Adekola OO, Quadri AR, Menkiti ID, Udenze IC, Awolola NA. The Magnetic Resonance Imaging Scan Findings in Adult Nigerians with Low Back Pain. *World Journal of Medical Sciences*. 2012;7(4):204-209. doi:https://dx.doi.org/10.5829/idosi.wjms.2012.7.4.6427

71. Kiil RM, Mistegaard CE, Loft AG, Zejden A, Hendricks O, Jurik AG. Differences in Topographical Location of Sacroiliac Joint Mri Lesions in Patients with Early Axial Spondyloarthritis and Mechanical Back Pain. *Arthritis Research & Therapy*. 03 24 2022;24(1):75. doi:https://dx.doi.org/10.1186/s13075-022-02760-7

72. Kim J, Park HJ, Kim MS, Kim JN, Choi YJ, Rho MH, et al. Wedging of Vertebral Bodies at the Thoracolumbar Spine in Healthy Individuals on Whole Body Mri Screening: Correlation with Disc Degeneration and Disc Herniation. *Acta Radiologica*. Jul 2022;63(7):958-963. doi:https://dx.doi.org/10.1177/02841851211024005

73. Michopoulou SK, Costaridou L, Panagiotopoulos E, Speller R, Panayiotakis G, Todd-Pokropek A. Atlas-Based Segmentation of Degenerated Lumbar Intervertebral Discs from Mr Images of the Spine. Research Support, Non-U.S. Gov't. *IEEE Transactions on Biomedical Engineering*. Sep 2009;56(9):2225-31. doi:https://dx.doi.org/10.1109/TBME.2009.2019765

74. Witwit W, Thoreson O, Sward Aminoff A, Todd C, Jonasson P, Laxdal G, et al. Young Football Players Have Significantly More Spinal Changes on Mri Compared to Non-Athletes. Review. *Translational Sports Medicine*. 01 Jul 2020;3(4):288-295. doi:https://dx.doi.org/10.1002/tsm2.144

75. Witwit WA, Hebelka H, Sward Aminoff A, Abrahamson J, Todd C, Baranto A. No Significant Change in Mri Abnormalities or Back Pain Prevalence in the Thoraco-Lumbar Spine of Young Elite Skiers over a 2-Year Follow-Up. *Open Access Journal of Sports Medicine*. 2022;13:69-76. doi:https://dx.doi.org/10.2147/OAJSM.S366548

76. Witwit WA, Kovac P, Sward A, Agnvall C, Todd C, Thoreson O, et al. Disc Degeneration on Mri Is More Prevalent in Young Elite Skiers Compared to Controls. Observational Study. *Knee Surgery, Sports Traumatology, Arthroscopy*. Jan 2018;26(1):325-332. doi:https://dx.doi.org/10.1007/s00167-017-4545-3

77. Battie MC, Videman T, Gibbons LE, Fisher LD, Manninen H, Gill K. Determinants of Lumbar Disc Degeneration: A Study Relating Lifetime Exposures and Magnetic Resonance Imaging Findings in Identical Twins. *Spine*. 1995;20(24):2601-2612.

78. Videman T, Leppavuori J, Kaprio J, Battie MC, Gibbons LE, Peltonen L, et al. Intragenic Polymorphisms of the Vitamin D Receptor Gene Associated with Intervertebral Disc Degeneration. Research Support, Non-U.S. Gov't Research Support, U.S. Gov't, P.H.S. Twin Study. *Spine*. Dec 01 1998;23(23):2477-85.

79. Horton WC, Daftari TK. Which Disc as Visualized by Magnetic Resonance Imaging Is Actually a Source of Pain? A Correlation between Magnetic Resonance Imaging and Discography. *Spine (Phila Pa 1976)*. Jun 1992;17(6 Suppl):S164-71. doi:10.1097/00007632-199206001-00018

80. Boden SD, Riew KD, Yamaguchi K, Branch TP, Schellinger D, Wiesel SW. Orientation of the Lumbar Facet Joints: Association with Degenerative Disc Disease. *Journal of Bone & Joint Surgery - American Volume*. Mar 1996;78(3):403-11.

81. Videman T, Saarela J, Kaprio J, Nakki A, Levalahti E, Gill K, et al. Associations of 25 Structural, Degradative, and Inflammatory Candidate Genes with Lumbar Disc Desiccation, Bulging, and Height Narrowing. Research Support, N.I.H., Extramural Research Support, Non-U.S. Gov't. *Arthritis & Rheumatism*. Feb 2009;60(2):470-81. doi:https://dx.doi.org/10.1002/art.24268

82. Videman T, Gibbons LE, Battie MC. Age- and Pathology-Specific Measures of Disc Degeneration. Comparative Study Research Support, N.I.H., Extramural Research Support, Non-U.S. Gov't Twin Study. *Spine*. Dec 01 2008;33(25):2781-8. doi:https://dx.doi.org/10.1097/BRS.0b013e31817e1d11

83. Battie MC, Videman T, Levalahti E, Gill K, Kaprio J. Genetic and Environmental Effects on Disc Degeneration by Phenotype and Spinal Level: A Multivariate Twin Study. Comparative Study Research Support, N.I.H., Extramural Research Support, Non-U.S. Gov't Twin Study. *Spine*. Dec 01 2008;33(25):2801-8. doi:https://dx.doi.org/10.1097/BRS.0b013e31818043b7

84. Deng C, Xia W. Effect of Tai Chi Chuan on Degeneration of Lumbar Vertebrae and Lumbar Discs in Middle-Aged and Aged People: A Cross-Sectional Study Based on Magnetic Resonance Images. *Journal of International Medical Research*. Feb 2018;46(2):578-585. doi:https://dx.doi.org/10.1177/0300060517734115

85. Desigan S, Hall-Craggs MA, Ho CP, Eliahoo J, Porter JB. Degenerative Disc Disease as a Cause of Back Pain in the Thalassaemic Population: A Case-Control Study Using Mri and Plain Radiographs. Controlled Clinical Trial. *Skeletal Radiology*. Feb 2006;35(2):95-102.

86. Videman T, Battie MC, Gibbons LE, Manninen H, Gill K, Fisher LD, et al. Lifetime Exercise and Disk Degeneration: An Mri Study of Monozygotic Twins. Research Support, Non-U.S. Gov't Research Support, U.S. Gov't, P.H.S. Twin Study. *Medicine & Science in Sports & Exercise*. Oct 1997;29(10):1350-6.

87. Aaen J, Austevoll IM, Hellum C, Storheim K, Myklebust TA, Banitalebi H, et al. Clinical and Mri Findings in Lumbar Spinal Stenosis: Baseline Data from the Nordsten Study. Clinical Trial

Research Support, Non-U.S. Gov't. *European Spine Journal*. 06 2022;31(6):1391-1398. doi:https://dx.doi.org/10.1007/s00586-021-07051-4

88. Aaen J, Banitalebi H, Austevoll IM, Hellum C, Storheim K, Myklebust TA, et al. The Association between Preoperative Mri Findings and Clinical Improvement in Patients Included in the Nordsten Spinal Stenosis Trial. Clinical Trial

Research Support, Non-U.S. Gov't. *European Spine Journal*. 10 2022;31(10):2777-2785. doi:https://dx.doi.org/10.1007/s00586-022-07317-5

89. Aavikko A, Lohman M, Ristolainen L, Kautiainen H, Osterman K, Schlenzka D, et al. Issls Prize in Clinical Science 2022: Accelerated Disc Degeneration after Pubertal Growth Spurt Differentiates Adults with Low Back Pain from Their Asymptomatic Peers. Observational Study

Research Support, Non-U.S. Gov't. *European Spine Journal*. 05 2022;31(5):1080-1087. doi:https://dx.doi.org/10.1007/s00586-022-07184-0

90. Abdalkader M, Guermazi A, Engebretsen L, Roemer FW, Jarraya M, Hayashi D, et al. Mri-Detected Spinal Disc Degenerative Changes in Athletes Participating in the Rio De Janeiro 2016 Summer Olympics Games. *BMC Musculoskeletal Disorders*. Jan 20 2020;21(1):45. doi:https://dx.doi.org/10.1186/s12891-020-3057-3

91. Abdollah V, Parent EC, Su A, Wachowicz K, Battié MC. The Effects of Axial Loading on the Morphometric and T2 Characteristics of Lumbar Discs in Relation to Disc Degeneration. *Clinical Biomechanics*. 2021;83:N.PAG-N.PAG. doi:10.1016/j.clinbiomech.2021.105291

92. Abou Khadrah RS, Dawoud MF, Abo-Elsafa AA, Elkilany AM. Advanced Trends in Magnetic Resonance Imaging in Assessment of Lumbar Intervertebral Degenerative Disk Disease. *Egyptian Journal of Radiology and Nuclear Medicine*. 01 Dec 2019;50(1) (no pagination)43. doi:https://dx.doi.org/10.1186/s43055-019-0042-7

93. Ahn TJ, Lee SH, Choi G, Ahn Y, Liu WC, Kim HJ, et al. Effect of Intervertebral Disk Degeneration on Spinal Stenosis During Magnetic Resonance Imaging with Axial Loading. Research Support, Non-U.S. Gov't. *Neurologia Medico-Chirurgica*. Jun 2009;49(6):242-7; discussion 247.

94. Akazawa T, Kotani T, Sakuma T, Minami S, Orita S, Fujimoto K, et al. Spinal Fusion on Adolescent Idiopathic Scoliosis Patients with the Level of L4 or Lower Can Increase Lumbar Disc Degeneration with Sagittal Imbalance 35 Years after Surgery. *Spine Surgery & Related Research*. 2017;1(2):72-77. doi:https://dx.doi.org/10.22603/ssrr.1.2016-0017

95. Akazawa T, Kotani T, Sakuma T, Minami S, Orita S, Inage K, et al. Modic Changes and Disc Degeneration of Nonfused Segments 27 to 45 Years after Harrington Instrumentation for Adolescent Idiopathic Scoliosis: Comparison to Healthy Controls. *Spine (03622436)*. 2017;42(15):N.PAG-N.PAG. doi:10.1097/BRS.0000000000002362

96. Akazawa T, Umehara T, Iinuma M, Asano K, Kuroya S, Torii Y, et al. Spinal Alignments of Residual Lumbar Curve Affect Disc Degeneration after Spinal Fusion in Patients with Adolescent Idiopathic Scoliosis: Follow-up after 5 or More Years. *Spine Surgery and Related Research*. 2020;4(1):50-56. doi:https://dx.doi.org/10.22603/ssrr.2019-0048

97. Akazawa T, Watanabe K, Matsumoto M, Tsuji T, Kawakami N, Kotani T, et al. Modic Changes and Disc Degeneration in Adolescent Idiopathic Scoliosis Patients Who Reach Middle Age without Surgery: Can Residual Deformity Cause Lumbar Spine Degeneration? *Journal of Orthopaedic Science*. Nov 2018;23(6):884-888. doi:https://dx.doi.org/10.1016/j.jos.2018.07.002

98. Alicioglu B, Sut N. Synovial Cysts of the Lumbar Facet Joints: A Retrospective Magnetic Resonance Imaging Study Investigating Their Relation with Degenerative Spondylolisthesis. *Prague Medical Report*. 2009;110(4):301-9.

99. Alkhasawneh MH, Al-Mnayyis A, Bagain Y. Spinal Degeneration and Degenerative Disc Disease Correlation Identified with Magnetic Resonance Imaging. *Biomedical and Pharmacology Journal*. March 2021;14(1):491-496. doi:https://dx.doi.org/10.13005/bpj/2149

100. Alserafy AM, Badran M, El-Nasr AA, El-Fiki A, Halaby W. Pre-Existing Adjacent Level Degeneration Effect on Decision Making in Single Level Lumbar Spondylolisthesis. *Journal of Cardiovascular Disease Research*. 2021;12(3):1004-1010. doi:https://dx.doi.org/10.31838/jcdr.2021.12.03.129

101. Alyas F, Turner M, Connell D. Mri Findings in the Lumbar Spines of Asymptomatic, Adolescent, Elite Tennis Players. Research Support, Non-U.S. Gov't. *British Journal of Sports Medicine*. Nov 2007;41(11):836-41; discussion 841.

102. Apaydin M, Kalayci OT, Varer M, Sezgin G, Uluc E. Lumbosacral Transitional Vertebra in the Young Men Population with Low Back Pain: Anatomical Considerations and Degenerations. Conference Abstract. *Neuroradiology*. September 2015;1):S137. doi:https://dx.doi.org/10.1007/s00234-015-1557-x

103. Arana E, Royuela A, Kovacs FM, Estremera A, Sarasibar H, Amengual G, et al. Lumbar Spine: Agreement in the Interpretation of 1.5-T Mr Images by Using the Nordic Modic Consensus Group Classification Form. *Radiology*. March 2010;254(3):809-817. doi:https://dx.doi.org/10.1148/radiol.09090706

104. Arslan E, Demirci I, Kilincaslan MO, Hacifazlioglu C, Demir T, Demirkale I. Identification of Intervertebral Disc Regeneration with Magnetic Resonance Imaging after a Long-Term Follow-up in Patients Treated with Percutaneous Diode Laser Nucleoplasty: A Retrospective Clinical and Radiological Analysis of 14 Patients. *European Spine Journal*. May 2014;23(5):1044-51. doi:https://dx.doi.org/10.1007/s00586-014-3194-1

105. Atalay A, Turhan N, Atalay B. Deconditioning in Chronic Low Back Pain: Might There Be a Relationship between Fitness and Magnetic Resonance Imaging Findings? Comparative Study. *Rheumatology International*. Jan 2012;32(1):21-5. doi:https://dx.doi.org/10.1007/s00296-010-1544-9

106. Auerbach JD, Johannessen W, Borthakur A, Wheaton AJ, Dolinskas CA, Balderston RA, et al. In Vivo Quantification of Human Lumbar Disc Degeneration Using T(1rho)-Weighted Magnetic Resonance Imaging. Research Support, N.I.H., Extramural. *European Spine Journal*. Aug 2006;15 Suppl 3:S338-44.

107. Baioni A, Silvestre M, Greggi T, Vommaro F, Lolli F, Scarale A, et al. Does Hybrid Fixation Prevent Junctional Disease after Posterior Fusion for Degenerative Lumbar Disorders? A Minimum 5-Year Follow-up Study. *European Spine Journal*. 2015;24:855-864. doi:10.1007/s00586-015-4269-3

108. Banno T, Hasegawa T, Yamato Y, Yoshida G, Arima H, Oe S, et al. Disc Degeneration Could Be Recovered after Chemonucleolysis with Condoliase.-1 Year Clinical Outcome of Condoliase Therapy. *Journal of Orthopaedic Science*. Jul 2022;27(4):767-773. doi:https://dx.doi.org/10.1016/j.jos.2021.05.005

109. Bao H, Zhu F, Liu Z, Zhu Z, He S, Ding Y, et al. Coronal Curvature and Spinal Imbalance in Degenerative Lumbar Scoliosis: Disc Degeneration Is Associated. Research Support, Non-U.S. Gov't. *Spine*. Nov 15 2014;39(24):E1441-7. doi:https://dx.doi.org/10.1097/BRS.0000000000000603

110. Basaran R, Senol M, Ozkanli S, Efendioglu M, Kaner T. Correlation of Matrix Metalloproteinase (Mmp)-1, -2, -3, and -9 Expressions with Demographic and Radiological Features in Primary Lumbar Intervertebral Disc Disease. *Journal of Clinical Neuroscience*. Jul 2017;41:46-49. doi:https://dx.doi.org/10.1016/j.jocn.2017.03.001

111. Basques BA, Espinoza Orias AA, Shifflett GD, Fice MP, Andersson GB, An HS, et al. The Kinematics and Spondylosis of the Lumbar Spine Vary Depending on the Levels of Motion Segments in Individuals with Low Back Pain. *Spine*. Jul 01 2017;42(13):E767-E774. doi:https://dx.doi.org/10.1097/BRS.0000000000001967

112. Bazan PL, Borri AE, Medina M. Correlation between the Modic I Sign and Images of Vertebral Instability. Correlacao entre sinal modic i e imagem de instabilidade vertebral, correlacion entre signo de modic i e imagen de inestabilidad vertebral. *Coluna/ Columna*. 2021;20(4):264-267. doi:https://dx.doi.org/10.1590/S1808-185120212004250503

113. Belykh E, Kalinin AA, Patel AA, Miller EJ, Bohl MA, Stepanov IA, et al. Apparent Diffusion Coefficient Maps in the Assessment of Surgical Patients with Lumbar Spine Degeneration. *PLoS ONE [Electronic Resource]*. 2017;12(8):e0183697. doi:https://dx.doi.org/10.1371/journal.pone.0183697

114. Benedikter C, Abrar DB, Konieczny M, Schleich C, Bittersohl B. Patterns of Intervertebral Disk Alteration in Asymptomatic Elite Rowers: A T2* Mri Mapping Study. *Orthopaedic Journal of Sports Medicine*. 2022;10(4)doi:https://dx.doi.org/10.1177/23259671221088572

115. Berg AJ, Ahmadje U, Jayanna HH, Tregouet P, Sanville P, Kapoor V. The Prevalence of Lumbar Disc Degeneration in Symptomatic Younger Patients: A Study of Mri Scans. *Journal of Clinical Orthopaedics & Trauma*. Sep-Oct 2020;11(5):932-936. doi:https://dx.doi.org/10.1016/j.jcot.2020.07.021

116. Bernstein P, Hentschel S, Platzek I, Huhne S, Ettrich U, Hartmann A, et al. Thoracal Flat Back Is a Risk Factor for Lumbar Disc Degeneration after Scoliosis Surgery. Comparative Study

Research Support, Non-U.S. Gov't. *Spine Journal: Official Journal of the North American Spine Society*. Jun 01 2014;14(6):925-32. doi:https://dx.doi.org/10.1016/j.spinee.2013.07.426

117. Bezuglov E, Lazarev A, Petrov A, Brodskaia A, Lyubushkina A, Kubacheva K, et al. Asymptomatic Degenerative Changes in the Lumbar Spine among Professional Soccer Players. *Spine*. Jan 15 2021;46(2):122-128. doi:https://dx.doi.org/10.1097/BRS.0000000000003726

118. Bo R, Yang QG, Duan W, Liu JR, Zhang YS. Correlation between Vertebral Endplate Shape and Intervertebral Disc Degeneration. *Chinese Journal of Tissue Engineering Research*. 2012;16(24):4413-4416. doi:https://dx.doi.org/10.3969/j.issn.1673-8225.2012.24.009

119. Borthakur A, Maurer PM, Fenty M, Wang C, Berger R, Yoder J, et al. T<Inf>1rho</Inf> Magnetic Resonance Imaging and Discography Pressure as Novel Biomarkers for Disc Degeneration and Low Back Pain. *Spine*. 01 Dec 2011;36(25):2190-2196. doi:https://dx.doi.org/10.1097/BRS.0b013e31820287bf

120. Byval'tsev VA, Stepanov IA, Kalinin AA, Belykh EG. Diffusion-Weighted Magnetic Resonance Imaging in the Diagnosis of Intervertebral Disc Degeneration in the Lumbosacral Spine. *Vestnik Rentgenologii i Radiologii*. Nov-Dec 2016;97(6):357-64.

121. Canbay S, Turhan N, Bozkurt M, Arda K, Caglar S. Correlation of Matrix Metalloproteinase-3 Expression with Patient Age, Magnetic Resonance Imaging and Histopathological Grade in Lumbar Disc Degeneration. *Turkish Neurosurgery*. 2013;23(4):427-33. doi:https://dx.doi.org/10.5137/1019-5149.JTN.7459-12.0

122. Cao Y, Guo QW, Wan YD. Significant Association between the T2 Values of Vertebral Cartilage Endplates and Pfirrmann Grading. *Orthopaedic Audio-Synopsis Continuing Medical Education [Sound Recording]*. Aug 2020;12(4):1164-1172. doi:https://dx.doi.org/10.1111/os.12727

123. Castro-Mateos I, Hua R, Pozo J, Lazary A, Frangi A, Pozo JM, et al. Intervertebral Disc Classification by Its Degree of Degeneration from T2-Weighted Magnetic Resonance Images. *European Spine Journal*. 2016;25(9):2721-2727. doi:10.1007/s00586-016-4654-6

124. Chen L, Hu X, Zhang J, Battie MC, Lin X, Wang Y. Modic Changes in the Lumbar Spine Are Common Aging-Related Degenerative Findings That Parallel with Disk Degeneration. Research Support, Non-U.S. Gov't. *Clinical Spine Surgery : A Spine Publication*. 08 2018;31(7):312-317. doi:https://dx.doi.org/10.1097/BSD.0000000000000662

125. Chen N, Lang N, Yuan H. Ultrashort Echo Time Mri on Cartilaginous Endplates in Lumbar Spine. [Chinese]. Mri. *Chinese Journal of Medical Imaging Technology*. 20 Jun 2019;35(6):899-903. doi:https://dx.doi.org/10.13929/j.1003-3289.201812037

126. Chen R, Liang X, Huang T, Zhong W, Luo X. Effects of Type 1 Diabetes Mellitus on Lumbar Disc Degeneration: A Retrospective Study of 118 Patients. *Journal of Orthopaedic Surgery*. Jul 25 2020;15(1):280. doi:https://dx.doi.org/10.1186/s13018-020-01784-6

127. Chen SQ, Li QP, Huang YY, Guo AN, Zhang RF, Ye PP, et al. Different Spinal Subtypes with Varying Characteristics of Lumbar Disc Degeneration at Specific Level with Age: A Study Based on an Asymptomatic Population. Observational Study. *Journal of Orthopaedic Surgery*. Jan 03 2020;15(1):3. doi:https://dx.doi.org/10.1186/s13018-019-1537-7

128. Cheng Z, Li Y, Li M, Huang J, Liang Y, Lu S, et al. Correlation between Posterior Paraspinal Muscle Atrophy and Lumbar Intervertebral Disc Degeneration in Patients with Chronic Low Back Pain. *International Orthopaedics*. March 2023;47(3):793-801. doi:https://dx.doi.org/10.1007/s00264-022-05621-9

129. Chiu CK, Tan CS, Chung WH, Mohamad SM, Kwan MK, Chan CYW. Mid-Long-Term Outcome and Degeneration of the Remaining Unfused Lumbar Intervertebral Disc in Adolescent Idiopathic Scoliosis Patients Who Had Posterior Spinal Fusion Surgery. *European Spine Journal*. 07 2021;30(7):1978-1987. doi:https://dx.doi.org/10.1007/s00586-021-06874-5

130. Collinet A, Charles YP, Ntilikina Y, Tuzin N, Steib JP. Analysis of Intervertebral Discs Adjacent to Thoracolumbar A3 Fractures Treated by Percutaneous Instrumentation and Kyphoplasty. Review. *Orthopaedics & traumatology, surgery & research*. Oct 2020;106(6):1221-1226. doi:https://dx.doi.org/10.1016/j.otsr.2020.05.006

131. Corniola MV, Stienen MN, Joswig H, Smoll NR, Schaller K, Hildebrandt G, et al. Correlation of Pain, Functional Impairment, and Health-Related Quality of Life with Radiological Grading Scales of Lumbar Degenerative Disc Disease. *Acta Neurochirurgica*. 01 Mar 2016;158(3):499-505. doi:https://dx.doi.org/10.1007/s00701-015-2700-5

132. Crewe H, Elliott B, Couanis G, Campbell A, Alderson J. The Lumbar Spine of the Young Cricket Fast Bowler: An Mri Study. *Journal of Science and Medicine in Sport*. May 2012;15(3):190-194. doi:https://dx.doi.org/10.1016/j.jsams.2011.11.251

133. Cubuk R, Kozakcioglu M, Tasali N, Atalay A, Celik L. Lumbar Disc and Facet Degeneration: Correlation with Age and Facet Orientation. *Trakya Universitesi Tip Fakultesi Dergisi*. 2009;26(1):36-42.

134. Cuellar VG, Cuellar JM, Vaccaro AR, Carragee EJ, Scuderi GJ. Accelerated Degeneration after Failed Cervical and Lumbar Nucleoplasty. *Journal of Spinal Disorders & Techniques*. Dec 2010;23(8):521-4. doi:https://dx.doi.org/10.1097/BSD.0b013e3181cc90dd

135. Cui J, Zhou R, Tian N, Sui X, Huang M, Hao D, et al. Correlation between Lower Lumbar Multifidus Muscles Fatty Atrophy and Corresponding Level Degenerative Diseases in Patients with Low Back Pain Using Mri. *Chinese Journal of Academic Radiology*. March 2021;4(1):63-70. doi:https://dx.doi.org/10.1007/s42058-021-00054-6

136. Cui JH, Kim YC, Lee K, Park GT, Kim KT, Kim SM. Relationship between Facet Joint Tropism and Degeneration of Facet Joints and Intervertebral Discs Based on a Histological Study. *Journal of Orthopaedics*. Mar-Apr 2019;16(2):123-127. doi:https://dx.doi.org/10.1016/j.jor.2018.12.008

137. Cui YZ, Yang XH, Liu PF, Wang B, Chen WJ. Preliminary Study on Diagnosis of Lumbar Disc Degeneration with Magnetic Resonance T1p, T2 Mapping and Dwi Quantitative Detection Technologies. Research Support, Non-U.S. Gov't. *European Review for Medical & Pharmacological Sciences*. 08 2016;20(16):3344-50.

138. Davies BM, Atkinson RA, Ludwinski F, Freemont AJ, Hoyland JA, Gnanalingham KK. Qualitative Grading of Disc Degeneration by Magnetic Resonance in the Lumbar and Cervical Spine: Lack of Correlation with Histology in Surgical Cases. *British Journal of Neurosurgery*. Aug 2016;30(4):414-21. doi:https://dx.doi.org/10.3109/02688697.2016.1161174

139. Deguchi T, Hashizume H, Nakajima M, Teraguchi M, Akune T, Yamada H, et al. A Population-Based Study Identifies an Association of Thbs2 with Intervertebral Disc Degeneration. *Osteoarthritis and Cartilage*. October 2019;27(10):1501-1507. doi:https://dx.doi.org/10.1016/j.joca.2019.06.001

140. Dehnokhalaji M, Golbakhsh MR, Siavashi B, Talebian P, Javidmehr S, Bozorgmanesh M. Evaluation of the Degenerative Changes of the Distal Intervertebral Discs after Internal Fixation Surgery in Adolescent Idiopathic Scoliosis. *Asian Spine Journal*. Dec 2018;12(6):1060-1068. doi:https://dx.doi.org/10.31616/asj.2018.12.6.1060

141. Ding Y, Chen JY, Yang JC, Li RY, Yin YJ, Chen JT, et al. Disc Degeneration Contributes to the Denser Bone in the Subendplate but Not in the Vertebral Body in Patients with Lumbar Spinal Stenosis or Disc Herniation. Research Support, Non-U.S. Gov't. *Spine Journal: Official Journal of the North American Spine Society*. 01 2023;23(1):64-71. doi:https://dx.doi.org/10.1016/j.spinee.2022.09.010

142. Dogan A, Dogan K, Tasolar S. Magnetic Resonance Imaging Evaluation of the Effects of Cigarette and Maras Powder (Smokeless Tobacco) on Lumbar Disc Degeneration. *Clinical Neurology & Neurosurgery*. Nov 2019;186:105500. doi:https://dx.doi.org/10.1016/j.clineuro.2019.105500

143. Doktor K, Hartvigsen J, Hancock M, Christensen HW, Fredberg U, Boyle E, et al. Reliability of Reporting Differences in Degenerative Mri Findings of the Lumbar Spine from the Supine to the Upright Position. *Skeletal Radiology*. November 2022;51(11):2141-2154. doi:https://dx.doi.org/10.1007/s00256-022-04060-2

144. Doktor K, Jensen TS, Christensen HW, Fredberg U, Kindt M, Boyle E, et al. Degenerative Findings in Lumbar Spine Mri: An Inter-Rater Reliability Study Involving Three Raters. Research Support, Non-U.S. Gov't. *Chiropractic & manual therapies*. 02 11 2020;28(1):8. doi:https://dx.doi.org/10.1186/s12998-020-0297-0

145. Doyle AJ, Merrilees M. Synovial Cysts of the Lumbar Facet Joints in a Symptomatic Population: Prevalence on Magnetic Resonance Imaging. *Spine*. Apr 15 2004;29(8):874-8.

146. Duran S, Cavusoglu M, Gunaydin E, Sakman B. Ligamentum Flavum Hypertrophy in Elderly Patients with Low Back Pain: A Mri Study. Bel agrisi bulunan yasli hastalarda ligamentum flavum hipertrofisi: MRG calismasi. *Turk Geriatri Dergisi*. 2016;19(2):107-112.

147. Dybvik V, Hermansen E, Banitalebi H, Myklebust TA, Indrekvam K. Is Repeated Preoperative Magnetic Resonance Imaging Necessary before Planned Decompressive Surgery for Lumbar Spinal Stenosis? *International Journal of Spine Surgery*. Mar 24 2023;24:24. doi:https://dx.doi.org/10.14444/8469

148. Eksi MS, Ozcan-Eksi EE, Akkas A, Orhun O, Arslan HN, Zarbizada M, et al. Intradiscal Vacuum Phenomenon and Spinal Degeneration: A Cross-Sectional Analysis of 219 Subjects. *Current Medical Research & Opinion*. Feb 2022;38(2):255-263. doi:https://dx.doi.org/10.1080/03007995.2021.1994379

149. Eksi MS, Ozcan-Eksi EE, Orhun O, Huet SE, Turgut VU, Pamir MN. Association between Facet Joint Orientation/Tropism and Lumbar Intervertebral Disc Degeneration. *British Journal of Neurosurgery*. Dec 26 2020:1-8. doi:https://dx.doi.org/10.1080/02688697.2020.1864289

150. Eksi MS, Ozcan-Eksi EE, Orhun O, Turgut VU, Pamir MN. Proposal for a New Scoring System for Spinal Degeneration: Mo-Fi-Disc. *Clinical Neurology & Neurosurgery*. 11 2020;198:106120. doi:https://dx.doi.org/10.1016/j.clineuro.2020.106120

151. Eksi MS, Ozcan-Eksi EE, Ozmen BB, Turgut VU, Huet SE, Dinc T, et al. Lumbar Intervertebral Disc Degeneration, End-Plates and Paraspinal Muscle Changes in Children and Adolescents with Low-Back Pain. *Journal of Pediatric Orthopaedics Part B*. 01 Jan 2022;31(1):93-102. doi:https://dx.doi.org/10.1097/BPB.0000000000000833

152. Eksi MS, Turgut VU, Berikol G, Ozmen BB, Huet SE, Dinc T, et al. Schmorl's Nodes Could Be Associated with Intervertebral Disc Degeneration at Upper Lumbar Levels and End-Plate Disease at Lower Lumbar Level in Patients with Low Back Pain. *Journal of Clinical Neuroscience*. Jun 2022;100:66-74. doi:https://dx.doi.org/10.1016/j.jocn.2022.04.004

153. Elfadle AA, Zarad CA, Elmaaty AaA, El-Nagaa BFA, Soliman AY. Correlation between Lumbar Spinal Canal Magnetic Resonance Imaging Grading Systems and Parameters in Lumbar Spinal Canal Compromise. *Egyptian Journal of Neurology, Psychiatry and Neurosurgery*. December 2022;58(1) (no pagination)104. doi:https://dx.doi.org/10.1186/s41983-022-00543-0

154. Enercan M, Kahraman S, Yilar S, Cobanoglu M, Gokcen BH, Karadereler S, et al. Does It Make a Difference to Stop Fusion at L3 Versus L4 in Terms of Disc and Facet Joint Degeneration: An Mri Study with Minimum 5 Years Follow-Up. *Spine Deformity*. May 2016;4(3):237-244. doi:https://dx.doi.org/10.1016/j.jspd.2015.12.001

155. Enoki S, Kuramochi R, Nakajyuku S, Mitsuyama H. The Prevalence of Spondylolysis and Intervertebral Disc Degeneration in Male Pole Vaulters. *Journal of Back & Musculoskeletal Rehabilitation*. 2022;35(1):147-151. doi:https://dx.doi.org/10.3233/BMR-200296

156. Ergun T, Lakadamyali H, Sahin MS. The Relation between Sagittal Morphology of the Lumbosacral Spine and the Degree of Lumbar Intervertebral Disc Degeneration. *Acta Orthopaedica et Traumatologica Turcica*. 2010;44(4):293-9. doi:https://dx.doi.org/10.3944/AOTT.2010.2375

157. Farshad-Amacker N, Hughes A, Herzog R, Seifert B, Farshad M, Farshad-Amacker NA, et al. The Intervertebral Disc, the Endplates and the Vertebral Bone Marrow as a Unit in the Process of Degeneration. *European Radiology*. 2017;27(6):2507-2520. doi:10.1007/s00330-016-4584-z

158. Farshad-Amacker N, Hughes AP, Aichmair A, Herzog RJ, Farshad M. Determinants of Evolution of Endplate and Disc Degeneration in the Lumbar Spine-a Multifactorial Perspective. Conference Abstract. *Swiss Medical Weekly*. 11 Jun 2014;204):30S.

159. Farshad-Amacker NA, Herzog RJ, Hughes AP, Aichmair A, Farshad M. Associations between Lumbosacral Transitional Anatomy Types and Degeneration at the Transitional and Adjacent Segments. *Spine Journal: Official Journal of the North American Spine Society*. Jun 01 2015;15(6):1210-6. doi:https://dx.doi.org/10.1016/j.spinee.2013.10.029

160. Farshad-Amacker NA, Hughes AP, Aichmair A, Herzog RJ, Farshad M. Determinants of Evolution of Endplate and Disc Degeneration in the Lumbar Spine: A Multifactorial Perspective. Research Support, Non-U.S. Gov't. *European Spine Journal*. Sep 2014;23(9):1863-8. doi:https://dx.doi.org/10.1007/s00586-014-3382-z

161. Farshad-Amacker NA, Hughes AP, Aichmair A, Herzog RJ, Farshad M. Is an Annular Tear a Predictor for Accelerated Disc Degeneration? Research Support, Non-U.S. Gov't. *European Spine Journal*. Sep 2014;23(9):1825-9. doi:https://dx.doi.org/10.1007/s00586-014-3260-8

162. Filippi CG, Duncan CT, Watts R, Nickerson JP, Gonyea JV, Hipko SG, et al. In Vivo Quantification of T1rho in Lumbar Spine Disk Spaces at 3 T Using Parallel Transmission Mri. Research Support, U.S. Gov't, Non-P.H.S. *AJR. American Journal of Roentgenology*. Jul 2013;201(1):W110-6. doi:https://dx.doi.org/10.2214/AJR.12.9523

163. Foizer GA, Paiva VC, Nascimento RDD, Gorios C, Cliquet Junior A, Miranda JB. Is There Any Association between the Severity of Disc Degeneration and Low Back Pain? *Revista Brasileira de Ortopedia*. Apr 2022;57(2):334-340. doi:https://dx.doi.org/10.1055/s-0041-1735831

164. Folkvardsen S, Magnussen E, Karppinen J, Auvinen J, Larsen R, Wong C, et al. Does Elite Swimming Accelerate Lumbar Intervertebral Disc Degeneration and Increase Low Back Pain? A Cross-Sectional Comparison. *European Spine Journal*. 2016;25(9):2849-2855. doi:10.1007/s00586-016-4642-x

165. Frenken M, Nebelung S, Schleich C, Muller-Lutz A, Radke KL, Kamp B, et al. Non-Specific Low Back Pain and Lumbar Radiculopathy: Comparison of Morphologic and Compositional Mri as Assessed by Gagcest Imaging at 3t. *Diagnostics*. Feb 26 2021;11(3):26. doi:https://dx.doi.org/10.3390/diagnostics11030402

166. Fu CL, Zhang B, Liu Y, Dai M, Zhou X, Fu XX. Mri Comparison of Lumbar Facet Joint Degeneration and Intervertebral Disc Degeneration in Patients with Low Back Pain. [Chinese]. *Chinese Journal of Tissue Engineering Research*. 2015;19(46):7401-7405. doi:https://dx.doi.org/10.3969/j.issn.2095-4344.2015.46.005

167. Fu L, France A, Xie Y, Fang K, Gan Y, Zhang P. Functional and Radiological Outcomes of Semi-Rigid Dynamic Lumbar Stabilization Adjacent to Single-Level Fusion after 2 Years. Observational Study Research Support, Non-U.S. Gov't. *Archives of Orthopaedic & Trauma Surgery*. May 2014;134(5):605-10. doi:https://dx.doi.org/10.1007/s00402-014-1961-4

168. Fujita N, Ishihara S, Michikawa T, Azuma K, Suzuki S, Tsuji O, et al. Potential Association of Metabolic and Musculoskeletal Disorders with Lumbar Intervertebral Disc Degeneration: Cross-Sectional Study Using Medical Checkup Data. *Journal of Orthopaedic Science*. May 2020;25(3):384-388. doi:https://dx.doi.org/10.1016/j.jos.2019.05.011

169. Galbusera F, Niemeyer F, Tao Y, Cina A, Sconfienza LM, Kienle A, et al. Issls Prize in Bioengineering Science 2021: In Vivo Sagittal Motion of the Lumbar Spine in Low Back Pain Patients-a Radiological Big Data Study. Research Support, Non-U.S. Gov't. *European Spine Journal*. 05 2021;30(5):1108-1116. doi:https://dx.doi.org/10.1007/s00586-021-06729-z

170. Galley J, Balague F. Revisiting Radiographic L5-S1 Parallelism Using Mri T1 Mapping. *Journal of the Belgian Society of Radiology*. Sep 27 2018;102(1):59. doi:https://dx.doi.org/10.5334/jbsr.1501

171. Gao F, Liu S, Zhang X, Wang X, Zhang J. Automated Grading of Lumbar Disc Degeneration Using a Push-Pull Regularization Network Based on Mri. *Journal of Magnetic Resonance Imaging*. 03 2021;53(3):799-806. doi:https://dx.doi.org/10.1002/jmri.27400

172. Gao J, Zhao W, Zhang X, Nong L, Zhou D, Lv Z, et al. Mri Analysis of the Isobar Ttl Internal Fixation System for the Dynamic Fixation of Intervertebral Discs: A Comparison with Rigid Internal Fixation. Comparative Study. *Journal of Orthopaedic Surgery*. Jun 04 2014;9:43. doi:https://dx.doi.org/10.1186/1749-799X-9-43

173. Gao X, Wang L, Zhang J, Wang P, Shen Y. Long Fusion Arthrodesis Stopping at L5 for Adult Scoliosis: Fate of L5-S1 Disk and Risk Factors for Subsequent Disk Degeneration. *Clinical Spine Surgery : A Spine Publication*. 04 2018;31(3):E171-E177. doi:https://dx.doi.org/10.1097/BSD.0000000000000624

174. Gautschi OP, Stienen MN, Joswig H, Smoll NR, Schaller K, Corniola MV. The Usefulness of Radiological Grading Scales to Predict Pain Intensity, Functional Impairment, and Health-Related Quality of Life after Surgery for Lumbar Degenerative Disc Disease. *Acta Neurochirurgica*. 02 2017;159(2):271-279. doi:https://dx.doi.org/10.1007/s00701-016-3030-y

175. Golan JD, Martens F, Griebel J, Lopresti DC, Hess MG, Ahrens M. Long-Term Outcomes Following Lumbar Nucleus Replacement. *International Journal of Spine Surgery*. Dec 2021;15(6):1096-1102. doi:https://dx.doi.org/10.14444/8196

176. Grannum S, Torrie PA, Miller A, Harding IJ. Risk Factors for the Development of a Mobile Degenerative Spondylolisthesis at L4-L5. *Spine Deformity*. Jan 2015;3(1):98-104. doi:https://dx.doi.org/10.1016/j.jspd.2014.06.012

177. Green DW, Lawhorne TW, 3rd, Widmann RF, Kepler CK, Ahern C, Mintz DN, et al. Long-Term Magnetic Resonance Imaging Follow-up Demonstrates Minimal Transitional Level Lumbar Disc Degeneration after Posterior Spine Fusion for Adolescent Idiopathic Scoliosis. *Spine (03622436)*. 2011;36(23):1948-1954. doi:10.1097/BRS.0b013e3181ff1ea9

178. Griffith JF, Wang YX, Antonio GE, Choi KC, Yu A, Ahuja AT, et al. Modified Pfirrmann Grading System for Lumbar Intervertebral Disc Degeneration. Clinical Trial Validation Study. *Spine*. Nov 15 2007;32(24):E708-12.

179. Grob A, Loibl M, Jamaludin A, Winklhofer S, Fairbank JCT, Fekete T, et al. External Validation of the Deep Learning System "Spinenet" for Grading Radiological Features of Degeneration on Mris of the Lumbar Spine. Research Support, Non-U.S. Gov't. *European Spine Journal*. 08 2022;31(8):2137-2148. doi:https://dx.doi.org/10.1007/s00586-022-07311-x

180. Guo Y, Li C, Shen B, Chen X, Hu T, Wu D. Is Intervertebral Disc Degeneration Associated with Reduction in Serum Ferritin? *European Spine Journal*. 11 2022;31(11):2950-2959. doi:https://dx.doi.org/10.1007/s00586-022-07361-1

181. Guo Y, Li C, Shen B, Zhu Z, Chen X, Hu T, et al. Is There Any Relationship between Plasma Il-6 and Tnf-Alpha Levels and Lumbar Disc Degeneration? A Retrospective Single-Center Study. *Disease Markers*. 2022;2022:6842130. doi:https://dx.doi.org/10.1155/2022/6842130

182. Guo Y, Zhao H, Lu J, Xu H, Hu T, Wu D. Preoperative Lymphocyte to Monocyte Ratio as a Predictive Biomarker for Disease Severity and Spinal Fusion Failure in Lumbar Degenerative Diseases Patients Undergoing Lumbar Fusion. *Journal of Pain Research*. 2022;15:2879-2891. doi:https://dx.doi.org/10.2147/JPR.S379453

183. Hancock M, Maher C, Macaskill P, Latimer J, Kos W, Pik J, et al. Mri Findings Are More Common in Selected Patients with Acute Low Back Pain Than Controls? *European Spine Journal*. 2012;21(2):240-246. doi:10.1007/s00586-011-1955-7

184. Hancock MJ, Maher CM, Petocz P, Lin C-WC, Steffens D, Luque-Suarez A, et al. Risk Factors for a Recurrence of Low Back Pain. *Spine Journal*. 2015;15(11):2360-2368. doi:10.1016/j.spinee.2015.07.007

185. Haneder S, Apprich SR, Schmitt B, Michaely HJ, Schoenberg SO, Friedrich KM, et al. Assessment of Glycosaminoglycan Content in Intervertebral Discs Using Chemical Exchange Saturation Transfer at 3.0 Tesla: Preliminary Results in Patients with Low-Back Pain. Research Support, Non-U.S. Gov't. *European Radiology*. Mar 2013;23(3):861-8. doi:https://dx.doi.org/10.1007/s00330-012-2660-6

186. Haneder S, Ong MM, Budjan JM, Schmidt R, Konstandin S, Morelli JN, et al. 23na-Magnetic Resonance Imaging of the Human Lumbar Vertebral Discs: In Vivo Measurements at 3.0 T in Healthy Volunteers and Patients with Low Back Pain. *Spine Journal: Official Journal of the North American Spine Society*. Jul 01 2014;14(7):1343-50. doi:https://dx.doi.org/10.1016/j.spinee.2014.01.031

187. Hangai M, Kaneoka K, Hinotsu S, Shimizu K, Okubo Y, Miyakawa S, et al. Lumbar Intervertebral Disk Degeneration in Athletes. Research Support, Non-U.S. Gov't. *American Journal of Sports Medicine*. Jan 2009;37(1):149-55. doi:https://dx.doi.org/10.1177/0363546508323252

188. Hangai M, Kaneoka K, Kuno S, Hinotsu S, Sakane M, Mamizuka N, et al. Factors Associated with Lumbar Intervertebral Disc Degeneration in the Elderly. Research Support, Non-U.S. Gov't. *Spine Journal: Official Journal of the North American Spine Society*. Sep-Oct 2008;8(5):732-40.

189. Hanhivaara J, Maatta JH, Karppinen J, Niinimaki J, Nevalainen MT. The Association of Lumbosacral Transitional Vertebrae with Low Back Pain and Lumbar Degenerative Findings in Mri: A Large Cohort Study. *Spine*. Jan 15 2022;47(2):153-162. doi:https://dx.doi.org/10.1097/BRS.0000000000004244

190. Hanimoglu H, Cevik S, Yilmaz H, Kaplan A, Calis F, Katar S, et al. Effects of Modic Type 1 Changes in the Vertebrae on Low Back Pain. *World Neurosurgery*. Jan 2019;121:e426-e432. doi:https://dx.doi.org/10.1016/j.wneu.2018.09.132

191. Hansen BB, Ciochon UM, Trampedach CR, Christensen AF, Rasti Z, Boesen M. Grading Lumbar Disc Degeneration: A Comparison between Low- and High-Field Mri. Comparative Study. *Acta Radiologica*. Dec 2019;60(12):1636-1642. doi:https://dx.doi.org/10.1177/0284185119842472

192. Hasegawa K, Kitahara K, Hara T, Takano K, Shimoda H, Homma T. Evaluation of Lumbar Segmental Instability in Degenerative Diseases by Using a New Intraoperative Measurement System. *Journal of Neurosurgery Spine*. Mar 2008;8(3):255-62. doi:https://dx.doi.org/10.3171/SPI/2008/8/3/255

193. Hayashi T, Daubs MD, Suzuki A, Scott TP, Phan KH, Ruangchainikom M, et al. Motion Characteristics and Related Factors of Modic Changes in the Lumbar Spine. *Journal of Neurosurgery Spine*. May 2015;22(5):511-7. doi:https://dx.doi.org/10.3171/2014.10.SPINE14496

194. He X, Liang A, Gao W, Peng Y, Zhang L, Liang G, et al. The Relationship between Concave Angle of Vertebral Endplate and Lumbar Intervertebral Disc Degeneration. Research Support, Non-U.S. Gov't. *Spine*. Aug 01 2012;37(17):E1068-73. doi:https://dx.doi.org/10.1097/BRS.0b013e31825640eb

195. Hebelka H, Brisby H, Hansson T. Comparison between Pain at Discography and Morphological Disc Changes at Axial Loaded Mri in Patients with Low Back Pain. Comparative Study Research Support, Non-U.S. Gov't. *European Spine Journal*. Oct 2014;23(10):2075-82. doi:https://dx.doi.org/10.1007/s00586-014-3408-6

196. Hebelka H, Gunterberg V, Lagerstrand K, Brisby H. Clinical Outcome and Mri Appearance in a Group of Chronic Low Back Pain Patients More Than 10 Years after Discography Evaluation and Consideration for Surgery. *BMC Musculoskeletal Disorders*. Feb 22 2023;24(1):138. doi:https://dx.doi.org/10.1186/s12891-023-06242-y

197. Hey HWD, Ng NL, Loh KYS, Tan YH, Tan KA, Moorthy V, et al. Sagittal Radiographic Parameters of the Spine in Three Physiological Postures Characterized Using a Slot Scanner and Their Potential Implications on Spinal Weight-Bearing Properties. *Asian Spine Journal*. Feb 2021;15(1):23-31. doi:https://dx.doi.org/10.31616/asj.2019.0198

198. Hong C, Lee CG, Song H. Characteristics of Lumbar Disc Degeneration and Risk Factors for Collapsed Lumbar Disc in Korean Farmers and Fishers. *Annals of Occupational and Environmental Medicine*. 2021;33(1) (no pagination)e16. doi:https://dx.doi.org/10.35371/aoem.2021.33.e16

199. Hoppe S, Quirbach S, Mamisch TC, Krause FG, Werlen S, Benneker LM. Axial T2 Mapping in Intervertebral Discs: A New Technique for Assessment of Intervertebral Disc Degeneration. Research Support, Non-U.S. Gov't. *European Radiology*. Sep 2012;22(9):2013-9. doi:https://dx.doi.org/10.1007/s00330-012-2448-8

200. Hornung AL, Barajas JN, Rudisill SS, Aboushaala K, Butler A, Park G, et al. Prediction of Lumbar Disc Herniation Resorption in Symptomatic Patients: A Prospective, Multi-Imaging and Clinical Phenotype Study. *Spine Journal: Official Journal of the North American Spine Society*. 02 2023;23(2):247-260. doi:https://dx.doi.org/10.1016/j.spinee.2022.10.003

201. Hsieh CC, Wang JD, Lin RM, Lin CJ, Huang KY. Adjacent Disc and Facet Joint Degeneration in Young Adults with Low-Grade Spondylolytic Spondylolisthesis: A Magnetic Resonance Imaging Study. Research Support, Non-U.S. Gov't. *Journal of the Formosan Medical Association*. Dec 2015;114(12):1211-5. doi:https://dx.doi.org/10.1016/j.jfma.2014.09.004

202. Hu J, Zhang Y, Duan C, Peng X, Hu P, Lu H. Feasibility Study for Evaluating Early Lumbar Facet Joint Degeneration Using Axial T<Inf>1</Inf>Rho, T<Inf>2</Inf>, and T2* Mapping in Cartilage. *Journal of Magnetic Resonance Imaging*. August 2017;46(2):468-475. doi:https://dx.doi.org/10.1002/jmri.25596

203. Hu JK, Morishita Y, Montgomery SR, Hymanson H, Taghavi CE, Do D, et al. Kinematic Evaluation of Association between Disc Bulge Migration, Lumbar Segmental Mobility, and Disc Degeneration in the Lumbar Spine Using Positional Magnetic Resonance Imaging. *Global Spine Journal*. Dec 2011;1(1):43-8. doi:https://dx.doi.org/10.1055/s-0031-1296056

204. Huang Y, Liu J, Guo L, Meng Y, Hao D, Du J. "Temporary" Short Segment Fixation in Treating Adolescent Lumbar Spondylolysis. *World Neurosurgery*. Mar 2019;123:e77-e84. doi:https://dx.doi.org/10.1016/j.wneu.2018.11.046

205. Huang Y, Wang L, Zeng X, Chen J, Zhang Z, Jiang Y, et al. Association of Paraspinal Muscle Csa and Pdff Measurements with Lumbar Intervertebral Disk Degeneration in Patients with Chronic Low Back Pain. Research Support, Non-U.S. Gov't. *Frontiers in Endocrinology*. 2022;13:792819. doi:https://dx.doi.org/10.3389/fendo.2022.792819

206. Ibrahim M, Arockiaraj J, Amritanand R, Venkatesh K, David KS. Recurrent Lumbar Disc Herniation: Results of Revision Surgery and Assessment of Factors That May Affect the Outcome. A Non-Concurrent Prospective Study. *Asian Spine Journal*. Oct 2015;9(5):728-36. doi:https://dx.doi.org/10.4184/asj.2015.9.5.728

207. Identeg F, Lagerstrand K, Hedelin H, Senorski EH, Sansone M, Hebelka H. Low Occurrence of Mri Spinal Changes in Elite Climbing Athletes; a Cross-Sectional Study. *BMC Sports Science, Medicine and Rehabilitation*. Mar 09 2023;15(1):29. doi:https://dx.doi.org/10.1186/s13102-023-00637-z

208. Iguchi T, Nishida K, Ozaki T, Kitagawa A, Tsumura N, Kakutani K, et al. Grade Three Disc Degeneration Is a Critical Stage for Anterior Spondylolisthesis in Lumbar Spine. *European Spine Journal*. Nov 2012;21(11):2134-9. doi:https://dx.doi.org/10.1007/s00586-012-2288-x

209. Iii WS, Orias AaE, Shifflett GD, Lee JYB, Siemionow K, Gandhi S, et al. Image-Based Markers Predict Dynamic Instability in Lumbar Degenerative Spondylolisthesis. *Neurospine*. Mar 2020;17(1):221-227. doi:https://dx.doi.org/10.14245/ns.1938440.220

210. Illeez OG, Ulger FEB, Aktas I. The Effect of Transitional Vertebrae and Spina Bifida Occulta on Disc Herniation, Disc Degeneration, and End-Plate Changes in Pediatric Patients with Low Back Pain. *Acta Orthopaedica Belgica*. Jun 2022;88(2):275-283. doi:https://dx.doi.org/10.52628/88.2.8528

211. Imagama S, Kawakami N, Kanemura T, Matsubara Y, Tsuji T, Ohara T, et al. Radiographic Adjacent Segment Degeneration at Five Years after L4/5 Posterior Lumbar Interbody Fusion with Pedicle Screw Instrumentation: Evaluation by Computed Tomography and Annual Screening with Magnetic Resonance Imaging. In Press. *Journal of Spinal Disorders and Techniques.* 2013;19doi:https://dx.doi.org/10.1097/BSD.0b013e31828aec78

212. Iriondo C, Pedoia V, Majumdar S. Lumbar Intervertebral Disc Characterization through Quantitative Mri Analysis: An Automatic Voxel-Based Relaxometry Approach. Research Support, N.I.H., Extramural. *Magnetic Resonance in Medicine*. 09 2020;84(3):1376-1390. doi:https://dx.doi.org/10.1002/mrm.28210

213. Jain A, Jain S, Barasker SK, Agrawal A. Predictors of Discogenic Pain in Magnetic Resonance Imaging: A Retrospective Study of Provocative Discography Performed by Posterolateral Approach. *The Korean journal of pain*. Oct 01 2021;34(4):447-453. doi:https://dx.doi.org/10.3344/kjp.2021.34.4.447

214. Jakkepally S, Viswanathan VK, Shetty AP, Hajare S, Kanna RM, Rajasekaran S. The Analysis of Progression of Disc Degeneration in Distal Unfused Segments and Evaluation of Long-Term Functional Outcome in Adolescent Idiopathic Scoliosis Patients Undergoing Long-Segment Instrumented Fusion. Research Support, Non-U.S. Gov't. *Spine Deformity*. 03 2022;10(2):343-350. doi:https://dx.doi.org/10.1007/s43390-021-00428-x

215. Jamaludin A, Kadir T, Zisserman A. Spinenet: Automated Classification and Evidence Visualization in Spinal Mris. *Medical Image Analysis*. October 2017;41:63-73. doi:https://dx.doi.org/10.1016/j.media.2017.07.002

216. Jamaludin A, Kadir T, Zisserman A, Mccall I, Williams FMK, Lang H, et al. Age and Disc Degeneration in Low Back Pain: Automated Analysis Enables a Magnetic Resonance Imaging Comparison of Large Cross-Sectional Cohorts of Symptomatic and Asymptomatic Subjects. Preprint. *medRxiv.* 2021;08doi:https://dx.doi.org/10.1101/2021.11.08.21265571

217. Jamaludin A, Kadir T, Zisserman A, Mccall I, Williams FMK, Lang H, et al. Issls Prize in Clinical Science 2023: Comparison of Degenerative Mri Features of the Intervertebral Disc between Those with and without Chronic Low Back Pain. An Exploratory Study of Two Large Female Populations Using Automated Annotation. *European Spine Journal*. Mar 30 2023;30:30. doi:https://dx.doi.org/10.1007/s00586-023-07604-9

218. Jamaludin A, Lootus M, Kadir T, Zisserman A, Urban J, Battie MC, et al. Issls Prize in Bioengineering Science 2017: Automation of Reading of Radiological Features from Magnetic Resonance Images (Mris) of the Lumbar Spine without Human Intervention Is Comparable with an Expert Radiologist. Comparative Study. *European Spine Journal*. 05 2017;26(5):1374-1383. doi:https://dx.doi.org/10.1007/s00586-017-4956-3

219. Janardhana AP, Rajagopal, Rao S, Kamath A. Correlation between Clinical Features and Magnetic Resonance Imaging Findings in Lumbar Disc Prolapse. *Indian Journal of Orthopaedics*. Jul 2010;44(3):263-9. doi:https://dx.doi.org/10.4103/0019-5413.65148

220. Jang SY, Kong MH, Hymanson HJ, Jin TK, Song KY, Wang JC. Radiographic Parameters of Segmental Instability in Lumbar Spine Using Kinetic Mri. *Journal of Korean Neurosurgical Society*. Jan 2009;45(1):24-31. doi:https://dx.doi.org/10.3340/jkns.2009.45.1.24

221. Jang TW, Ahn YS, Byun J, Lee JI, Kim KH, Kim Y, et al. Lumbar Intervertebral Disc Degeneration and Related Factors in Korean Firefighters. Multicenter Study Research Support, Non-U.S. Gov't. *BMJ Open*. 06 28 2016;6(6):e011587. doi:https://dx.doi.org/10.1136/bmjopen-2016-011587

222. Jarman JP, Arpinar VE, Baruah D, Klein AP, Maiman DJ, Muftuler LT. Intervertebral Disc Height Loss Demonstrates the Threshold of Major Pathological Changes During Degeneration. Research Support, Non-U.S. Gov't. *European Spine Journal*. Sep 2015;24(9):1944-50. doi:https://dx.doi.org/10.1007/s00586-014-3564-8

223. Jeng CM, Cheng TC, Kung CH, Hsu HC. Yoga and Disc Degenerative Disease in Cervical and Lumbar Spine: An Mr Imaging-Based Case Control Study. Case Reports Comparative Study. *European Spine Journal*. Mar 2011;20(3):408-13. doi:https://dx.doi.org/10.1007/s00586-010-1547-y

224. Jha SC, Takata Y, Abe M, Yamashita K, Tezuka F, Sakai T, et al. High Intensity Zone in Lumbar Spine and Its Correlation with Disc Degeneration. *Journal of Medical Investigation*. 2017;64(1.2):39-42. doi:https://dx.doi.org/10.2152/jmi.64.39

225. Ji Y, Hong W, Liu M, Liang Y, Deng Y, Ma L. Intervertebral Disc Degeneration Associated with Vertebral Marrow Fat, Assessed Using Quantitative Magnetic Resonance Imaging. *Skeletal Radiology*. Nov 2020;49(11):1753-1763. doi:https://dx.doi.org/10.1007/s00256-020-03419-7

226. Jiang Y, Yu L, Luo X, Lin Y, He B, Wu B, et al. Quantitative Synthetic Mri for Evaluation of the Lumbar Intervertebral Disk Degeneration in Patients with Chronic Low Back Pain. Evaluation Study. *European Journal of Radiology*. Mar 2020;124:108858. doi:https://dx.doi.org/10.1016/j.ejrad.2020.108858

227. Jung M, Rospleszcz S, Loffler MT, Walter SS, Maurer E, Jungmann PM, et al. Association of Lumbar Vertebral Bone Marrow and Paraspinal Muscle Fat Composition with Intervertebral Disc Degeneration: 3t Quantitative Mri Findings from the Population-Based Kora Study. *European Radiology*. Mar 2023;33(3):1501-1512. doi:https://dx.doi.org/10.1007/s00330-022-09140-4

228. Kamei N, Nakamae T, Nakanishi K, Tamura T, Tsuchikawa Y, Morisako T, et al. Evaluation of Intervertebral Disc Degeneration Using T2 Signal Ratio on Magnetic Resonance Imaging. *European Journal of Radiology*. Jul 2022;152:110358. doi:https://dx.doi.org/10.1016/j.ejrad.2022.110358

229. Kaneoka K, Shimizu K, Hangai M, Okuwaki T, Mamizuka N, Sakane M, et al. Lumbar Intervertebral Disk Degeneration in Elite Competitive Swimmers: A Case Control Study. *American Journal of Sports Medicine*. Aug 2007;35(8):1341-5.

230. Kanna RM, Hajare S, Thippeswamy PB, Shetty AP, Rajasekaran S. Advanced Disc Degeneration, Bi-Planar Instability and Pathways of Peri-Discal Gas Suffusion Contribute to Pathogenesis of Intradiscal Vacuum Phenomenon. Research Support, Non-U.S. Gov't. *European Spine Journal*. 03 2022;31(3):755-763. doi:https://dx.doi.org/10.1007/s00586-022-07122-0

231. Kanna RM, Shetty AP, Rajasekaran S. Patterns of Lumbar Disc Degeneration Are Different in Degenerative Disc Disease and Disc Prolapse Magnetic Resonance Imaging Analysis of 224 Patients. Comparative Study Observational Study Research Support, Non-U.S. Gov't. *Spine Journal: Official Journal of the North American Spine Society*. Feb 01 2014;14(2):300-7. doi:https://dx.doi.org/10.1016/j.spinee.2013.10.042

232. Keorochana G, Taghavi CE, Lee KB, Yoo JH, Liao JC, Fei Z, et al. Effect of Sagittal Alignment on Kinematic Changes and Degree of Disc Degeneration in the Lumbar Spine: An Analysis Using Positional Mri. *Spine*. May 15 2011;36(11):893-8. doi:https://dx.doi.org/10.1097/BRS.0b013e3181f4d212

233. Khodair SA, Ghieda UE, Eltomey MA. Relationship of Lumbosacral Spine Morphometrics and Lumber Disc Degenerative Disease in Young Adults Using Magnetic Resonance Imaging. *Egyptian Journal of Radiology and Nuclear Medicine*. 2014;45(2):461-466. doi:https://dx.doi.org/10.1016/j.ejrnm.2014.02.005

234. Kilic G, Senol S, Baspinar S, Kilic E, Ozgocmen S. Degenerative Changes of Lumbar Spine and Their Clinical Implications in Patients with Axial Spondyloarthritis. *Clinical Rheumatology*. Jan 2023;42(1):111-116. doi:https://dx.doi.org/10.1007/s10067-022-06321-w

235. Kim HJ, Suh BG, Lee DB, Lee GW, Kim DW, Kang KT, et al. The Influence of Pain Sensitivity on the Symptom Severity in Patients with Lumbar Spinal Stenosis. *Pain Physician*. Mar-Apr 2013;16(2):135-44.

236. Kim HJ, Suh BG, Lee DB, Park JY, Kang KT, Chang BS, et al. Gender Difference of Symptom Severity in Lumbar Spinal Stenosis: Role of Pain Sensitivity. *Pain Physician*. Nov-Dec 2013;16(6):E715-23.

237. Kim JY, Ryu DS, Paik HK, Ahn SS, Kang MS, Kim KH, et al. Paraspinal Muscle, Facet Joint, and Disc Problems: Risk Factors for Adjacent Segment Degeneration after Lumbar Fusion. *Spine Journal: Official Journal of the North American Spine Society*. 07 2016;16(7):867-75. doi:https://dx.doi.org/10.1016/j.spinee.2016.03.010

238. Kim SY, Lee IS, Kim BR, Lim JH, Lee J, Koh SE, et al. Magnetic Resonance Findings of Acute Severe Lower Back Pain. *Annals of Rehabilitation Medicine*. Feb 2012;36(1):47-54. doi:https://dx.doi.org/10.5535/arm.2012.36.1.47

239. Kleinstuck F, Dvorak J, Mannion AF. Are "Structural Abnormalities" on Magnetic Resonance Imaging a Contraindication to the Successful Conservative Treatment of Chronic Nonspecific Low Back Pain? *Spine*. Sep 01 2006;31(19):2250-7.

240. Kobayashi K, Sato K, Ando T. Factors Associated with Disc Degeneration Based on Pfirrmann Criteria after Condoliase Treatment for Lumbar Disc Herniation. *Journal of Orthopaedic Science*. Aug 24 2022;24:24. doi:https://dx.doi.org/10.1016/j.jos.2022.08.001

241. Kobayashi K, Sato K, Ando T, Ando K. Mri Characteristics of Disc Degeneration after Condoliase Injection in Young Patients: A Consecutive Case Series. *Journal of Orthopaedic Science*. Mar 04 2023;04:04. doi:https://dx.doi.org/10.1016/j.jos.2023.02.013

242. Kojima T, Kubo S, Tajima N, Mitsuhashi R, Nozaki S, Chosa E. Lumbar Intervertebral Disc Degeneration in Professional Surfers. Lumbale Bandscheibendegeneration bei professionellen Surfern. *Sports Orthopaedics and Traumatology*. September 2018;34(3):261-264. doi:https://dx.doi.org/10.1016/j.orthtr.2018.06.006

243. Kong MH, Morishita Y, He W, Miyazaki M, Zhang H, Wu G, et al. Lumbar Segmental Mobility According to the Grade of the Disc, the Facet Joint, the Muscle, and the Ligament Pathology by Using Kinetic Magnetic Resonance Imaging. *Spine*. Nov 01 2009;34(23):2537-44. doi:https://dx.doi.org/10.1097/BRS.0b013e3181b353ea

244. Kovacs FM, Royuela A, Jensen TS, Estremera A, Amengual G, Muriel A, et al. Agreement in the Interpretation of Magnetic Resonance Images of the Lumbar Spine. *Acta Radiologica*. Jun 2009;50(5):497-506. doi:https://dx.doi.org/10.1080/02841850902838074

245. Kraft CN, Pennekamp PH, Becker U, Young M, Diedrich O, Luring C, et al. Magnetic Resonance Imaging Findings of the Lumbar Spine in Elite Horseback Riders: Correlations with Back Pain, Body Mass Index, Trunk/Leg-Length Coefficient, and Riding Discipline. *American Journal of Sports Medicine*. Nov 2009;37(11):2205-13. doi:https://dx.doi.org/10.1177/0363546509336927

246. Krug R, Joseph GB, Han M, Fields A, Cheung J, Mundada M, et al. Associations between Vertebral Body Fat Fraction and Intervertebral Disc Biochemical Composition as Assessed by Quantitative Mri. Research Support, N.I.H., Extramural. *Journal of Magnetic Resonance Imaging*. 10 2019;50(4):1219-1226. doi:https://dx.doi.org/10.1002/jmri.26675

247. Kuisma M, Karppinen J, Haapea M, Lammentausta E, Niinimaki J, Tervonen O. Modic Changes in Vertebral Endplates: A Comparison of Mr Imaging and Multislice Ct. Comparative Study Research Support, Non-U.S. Gov't. *Skeletal Radiology*. Feb 2009;38(2):141-7. doi:https://dx.doi.org/10.1007/s00256-008-0590-9

248. Kuisma M, Karppinen J, Haapea M, Niinimaki J, Ojala R, Heliovaara M, et al. Are the Determinants of Vertebral Endplate Changes and Severe Disc Degeneration in the Lumbar Spine the Same? A Magnetic Resonance Imaging Study in Middle-Aged Male Workers. Comparative Study Research Support, Non-U.S. Gov't. *BMC Musculoskeletal Disorders*. Apr 16 2008;9:51. doi:https://dx.doi.org/10.1186/1471-2474-9-51

249. Kuo C-H, Huang W-C, Wu J-C, Tu T-H, Fay L-Y, Wu C-L, et al. Radiological Adjacent-Segment Degeneration in L4-5 Spondylolisthesis: Comparison between Dynamic Stabilization and Minimally Invasive Transforaminal Lumbar Interbody Fusion. *Journal of Neurosurgery: Spine*. 2018:1-9. doi:10.3171/2018.1.SPINE17993

250. Lagerback T, Kastrati G, Moller H, Jensen K, Skorpil M, Gerdhem P. Mri Characteristics at a Mean of Thirteen Years after Lumbar Disc Herniation Surgery in Adolescents: A Case-Control Study. *JB & JS Open Access*. Oct-Dec 2021;6(4):Oct-Dec. doi:https://dx.doi.org/10.2106/JBJS.OA.21.00081

251. Lao L, Daubs MD, Scott TP, Lord EL, Cohen JR, Yin R, et al. Effect of Disc Degeneration on Lumbar Segmental Mobility Analyzed by Kinetic Magnetic Resonance Imaging. *Spine*. Mar 01 2015;40(5):316-22. doi:https://dx.doi.org/10.1097/BRS.0000000000000738

252. Lao L, Daubs MD, Takahashi S, Lord EL, Cohen JR, Zhong G, et al. Kinetic Magnetic Resonance Imaging Analysis of Lumbar Segmental Motion at Levels Adjacent to Disc Herniation. *European Spine Journal*. Jan 2016;25(1):222-229. doi:https://dx.doi.org/10.1007/s00586-015-3977-z

253. Latif R, Imran S, Ahmad I, Ilyas MS, Aziz A, Zehra U. Vertebral Endplate Changes Correlate with Presence of Cartilaginous Endplate in the Herniated Disc Tissue: Factor Predicting Failure of Conservative Treatment. *Asian Spine Journal*. Apr 2022;16(2):212-220. doi:https://dx.doi.org/10.31616/asj.2021.0106

254. Latz D, Frenken M, Schiffner E, Knautz M, Quante WA, Windolf J, et al. Assessment of Glycosaminoglycan Content in Intervertebral Discs of Patients with Leg Length Discrepancy: A Pilot Study. *Journal of Orthopaedics*. 2019;16(5):363-367. doi:10.1016/j.jor.2019.03.014

255. Lee CS, Ha JK, Kim DG, Hwang CJ, Lee DH, Cho JH. The Clinical Importance of Lumbosacral Transitional Vertebra in Patients with Adolescent Idiopathic Scoliosis. *Spine*. Sep 01 2015;40(17):E964-70. doi:https://dx.doi.org/10.1097/BRS.0000000000000945

256. Lee J, Kim J, Shin JS, Lee YJ, Kim MR, Jeong SY, et al. Long-Term Course to Lumbar Disc Resorption Patients and Predictive Factors Associated with Disc Resorption. *Evidence-based Complementary and Alternative Medicine*. 2017;2017 (no pagination)2147408. doi:https://dx.doi.org/10.1155/2017/2147408

257. Lee JW, Choi SW, Park SH, Lee GY, Kang HS, Lee JW, et al. Mr-Based Outcome Predictors of Lumbar Transforaminal Epidural Steroid Injection for Lumbar Radiculopathy Caused by Herniated Intervertebral Disc. *European Radiology*. 2013;23(1):205-211. doi:10.1007/s00330-012-2566-3

258. Lee K, Shin JS, Lee J, Lee YJ, Kim MR, Seong I, et al. Lumbar Intervertebral Disc Space Height in Disc Herniation and Degeneration Patients Aged 20 to 25. *International Journal of Clinical and Experimental Medicine*. 30 Apr 2017;10(4):6828-6836.

259. Lee SE, Jahng TA, Kim HJ. Clinical Experiences of Non-Fusion Dynamic Stabilization Surgery for Adjacent Segmental Pathology after Lumbar Fusion. *International Journal of Spine Surgery*. 2016;10:8. doi:https://dx.doi.org/10.14444/3008

260. Lee SM, Lee GW. The Impact of Generalized Joint Laxity on the Clinical and Radiological Outcomes of Single-Level Posterior Lumbar Interbody Fusion. Evaluation Study. *Spine Journal: Official Journal of the North American Spine Society*. May 01 2015;15(5):809-16. doi:https://dx.doi.org/10.1016/j.spinee.2014.12.013

261. Li X, Xie Y, Lu R, Zhang Y, Li Q, Kober T, et al. Q-Dixon and Grappatini T2 Mapping Parameters: A Whole Spinal Assessment of the Relationship between Osteoporosis and Intervertebral Disc Degeneration. Research Support, Non-U.S. Gov't. *Journal of Magnetic Resonance Imaging*. 05 2022;55(5):1536-1546. doi:https://dx.doi.org/10.1002/jmri.27959

262. Li Y, Lord E, Cohen Y, Ruangchainikom M, Wang B, Lv G, et al. Effects of Sagittal Endplate Shape on Lumbar Segmental Mobility as Evaluated by Kinetic Magnetic Resonance Imaging. *Spine*. Aug 01 2014;39(17):E1035-41. doi:https://dx.doi.org/10.1097/BRS.0000000000000419

263. Liang J, Dong Y, Zhao H. Risk Factors for Predicting Symptomatic Adjacent Segment Degeneration Requiring Surgery in Patients after Posterior Lumbar Fusion. Research Support, Non-U.S. Gov't. *Journal of Orthopaedic Surgery*. Oct 12 2014;9:97. doi:https://dx.doi.org/10.1186/s13018-014-0097-0

264. Liang X, Xie R, Hou B, Li Y, Xiong Y, Yin C, et al. Feasibility Study for Evaluating Lumbar Intervertebral Disc Degeneration Using Histogram Analysis of T2 Values. Research Support, Non-U.S. Gov't. *European Spine Journal*. 10 2020;29(10):2600-2608. doi:https://dx.doi.org/10.1007/s00586-020-06476-7

265. Liawrungrueang W, Kim P, Kotheeranurak V, Jitpakdee K, Sarasombath P. Automatic Detection, Classification, and Grading of Lumbar Intervertebral Disc Degeneration Using an Artificial Neural Network Model. *Diagnostics*. Feb 10 2023;13(4):10. doi:https://dx.doi.org/10.3390/diagnostics13040663

266. Lippross S, Girmond P, Luders KA, Austein F, Braunschweig L, Luders S, et al. Smaller Intervertebral Disc Volume and More Disc Degeneration after Spinal Distraction in Scoliotic Children. *Journal of Clinical Medicine*. May 2021;10(10) (no pagination)2124. doi:https://dx.doi.org/10.3390/jcm10102124

267. Liu C, Liang G, Deng Z, Tan J, Zheng Q, Lyu FJ. The Upregulation of Cox2 in Human Degenerated Nucleus Pulposus: The Association of Inflammation with Intervertebral Disc Degeneration. *Mediators of Inflammation*. 2021;2021:2933199. doi:https://dx.doi.org/10.1155/2021/2933199

268. Liu T, Wang Y, Xu Z, Wu T, Zang X, Li M, et al. Application Study of 3d Lava-Flex on Lumbar Intervertebral Disc Degeneration. *European Journal of Medical Research*. May 07 2021;26(1):43. doi:https://dx.doi.org/10.1186/s40001-021-00512-y

269. Liu X, Pan F, Ba Z, Wang S, Wu D. The Potential Effect of Type 2 Diabetes Mellitus on Lumbar Disc Degeneration: A Retrospective Single-Center Study. *Journal of Orthopaedic Surgery*. Mar 14 2018;13(1):52. doi:https://dx.doi.org/10.1186/s13018-018-0755-8

270. Liu ZZ, Chen JY, Cai ZX, Jiang XH, Zhang Y, Yang ZH, et al. Mri of Lumbar Intervertebral Disc Degeneration: Correlation of Tlrho Value with Pfirrmann Grade and T2 Value. [Chinese]. *Chinese Journal of Medical Imaging Technology*. February 2014;30(2):260-264.

271. Liu ZZ, Wen HQ, Zhu YQ, Zhao BL, Kong QC, Chen JY, et al. Short-Term Effect of Lumbar Traction on Intervertebral Discs in Patients with Low Back Pain: Correlation between the T2 Value and Odi/Vas Score. Research Support, Non-U.S. Gov't. *Cartilage*. 12 2021;13(1_suppl):414S-423S. doi:https://dx.doi.org/10.1177/1947603521996793

272. Lorenc T, Burzykowski T. Relationship among the Foraminal Area and Demographic and Clinical Characteristics of Patients with Low Back Pain. *World Neurosurgery*. 04 2022;160:e520-e528. doi:https://dx.doi.org/10.1016/j.wneu.2022.01.062

273. Lorenc T, Glinkowski WM, Golebiowski M. Axially Loaded Magnetic Resonance Imaging Identification of the Factors Associated with Low Back-Related Leg Pain. *Journal of Clinical Medicine*. Aug 29 2021;10(17):29. doi:https://dx.doi.org/10.3390/jcm10173884

274. Louie PK, Orias AaE, Fogg LF, Labelle M, An HS, Andersson GBJ, et al. Changes in Lumbar Endplate Area and Concavity Associated with Disc Degeneration. *Spine*. 2018;43(19):E1127-E1134. doi:https://dx.doi.org/10.1097/BRS.0000000000002657

275. Ma H, Zhang X, Guo Y, Du Y, Wang R, Niu G. Intravoxel Incoherent Motion Mr Imaging in the Quantitative Evaluation of Lumbar Disc Degeneration. [Chinese]. b. *Journal of Xi'an Jiaotong University (Medical Sciences)*. 05 Nov 2020;41(6):901-905. doi:https://dx.doi.org/10.7652/jdyxb202006016

276. Ma J, Wang R, Yu Y, Xu X, Duan H, Yu N. Is Fractal Dimension a Reliable Imaging Biomarker for the Quantitative Classification of an Intervertebral Disk? Research Support, Non-U.S. Gov't. *European Spine Journal*. 05 2020;29(5):1175-1180. doi:https://dx.doi.org/10.1007/s00586-020-06370-2

277. Maasumi K, Tehranzadeh J, Muftuler LT, Gardner V, Hasso AN. Assessment of the Correlation between Apparent Diffusion Coefficient and Intervertebral Disk Degeneration Using 3 Tesla Mri. *Neuroradiology Journal*. 2011;24(4):593-602. doi:10.1177/197140091102400416

278. Maatta J, Kautiainen H, Leinonen V, Niinimaki J, Jarvenpaa S, Koskelainen T, et al. Association of Modic Changes with Health-Related Quality of Life among Patients Referred to Spine Surgery. *Scandinavian Journal of Pain*. Jan 01 2014;5(1):36-40. doi:https://dx.doi.org/10.1016/j.sjpain.2013.08.003

279. Maatta JH, Karppinen J, Paananen M, Bow C, Luk KDK, Cheung KMC, et al. Refined Phenotyping of Modic Changes: Imaging Biomarkers of Prolonged Severe Low Back Pain and Disability. Multicenter Study Observational Study. *Medicine*. May 2016;95(22):e3495.doi:https://dx.doi.org/10.1097/MD.0000000000003495

280. Maatta JH, Karppinen JI, Luk KD, Cheung KM, Samartzis D. Phenotype Profiling of Modic Changes of the Lumbar Spine and Its Association with Other Mri Phenotypes: A Large-Scale Population-Based Study. Evaluation Study Research Support, Non-U.S. Gov't. *Spine Journal: Official Journal of the North American Spine Society*. Sep 01 2015;15(9):1933-42. doi:https://dx.doi.org/10.1016/j.spinee.2015.06.056

281. Machino M, Nakashima H, Ito K, Tsushima M, Ando K, Kobayashi K, et al. Influence of Age and Gender on Intervertebral Disk Degeneration and Height in the Thoracolumbar Spine. *Spine Surgery and Related Research*. 2022;6(4):379-387. doi:https://dx.doi.org/10.22603/ssrr.2021-0187

282. Majeed SA, Seshadrinath NaK, Binoy KR, Raji L. Lumbar Disc Herniation: Is There an Association between Histological and Magnetic Resonance Imaging Findings? *Indian Journal of Orthopaedics*. 2016;50(3):234-242. doi:10.4103/0019-5413.181794

283. Mallow GM, Zepeda D, Kuzel TG, Barajas JN, Aboushaala K, Nolte MT, et al. Issls Prize in Clinical Science 2022: Epidemiology, Risk Factors and Clinical Impact of Juvenile Modic Changes in Paediatric Patients with Low Back Pain. *European Spine Journal*. 05 2022;31(5):1069-1079. doi:https://dx.doi.org/10.1007/s00586-022-07125-x

284. Manabe H, Sakai T, Omichi Y, Sugiura K, Morimoto M, Tezuka F, et al. Role of Growth Plate (Apophyseal Ring Fracture) in Causing Modic Type Changes in Pediatric Low Back Pain Patients. *European Spine Journal*. 09 2021;30(9):2565-2569. doi:https://dx.doi.org/10.1007/s00586-021-06885-2

285. Mardare M, Oprea M, Popa I, Zazgyva A, Niculescu M, Poenaru D. Sagittal Balance Parameters Correlate with Spinal Conformational Type and Mri Changes in Lumbar Degenerative Disc Disease: Results of a Retrospective Study. *European Journal of Orthopaedic Surgery & Traumatology*. 2016;26(7):735-743. doi:10.1007/s00590-016-1842-3

286. Marinelli NL, Haughton VM, Anderson PA. T2 Relaxation Times Correlated with Stage of Lumbar Intervertebral Disk Degeneration and Patient Age. Clinical Trial Validation Study. *Ajnr: American Journal of Neuroradiology*. Aug 2010;31(7):1278-82. doi:https://dx.doi.org/10.3174/ajnr.A2080

287. Martin JT, Oldweiler AB, Kosinski AS, Spritzer CE, Soher BJ, Erickson MM, et al. Lumbar Intervertebral Disc Diurnal Deformations and T2 and T1rho Relaxation Times Vary by Spinal Level and Disc Region. Research Support, N.I.H., Extramural Research Support, Non-U.S. Gov't. *European Spine Journal*. 03 2022;31(3):746-754. doi:https://dx.doi.org/10.1007/s00586-021-07097-4

288. Martin JT, Wesorick B, Oldweiler AB, Kosinski AS, Goode AP, Defrate LE. In Vivo Fluid Transport in Human Intervertebral Discs Varies by Spinal Level and Disc Region. *JOR Spine*. Jun 2022;5(2):e1199. doi:https://dx.doi.org/10.1002/jsp2.1199

289. Maurer E, Klinger C, Lorbeer R, Hefferman G, Schlett CL, Peters A, et al. Association between Cardiovascular Risk Factors and Degenerative Disc Disease of the Thoracolumbar Spine in the General Population: Results from the Kora Mri Study. *Acta Radiologica*. Jun 2022;63(6):750-759. doi:https://dx.doi.org/10.1177/02841851211010391

290. Maurer E, Klinger C, Lorbeer R, Rathmann W, Peters A, Schlett CL, et al. Long-Term Effect of Physical Inactivity on Thoracic and Lumbar Disc Degeneration-an Mri-Based Analysis of 385 Individuals from the General Population. Research Support, Non-U.S. Gov't. *Spine Journal: Official Journal of the North American Spine Society*. 09 2020;20(9):1386-1396. doi:https://dx.doi.org/10.1016/j.spinee.2020.04.016

291. Mazza E, Marcia S, Mondaini F, Piras E, Giordan N, Torri T, et al. Efficacy and Safety of a Novel Hydrogel (Hyadd4-G) in Degenerative Disc Disease Patients: A Multicentric Open Label Study. Clinical Trial Multicenter Study Research Support, Non-U.S. Gov't. *European Review for Medical & Pharmacological Sciences*. 03 2020;24(5):2692-2703. doi:https://dx.doi.org/10.26355/eurrev_202003_20539

292. Mcsweeney TP, Tiulpin A, Saarakkala S, Niinimaki J, Windsor R, Jamaludin A, et al. External Validation of Spinenet, an Open-Source Deep Learning Model for Grading Lumbar Disk Degeneration Mri Features, Using the Northern Finland Birth Cohort 1966. Observational Study. *Spine*. Apr 01 2023;48(7):484-491. doi:https://dx.doi.org/10.1097/BRS.0000000000004572

293. Meadows KD, Peloquin JM, Newman HR, Cauchy PJK, Vresilovic EJ, Elliott DM. Mri-Based Measurement of in Vivo Disc Mechanics in a Young Population Due to Flexion, Extension, and Diurnal Loading. *JOR Spine*. Mar 2023;6(1):e1243. doi:https://dx.doi.org/10.1002/jsp2.1243

294. Menezes-Reis R, Bonugli GP, Dalto VF, Da Silva Herrero CFP, Aparecido Defino HL, Nogueira-Barbosa MH, et al. Association between Lumbar Spine Sagittal Alignment and L4-L5 Disc Degeneration among Asymptomatic Young Adults. *Spine (03622436)*. 2016;41(18):E1081-E1087. doi:10.1097/BRS.0000000000001568

295. Menezes-Reis R, Salmon CE, Carvalho CS, Bonugli GP, Chung CB, Nogueira-Barbosa MH. T1rho and T2 Mapping of the Intervertebral Disk: Comparison of Different Methods of Segmentation. Comparative Study

Research Support, Non-U.S. Gov't. *Ajnr: American Journal of Neuroradiology*. Mar 2015;36(3):606-11. doi:https://dx.doi.org/10.3174/ajnr.A4125

296. Mera Y, Teraguchi M, Hashizume H, Oka H, Muraki S, Akune T, et al. Association between Types of Modic Changes in the Lumbar Region and Low Back Pain in a Large Cohort: The Wakayama Spine Study. Research Support, Non-U.S. Gov't Research Support, U.S. Gov't, Non-P.H.S. *European Spine Journal*. 04 2021;30(4):1011-1017. doi:https://dx.doi.org/10.1007/s00586-020-06618-x

297. Mertimo T, Karppinen J, Niinimaki J, Blanco R, Maatta J, Kankaanpaa M, et al. Association of Lumbar Disc Degeneration with Low Back Pain in Middle Age in the Northern Finland Birth Cohort 1966. *BMC Musculoskeletal Disorders*. Apr 15 2022;23(1):359. doi:https://dx.doi.org/10.1186/s12891-022-05302-z

298. Mesregah MK, Lee H, Roberts S, Gardner C, Shah I, Buchanan IA, et al. Evaluation of Facet Joints and Segmental Motion in Patients with Different Grades of L5/S1 Intervertebral Disc Degeneration: A Kinematic Mri Study. *European Spine Journal*. 10 2020;29(10):2609-2618. doi:https://dx.doi.org/10.1007/s00586-020-06482-9

299. Michopoulou S, Costaridou L, Vlychou M, Speller R, Todd-Pokropek A. Texture-Based Quantification of Lumbar Intervertebral Disc Degeneration from Conventional T2-Weighted Mri. Research Support, Non-U.S. Gov't. *Acta Radiologica*. Feb 01 2011;52(1):91-8. doi:https://dx.doi.org/10.1258/ar.2010.100166

300. Middendorp M, Vogl TJ, Kollias K, Kafchitsas K, Khan MF, Maataoui A. Association between Intervertebral Disc Degeneration and the Oswestry Disability Index. *Journal of Back & Musculoskeletal Rehabilitation*. 2017;30(4):819-823. doi:https://dx.doi.org/10.3233/BMR-150516

301. Miki T, Naoki F, Takashima H, Takebayashi T. Associations between Paraspinal Muscle Morphology, Disc Degeneration, and Clinical Features in Patients with Lumbar Spinal Stenosis. *Progress in Rehabilitation Medicine*. 2020;5:20200015. doi:https://dx.doi.org/10.2490/prm.20200015

302. Milgrom Y, Milgrom C, Constantini N, Applbaum Y, Radeva-Petrova D, Finestone AS. The Effect of Very High Versus Very Low Sustained Loading on the Lower Back and Knees in Middle Life. *BioMed Research International*. 2013;2013:921830. doi:https://dx.doi.org/10.1155/2013/921830

303. Min HK, He W, Tsai YD, Chen NF, Keorochana G, Do DH, et al. Relationship of Facet Tropism with Degeneration and Stability of Functional Spinal Unit. *Yonsei Medical Journal*. October 2009;50(5):624-629. doi:https://dx.doi.org/10.3349/ymj.2009.50.5.624

304. Min HK, Hymanson HJ, Kwan YS, Dong KC, Yong EC, Do HY, et al. Kinetic Magnetic Resonance Imaging Analysis of Abnormal Segmental Motion of the Functional Spine Unit: Clinical Article. *Journal of Neurosurgery: Spine*. April 2009;10(4):357-365. doi:https://dx.doi.org/10.3171/2008.12.SPINE08321

305. Minetama M, Kawakami M, Teraguchi M, Matsuo S, Enyo Y, Nakagawa M, et al. Mri Grading of Spinal Stenosis Is Not Associated with the Severity of Low Back Pain in Patients with Lumbar Spinal Stenosis. *BMC Musculoskeletal Disorders*. Sep 12 2022;23(1):857. doi:https://dx.doi.org/10.1186/s12891-022-05810-y

306. Minetama M, Kawakami M, Teraguchi M, Matsuo S, Sumiya T, Nakagawa M, et al. Endplate Defects, Not the Severity of Spinal Stenosis, Contribute to Low Back Pain in Patients with Lumbar Spinal Stenosis. *The spine journal : official journal of the North American Spine Society.* 2021;29doi:https://dx.doi.org/10.1016/j.spinee.2021.09.008

307. Morishita Y, Buser Z, D'oro A, Shiba K, Wang JC. Clinical Relationship of Degenerative Changes between the Cervical and Lumbar Spine. *Asian Spine Journal*. Apr 2018;12(2):343-348. doi:https://dx.doi.org/10.4184/asj.2018.12.2.343

308. Morishita Y, Ohta H, Naito M, Matsumoto Y, Huang G, Tatsumi M, et al. Kinematic Evaluation of the Adjacent Segments after Lumbar Instrumented Surgery: A Comparison between Rigid Fusion and Dynamic Non-Fusion Stabilization. Comparative Study. *European Spine Journal*. Sep 2011;20(9):1480-5. doi:https://dx.doi.org/10.1007/s00586-011-1701-1

309. Moser M, Adl Amini D, Albertini Sanchez L, Oezel L, Haffer H, Muellner M, et al. The Association between Vertebral Endplate Defects, Subchondral Bone Marrow Changes, and Lumbar Intervertebral Disc Degeneration: A Retrospective, 3-Year Longitudinal Study. *European Spine Journal*. Feb 11 2023;11:11. doi:https://dx.doi.org/10.1007/s00586-023-07544-4

310. Muellner M, Haffer H, Chiapparelli E, Dodo Y, Tan ET, Shue J, et al. Differences in Lumbar Paraspinal Muscle Morphology in Patients with Sagittal Malalignment Undergoing Posterior Lumbar Fusion Surgery. Research Support, N.I.H., Extramural. *European Spine Journal*. 11 2022;31(11):3109-3118. doi:https://dx.doi.org/10.1007/s00586-022-07351-3

311. Muftuler LT, Jarman JP, Yu HJ, Gardner VO, Maiman DJ, Arpinar VE. Association between Intervertebral Disc Degeneration and Endplate Perfusion Studied by Dce-Mri. Research Support, Non-U.S. Gov't. *European Spine Journal*. Apr 2015;24(4):679-85. doi:https://dx.doi.org/10.1007/s00586-014-3690-3

312. Munarriz PM, Paredes I, Alen JF, Castano-Leon AM, Cepeda S, Hernandez-Lain A, et al. [Assessment of the Correlation between Histological Degeneration and Radiological and Clinical Parameters in a Series of Patients Who Underwent Lumbar Disc Herniation Surgery]. Research Support, Non-U.S. Gov't. *Neurocirugia (English Edition)*. Mar - Apr 2018;29(2):79-85. Evaluacion de la correlacion entre degeneracion histologica y parametros radiologicos o clinicos en una serie de pacientes operados por hernia discal lumbar. doi:https://dx.doi.org/10.1016/j.neucir.2017.07.003

313. Munns JJ, An HS, Oias AaE, Andersson GJ, Takatori BR, Inoue N. Ligamentum Flavum Hyperthropy Related to Disc Degeneration. Conference Abstract. *Spine Journal*. September 2010;1):76S. doi:https://dx.doi.org/10.1016/j.spinee.2010.07.206

314. Murata K, Akeda K, Takegami N, Cheng K, Masuda K, Sudo A. Morphology of Intervertebral Disc Ruptures Evaluated by Vacuum Phenomenon Using Multi-Detector Computed Tomography: Association with Lumbar Disc Degeneration and Canal Stenosis. *BMC Musculoskeletal Disorders*. May 24 2018;19(1):164. doi:https://dx.doi.org/10.1186/s12891-018-2086-7

315. Nagashima M, Abe H, Amaya K, Matsumoto H, Yanaihara H, Nishiwaki Y, et al. A Method for Quantifying Intervertebral Disc Signal Intensity on T2-Weighted Imaging. *Acta Radiologica*. Nov 01 2012;53(9):1059-65. doi:https://dx.doi.org/10.1258/ar.2012.120039

316. Niemeyer F, Galbusera F, Tao Y, Kienle A, Beer M, Wilke HJ. A Deep Learning Model for the Accurate and Reliable Classification of Disc Degeneration Based on Mri Data. Research Support, Non-U.S. Gov't. *Investigative Radiology*. 02 01 2021;56(2):78-85. doi:https://dx.doi.org/10.1097/RLI.0000000000000709

317. Niinimaki JL, Parviainen O, Ruohonen J, Ojala RO, Kurunlahti M, Karppinen J, et al. In Vivo Quantification of Delayed Gadolinium Enhancement in the Nucleus Pulposus of Human Intervertebral Disc. *Journal of Magnetic Resonance Imaging*. Oct 2006;24(4):796-800.

318. Niu G, Yang J, Wang R, Dang S, Wu EX, Guo Y. Mr Imaging Assessment of Lumbar Intervertebral Disk Degeneration and Age-Related Changes: Apparent Diffusion Coefficient Versus T2 Quantitation. Controlled Clinical Trial Research Support, Non-U.S. Gov't. *Ajnr: American Journal of Neuroradiology*. Oct 2011;32(9):1617-23. doi:https://dx.doi.org/10.3174/ajnr.A2556

319. Nohara A, Kawakami N, Seki K, Tsuji T, Ohara T, Saito T, et al. The Effects of Spinal Fusion on Lumbar Disc Degeneration in Patients with Adolescent Idiopathic Scoliosis: A Minimum 10-Year Follow-Up. *Spine Deformity*. September 2015;3(5):462-468. doi:https://dx.doi.org/10.1016/j.jspd.2015.04.001

320. Nordberg CL, Boesen M, Fournier GL, Bliddal H, Hansen P, Hansen BB. Positional Changes in Lumbar Disc Herniation During Standing or Lumbar Extension: A Cross-Sectional Weight-Bearing Mri Study. *European Radiology*. Feb 2021;31(2):804-812. doi:https://dx.doi.org/10.1007/s00330-020-07132-w

321. Ohashi M, Watanabe K, Hirano T, Hasegawa K, Katsumi K, Tashi H, et al. Impact of the Flexibility of the Spinal Deformity on Low Back Pain and Disc Degeneration in Adult Patients Nonoperatively Treated for Adolescent Idiopathic Scoliosis with Thoracolumbar or Lumbar Curves. Research Support, Non-U.S. Gov't. *Spine Deformity*. 01 2022;10(1):133-140. doi:https://dx.doi.org/10.1007/s43390-021-00402-7

322. Oldweiler AB, Martin JT. In Vivo Relationships between Lumbar Facet Joint and Intervertebral Disc Composition and Diurnal Deformation. Research Support, N.I.H., Extramural. *Clinical Biomechanics*. 08 2021;88:105425. doi:https://dx.doi.org/10.1016/j.clinbiomech.2021.105425

323. Oprea M, Popa I, Cimpean AM, Raica M, Poenaru DV. Microscopic Assessment of Degenerated Intervertebral Disc: Clinical Implications and Possible Therapeutic Challenge. *In Vivo*. 01 Jan 2015;29(1):95-102.

324. Otluoglu GD, Konya D, Toktas ZO. The Influence of Mechanic Factors Idisc Degeneration Disease as a Determinant for Surgical Indication. *Neurospine*. March 2020;17(1):215-220. doi:https://dx.doi.org/10.14245/ns.2040044.022

325. Ozcan-Eksi EE, Eksi MS, Akcal MA. Severe Lumbar Intervertebral Disc Degeneration Is Associated with Modic Changes and Fatty Infiltration in the Paraspinal Muscles at All Lumbar Levels, except for L1-L2: A Cross-Sectional Analysis of 50 Symptomatic Women and 50 Age-Matched Symptomatic Men. *World Neurosurgery*. Feb 2019;122:e1069-e1077. doi:https://dx.doi.org/10.1016/j.wneu.2018.10.229

326. Ozcan-Eksi EE, Eksi MS, Turgut VU, Canbolat C, Pamir MN. Reciprocal Relationship between Multifidus and Psoas at L4-L5 Level in Women with Low Back Pain. *British journal of neurosurgery*. 24 Jun 2020:1-9. doi:https://dx.doi.org/10.1080/02688697.2020.1783434

327. Ozcan-Eksi EE, Kara M, Berikol G, Orhun O, Turgut VU, Eksi MS. A New Radiological Index for the Assessment of Higher Body Fat Status and Lumbar Spine Degeneration. *Skeletal Radiology*. Jun 2022;51(6):1261-1271. doi:https://dx.doi.org/10.1007/s00256-021-03957-8

328. Ozcan-Eksi EE, Turgut VU, Kucuksuleymanoglu D, Eksi MS. Obesity Could Be Associated with Poor Paraspinal Muscle Quality at Upper Lumbar Levels and Degenerated Spine at Lower Lumbar Levels: Is This a Domino Effect? *Journal of Clinical Neuroscience*. Dec 2021;94:120-127. doi:https://dx.doi.org/10.1016/j.jocn.2021.10.005

329. Ozcan-Eksi EE, Yayla A, Orhun O, Turgut VU, Arslan HN, Eksi MS. Is the Distribution Pattern of Modic Changes in Vertebral End-Plates Associated with the Severity of Intervertebral Disc Degeneration?: A Cross-Sectional Analysis of 527 Caucasians. *World Neurosurgery*. 06 2021;150:e298-e304. doi:https://dx.doi.org/10.1016/j.wneu.2021.02.128

330. Paholpak P, Dedeogullari E, Lee C, Tamai K, Barkoh K, Sessumpun K, et al. Do Modic Changes, Disc Degeneration, Translation and Angular Motion Affect Facet Osteoarthritis of the Lumbar Spine. *European Journal of Radiology*. Jan 2018;98:193-199. doi:https://dx.doi.org/10.1016/j.ejrad.2017.11.023

331. Carrino JA, Lurie JD, Herzog R, Tosteson ANA, Tosteson TD, Carragee EJ, et al. Lumbar Spine: Reliability of Mr Imaging Findings. *Radiology*. 2009;250(1):161-170. doi:10.1148/radiol.2493071999

332. Duran S, Cavusoglu M, Hatipoglu HG, Sozmen Ciliz D, Sakman B. Association between Measures of Vertebral Endplate Morphology and Lumbar Intervertebral Disc Degeneration. *Canadian Association of Radiologists Journal*. May 2017;68(2):210-216. doi:https://dx.doi.org/10.1016/j.carj.2016.11.002

333. Pan J, Lu X, Yang G, Han Y, Tong X, Wang Y. Lumbar Disc Degeneration Was Not Related to Spine and Hip Bone Mineral Densities in Chinese: Facet Joint Osteoarthritis May Confound the Association. *Archives of Osteoporosis*. Dec 2017;12(1):20. doi:https://dx.doi.org/10.1007/s11657-017-0315-6

334. Papic M, Papic V, Kresoja M, Munteanu V, Mikov I, Cigic T. Relation between Grades of Intervertebral Disc Degeneration and Occupational Activities of Patients with Lumbar Disc Herniation. Povezanost stepena degeneracije intervertebralnih diskusa i radnih aktivnosti kod bolesnika sa lumbalnom diskus hernijom. *Vojnosanitetski Pregled*. 2017;74(12):1121-1127. doi:https://dx.doi.org/10.2298/VSP151112306P

335. Pappou IP, Cammisa FP, Jr., Girardi FP. Correlation of End Plate Shape on Mri and Disc Degeneration in Surgically Treated Patients with Degenerative Disc Disease and Herniated Nucleus Pulposus. Comparative Study. *Spine Journal: Official Journal of the North American Spine Society*. Jan-Feb 2007;7(1):32-8.

336. Park C, Ryu K, Jee W. Degenerative Changes of Discs and Facet Joints in Lumbar Total Disc Replacement Using Prodisc Ii: Minimum Two-Year Follow-Up. *Spine (03622436)*. 2008;33(16):1755-1761. doi:10.1097/brs.0b013e31817b8fed

337. Pfirrmann CWA, Metzdorf A, Elfering A, Hodler J, Boos N. Effect of Aging and Degeneraton on Disc Volume and Shape: A Quantitative Study in Asymptomatic Volunteers. *Journal of Orthopaedic Research*. May 2006;24(5):1086-1094. doi:https://dx.doi.org/10.1002/jor.20113

338. Pfirrmann CWA, Metzdorf A, Zanetti M, Hodler J, Boos N. Magnetic Resonance Classification of Lumbar Intervertebral Disc Degeneration. *Spine (Philadelphia, Pa. 1976)*. 2001;26(17):1873-1878. doi:10.1097/00007632-200109010-00011

339. Pinson H, Hallaert G, Herregodts P, Everaert K, Couvreur T, Caemaert J, et al. Outcome of Anterior Lumbar Interbody Fusion: A Retrospective Study of Clinical and Radiologic Parameters. *World Neurosurgery*. Jul 2017;103:772-779. doi:https://dx.doi.org/10.1016/j.wneu.2017.04.077

340. Rajasekaran S, Kanna RM, Senthil N, Raveendran M, Ranjani V, Cheung KM, et al. Genetic Susceptibility of Lumbar Degenerative Disc Disease in Young Indian Adults. Research Support, Non-U.S. Gov't. *European Spine Journal*. Sep 2015;24(9):1969-75. doi:https://dx.doi.org/10.1007/s00586-014-3687-y

341. Raudner M, Schreiner MM, Juras V, Weber M, Stelzeneder D, Kronnerwetter C, et al. Prediction of Lumbar Disk Herniation and Clinical Outcome Using Quantitative Magnetic Resonance Imaging: A 5-Year Follow-up Study. *Investigative Radiology*. 01 Mar 2019;54(3):183-189. doi:https://dx.doi.org/10.1097/RLI.0000000000000527

342. Raudner M, Toth DF, Schreiner MM, Hilbert T, Kober T, Juras V, et al. Synthetic T<Sub>2</Sub>-Weighted Images of the Lumbar Spine Derived from an Accelerated T<Sub>2</Sub> Mapping Sequence: Comparison to Conventional T<Sub>2</Sub>W Turbo Spin Echo. Research Support, Non-U.S. Gov't. *Magnetic Resonance Imaging*. 12 2021;84:92-100. doi:https://dx.doi.org/10.1016/j.mri.2021.09.011

343. Ravikanth R. Magnetic Resonance Evaluation of Lumbar Disc Degenerative Disease as an Implication of Low Back Pain: A Prospective Analysis. *Neurology India*. Nov-Dec 2020;68(6):1378-1384. doi:https://dx.doi.org/10.4103/0028-3886.304091

344. Reyes-Sanchez A, Zarate-Kalfopulos B, Ramirez-Mora I, Rosales-Olivarez LM, Alpizar-Aguirre A, Sanchez-Bringas G. Posterior Dynamic Stabilization of the Lumbar Spine with the Accuflex Rod System as a Stand-Alone Device: Experience in 20 Patients with 2-Year Follow-Up. *European Spine Journal*. Dec 2010;19(12):2164-70. doi:https://dx.doi.org/10.1007/s00586-010-1417-7

345. Rigal J, Thelen T, Byrne F, Cogniet A, Boissière L, Aunoble S, et al. Prospective Study Using Anterior Approach Did Not Show Association between Modic 1 Changes and Low Grade Infection in Lumbar Spine. *European Spine Journal*. 2016;25(4):1000-1005. doi:10.1007/s00586-016-4396-5

346. Roberts S, Gardner C, Jiang Z, Abedi A, Buser Z, Wang JC. Analysis of Trends in Lumbar Disc Degeneration Using Kinematic Mri. *Clinical Imaging*. Nov 2021;79:136-141. doi:https://dx.doi.org/10.1016/j.clinimag.2021.04.028

347. Rodriguez-Soto AE, Berry DB, Jaworski R, Jensen A, Chung CB, Niederberger B, et al. The Effect of Training on Lumbar Spine Posture and Intervertebral Disc Degeneration in Active-Duty Marines. Comparative Study. *Ergonomics*. Aug 2017;60(8):1055-1063. doi:https://dx.doi.org/10.1080/00140139.2016.1252858

348. Roller BL, Boutin RD, O'gara TJ, Knio ZO, Jamaludin A, Tan J, et al. Accurate Prediction of Lumbar Microdecompression Level with an Automated Mri Grading System. *Skeletal Radiology*. Jan 2021;50(1):69-78. doi:https://dx.doi.org/10.1007/s00256-020-03505-w

349. Ruangchainikom M, Daubs MD, Suzuki A, Xiong C, Hayashi T, Scott TP, et al. Patterns of Lumbar Disc Degeneration: Magnetic Resonance Imaging Analysis in Symptomatic Subjects. *Asian Spine Journal*. Dec 2021;15(6):799-807. doi:https://dx.doi.org/10.31616/asj.2020.0325

350. Salamat S, Hutchings J, Kwong C, Magnussen J, Hancock MJ. The Relationship between Quantitative Measures of Disc Height and Disc Signal Intensity with Pfirrmann Score of Disc Degeneration. *Springerplus*. 2016;5(1):829. doi:https://dx.doi.org/10.1186/s40064-016-2542-5

351. Salo S, Hurri H, Rikkonen T, Sund R, Kroger H, Sirola J. Association between Severe Lumbar Disc Degeneration and Self-Reported Occupational Physical Loading. *Journal of Occupational Health*. Jan 2022;64(1):e12316. doi:https://dx.doi.org/10.1002/1348-9585.12316

352. Salo S, Leinonen V, Rikkonen T, Vainio P, Marttila J, Honkanen R, et al. Association between Bone Mineral Density and Lumbar Disc Degeneration. Research Support, Non-U.S. Gov't. *Maturitas*. Dec 2014;79(4):449-55. doi:https://dx.doi.org/10.1016/j.maturitas.2014.09.003

353. Sandor Z, Rathonyi GK, Dinya E. Assessment of Lumbar Lordosis Distribution with a Novel Mathematical Approach and Its Adaptation for Lumbar Intervertebral Disc Degeneration. *Computational & Mathematical Methods in Medicine*. 2020;2020:7312125. doi:https://dx.doi.org/10.1155/2020/7312125

354. Sandor Z, Rathonyi GK, Dinya E. Relationship between the Distribution of Lumbar Lordosis and the Average Degeneration of Intervertebral Discs. [Hungarian]. A lumbalis lordosis eloszl s nak s a porckorongok tlagos degener ci j nak kapcsolata. *Orvosi Hetilap*. August 2020;161(31):1286-1292. doi:https://dx.doi.org/10.1556/650.2020.31794

355. Schroeder GD, Mendoza M, Daley E, La Bella C, Savage JW, Patel AA, et al. The Role of Athletic Activity on Structural Lumbar Abnormalities in Adolescent Patients with Symptomatic Low Back Pain. Conference Abstract. *Spine Journal*. 01 Nov 2014;1):S139. doi:https://dx.doi.org/10.1016/j.spinee.2014.08.338

356. Schwarz-Nemec U, Friedrich KM, Prayer D, Trattnig S, Schwarz FK, Weber M, et al. Lumbar Intervertebral Disc Degeneration as a Common Incidental Finding in Young Pregnant Women as Observed on Prenatal Magnetic Resonance Imaging. *Journal of Women's Health*. 05 2020;29(5):713-720. doi:https://dx.doi.org/10.1089/jwh.2019.7964

357. Seyithanoglu MH, Kitis S, Ozer OF, Kocyigit A, Dundar T, Gundag Papaker M, et al. Comparison of the Biochemical and Radiological Criteria for Lumbar Disc Degeneration. Comparative Study. *Neurologia i Neurochirurgia Polska*. Sep - Oct 2018;52(5):570-574. doi:https://dx.doi.org/10.1016/j.pjnns.2018.01.008

358. Sharma A, Pilgram T, Wippold FJ, 2nd. Association between Annular Tears and Disk Degeneration: A Longitudinal Study. *Ajnr: American Journal of Neuroradiology*. Mar 2009;30(3):500-6. doi:https://dx.doi.org/10.3174/ajnr.A1411

359. Sharma A, Sargar K, Salter A. Temporal Evolution of Disc in Young Patients with Low Back Pain and Stress Reaction in Lumbar Vertebrae. Observational Study. *Ajnr: American Journal of Neuroradiology*. Aug 2017;38(8):1647-1652. doi:https://dx.doi.org/10.3174/ajnr.A5237

360. Sharma A, Walk RE, Tang SY, Eldaya R, Owen PJ, Belavy DL. Variability of T2-Relaxation Times of Healthy Lumbar Intervertebral Discs Is More Homogeneous within an Individual Than across Healthy Individuals. Research Support, N.I.H., Extramural Research Support, Non-U.S. Gov't. *Ajnr: American Journal of Neuroradiology*. 11 2020;41(11):2160-2165. doi:https://dx.doi.org/10.3174/ajnr.A6791

361. Shu-Hua Y, Orías AaE, Chien-Chou P, Senoo I, Andersson GBJ, An HS, et al. Spatial Geometric and Magnetic Resonance Signal Intensity Changes with Advancing Stages of Nucleus Pulposus Degeneration. *BMC Musculoskeletal Disorders*. 2017;18:1-6. doi:10.1186/s12891-017-1838-0

362. Singh R, Kumar P, Wadhwani J, Yadav RK, Khanna M, Kaur S. A Comparative Study to Evaluate Disc Degeneration on Magnetic Resonance Imaging in Patients with Chronic Low Back Pain and Asymptomatic Individuals. *Journal of Orthopaedics, Trauma and Rehabilitation*. 2021;28(no pagination)doi:https://dx.doi.org/10.1177/22104917211039522

363. Smith A, Hancock M, O’hanlon S, Krieser M, O’sullivan P, Cicuttini F, et al. The Association between Different Trajectories of Low Back Pain and Degenerative Imaging Findings in Young Adult Participants within the Raine Study. *Spine (Philadelphia, Pa. 1976)*. 2022;47(3):269-276. doi:10.1097/BRS.0000000000004171

364. Soh J, Lee JC, Shin BJ. Analysis of Risk Factors for Adjacent Segment Degeneration Occurring More Than 5 Years after Fusion with Pedicle Screw Fixation for Degenerative Lumbar Spine. *Asian Spine Journal*. Dec 2013;7(4):273-81. doi:https://dx.doi.org/10.4184/asj.2013.7.4.273

365. Son S, Lee SG, Kim WK, Ahn Y, Jung JM. Disc Height Discrepancy between Supine and Standing Positions as a Screening Metric for Discogenic Back Pain in Patients with Disc Degeneration. *Spine Journal: Official Journal of the North American Spine Society*. 01 2021;21(1):71-79. doi:https://dx.doi.org/10.1016/j.spinee.2020.07.006

366. Song J, Pan F, Kong C, Sun X, Wang Y, Wang W, et al. Does the Sagittal Spinal Profile Differ between the Elderly Chinese Populations with and without Lumbar Disc Herniation? *Asian Journal of Surgery*. Dec 2022;45(12):2719-2724. doi:https://dx.doi.org/10.1016/j.asjsur.2022.03.020

367. Song Q, Liu X, Chen DJ, Lai Q, Tang B, Zhang B, et al. Evaluation of Mri and Ct Parameters to Analyze the Correlation between Disc and Facet Joint Degeneration in the Lumbar Three-Joint Complex. Observational Study. *Medicine*. Oct 2019;98(40):e17336. doi:https://dx.doi.org/10.1097/MD.0000000000017336

368. Splendiani A, Bruno F, Marsecano C, Arrigoni F, Di Cesare E, Barile A, et al. Modic I Changes Size Increase from Supine to Standing Mri Correlates with Increase in Pain Intensity in Standing Position: Uncovering the "Biomechanical Stress" and "Active Discopathy" Theories in Low Back Pain. *European Spine Journal*. 05 2019;28(5):983-992. doi:https://dx.doi.org/10.1007/s00586-019-05974-7

369. Stelzeneder D, Welsch GH, Kovacs BK, Goed S, Paternostro-Sluga T, Vlychou M, et al. Quantitative T2 Evaluation at 3.0t Compared to Morphological Grading of the Lumbar Intervertebral Disc: A Standardized Evaluation Approach in Patients with Low Back Pain. Comparative Study Research Support, Non-U.S. Gov't. *European Journal of Radiology*. Feb 2012;81(2):324-30. doi:https://dx.doi.org/10.1016/j.ejrad.2010.12.093

370. Stosch-Wiechert K, Wuertz-Kozak K, Hitzl W, Szeimies U, Stabler A, Siepe CJ. Clinical and Radiological Mid- to Long-Term Investigation of Anterior Lumbar Stand-Alone Fusion: Incidence of Reoperation and Adjacent Segment Degeneration. *Brain & Spine*. 2022;2:100924. doi:https://dx.doi.org/10.1016/j.bas.2022.100924

371. Su Y, Ren D, Liu D, Li J, Wang T, Qi W, et al. Effects of Endplate Healing Morphology on Intervertebral Disc Degeneration after Pedicle Screw Fixation for Thoracolumbar Fractures. Evaluation Study. *Medicine*. Apr 30 2021;100(17):e25636. doi:https://dx.doi.org/10.1097/MD.0000000000025636

372. Sudhir G, Jayabalan V, Sellayee S, Gadde S, Kailash K. Is There an Interdependence between Paraspinal Muscle Mass and Lumbar Disc Degeneration? A Mri Based Study at 2520 Levels in 504 Patients. *Journal of Clinical Orthopaedics & Trauma*. Nov 2021;22:101576. doi:https://dx.doi.org/10.1016/j.jcot.2021.101576

373. Sun S, Tan ET, Mintz DN, Sahr M, Endo Y, Nguyen J, et al. Evaluation of Deep Learning Reconstructed High-Resolution 3d Lumbar Spine Mri. *European Radiology*. Sep 2022;32(9):6167-6177. doi:https://dx.doi.org/10.1007/s00330-022-08708-4

374. Takahashi S, Lord EL, Hayashi T, Cohen JR, Lao L, Yao Q, et al. Radiologic Factors Associated with the Dynamic Change of Dural Sac Diameter in Lumbar Spine: A Kinematic Mri Study. *Clinical Spine Surgery : A Spine Publication*. Jul 2017;30(6):E827-E832. doi:https://dx.doi.org/10.1097/BSD.0000000000000403

375. Takashima H, Yoshimoto M, Ogon I, Takebayashi T, Imamura R, Akatsuka Y, et al. T1rho, T2, and T2* Relaxation Time Based on Grading of Intervertebral Disc Degeneration. *Acta Radiologica.* 2022;doi:https://dx.doi.org/10.1177/02841851221113936

376. Takashima H, Yoshimoto M, Ogon I, Terashima Y, Imamura R, Akatsuka Y, et al. Lumbar Disc Degeneration Assessment Using T2* Relaxation Time with Ultra-Short Te. *Magnetic Resonance Imaging*. November 2020;73:11-14. doi:https://dx.doi.org/10.1016/j.mri.2020.07.004

377. Takatalo J, Karppinen J, Nayha S, Taimela S, Niinimaki J, Blanco Sequeiros R, et al. Association between Adolescent Sport Activities and Lumbar Disk Degeneration among Young Adults. *Scandinavian Journal of Medicine & Science in Sports*. Dec 2017;27(12):1993-2001. doi:https://dx.doi.org/10.1111/sms.12840

378. Takatalo J, Karppinen J, Niinimäki J, Taimela S, Näyhä S, Järvelin MR, et al. Prevalence of Degenerative Imaging Findings in Lumbar Magnetic Resonance Imaging among Young Adults. *Spine (03622436)*. 2009;34(16):1716-1721. doi:10.1097/BRS.0b013e3181ac5fec

379. Takatalo J, Karppinen J, Niinimaki J, Taimela S, Nayha S, Mutanen P, et al. Does Lumbar Disc Degeneration on Magnetic Resonance Imaging Associate with Low Back Symptom Severity in Young Finnish Adults? Research Support, Non-U.S. Gov't. *Spine*. Dec 01 2011;36(25):2180-9. doi:https://dx.doi.org/10.1097/BRS.0b013e3182077122

380. Takatalo J, Karppinen J, Taimela S, Niinimaki J, Laitinen J, Blanco Sequeiros R, et al. Body Mass Index Is Associated with Lumbar Disc Degeneration in Young Finnish Males: Subsample of Northern Finland Birth Cohort Study 1986. Research Support, Non-U.S. Gov't. *BMC Musculoskeletal Disorders*. Mar 11 2013;14:87. doi:https://dx.doi.org/10.1186/1471-2474-14-87

381. Takatalo J, Karppinen J, Taimela S, Niinimaki J, Laitinen J, Sequeiros RB, et al. Association of Abdominal Obesity with Lumbar Disc Degeneration--a Magnetic Resonance Imaging Study. Research Support, Non-U.S. Gov't. *PLoS ONE [Electronic Resource]*. 2013;8(2):e56244. doi:https://dx.doi.org/10.1371/journal.pone.0056244

382. Takegami N, Akeda K, Murata K, Yamada J, Sudo A. Association between Non-Traumatic Vertebral Fractures and Adjacent Discs Degeneration: A Cross-Sectional Study and Literature Review. Review. *BMC Musculoskeletal Disorders*. Nov 27 2020;21(1):781. doi:https://dx.doi.org/10.1186/s12891-020-03814-0

383. Takeuchi M, Nagamachi A, Adachi K, Inoue K, Tamaki Y, Omichi Y, et al. Prevalence of High-Intensity Zones in the Lumbar Spine According to Age and Their Correlation with Other Degenerative Findings on Magnetic Resonance Imaging. *Spine Surgery & Related Research*. Oct 26 2018;2(4):299-303. doi:https://dx.doi.org/10.22603/ssrr.2017-0071

384. Tan Y, Aghdasi BG, Montgomery SR, Inoue H, Lu C, Wang JC. Kinetic Magnetic Resonance Imaging Analysis of Lumbar Segmental Mobility in Patients without Significant Spondylosis. *European Spine Journal*. Dec 2012;21(12):2673-9. doi:https://dx.doi.org/10.1007/s00586-012-2387-8

385. Tarnoki AD, Tarnoki DL, Olah C, Szily M, Kovacs DT, Dienes A, et al. Lumbar Spine Abnormalities in Patients with Obstructive Sleep Apnoea. Research Support, Non-U.S. Gov't. *Scientific Reports*. 08 10 2021;11(1):16233. doi:https://dx.doi.org/10.1038/s41598-021-95667-3

386. Teichtahl AJ, Finnin MA, Wang Y, Wluka AE, Urquhart DM, O'sullivan R, et al. The Natural History of Modic Changes in a Community-Based Cohort. *Joint, Bone, Spine: Revue du Rhumatisme*. Mar 2017;84(2):197-202. doi:https://dx.doi.org/10.1016/j.jbspin.2016.03.011

387. Teichtahl AJ, Urquhart DM, Wang Y, Wluka AE, Heritier S, Cicuttini FM. A Dose-Response Relationship between Severity of Disc Degeneration and Intervertebral Disc Height in the Lumbosacral Spine. Research Support, Non-U.S. Gov't. *Arthritis Research & Therapy*. Oct 23 2015;17:297. doi:https://dx.doi.org/10.1186/s13075-015-0820-1

388. Teichtahl AJ, Urquhart DM, Wang Y, Wluka AE, O'sullivan R, Jones G, et al. Lumbar Disc Degeneration Is Associated with Modic Change and High Paraspinal Fat Content - a 3.0t Magnetic Resonance Imaging Study. Research Support, Non-U.S. Gov't. *BMC Musculoskeletal Disorders*. 10 21 2016;17(1):439.

389. Teraguchi M, Cheung JPY, Karppinen J, Bow C, Hashizume H, Luk KDK, et al. Lumbar High-Intensity Zones on Mri: Imaging Biomarkers for Severe, Prolonged Low Back Pain and Sciatica in a Population-Based Cohort. Research Support, Non-U.S. Gov't. *Spine Journal: Official Journal of the North American Spine Society*. 07 2020;20(7):1025-1034. doi:https://dx.doi.org/10.1016/j.spinee.2020.02.015

390. Teraguchi M, Samartzis D, Hashizume H, Yamada H, Muraki S, Oka H, et al. Classification of High Intensity Zones of the Lumbar Spine and Their Association with Other Spinal Mri Phenotypes: The Wakayama Spine Study. *PLoS ONE [Electronic Resource]*. 2016;11(9):e0160111. doi:https://dx.doi.org/10.1371/journal.pone.0160111

391. Teraguchi M, Yoshimura N, Hashizume H, Muraki S, Yamada H, Oka H, et al. Metabolic Syndrome Components Are Associated with Intervertebral Disc Degeneration: The Wakayama Spine Study. *PLoS ONE*. 01 Feb 2016;11(2) (no pagination)e0147565. doi:https://dx.doi.org/10.1371/journal.pone.0147565

392. Teraguchi M, Yoshimura N, Hashizume H, Muraki S, Yamada H, Oka H, et al. The Association of Combination of Disc Degeneration, End Plate Signal Change, and Schmorl Node with Low Back Pain in a Large Population Study: The Wakayama Spine Study. Research Support, Non-U.S. Gov't. *Spine Journal: Official Journal of the North American Spine Society*. Apr 01 2015;15(4):622-8. doi:https://dx.doi.org/10.1016/j.spinee.2014.11.012

393. Teraguchi M, Yoshimura N, Hashizume H, Yamada H, Oka H, Minamide A, et al. Progression, Incidence, and Risk Factors for Intervertebral Disc Degeneration in a Longitudinal Population-Based Cohort: The Wakayama Spine Study. Research Support, Non-U.S. Gov't. *Osteoarthritis & Cartilage*. 07 2017;25(7):1122-1131. doi:https://dx.doi.org/10.1016/j.joca.2017.01.001

394. Tonosu J, Oka H, Higashikawa A, Okazaki H, Tanaka S, Matsudaira K. The Associations between Magnetic Resonance Imaging Findings and Low Back Pain: A 10-Year Longitudinal Analysis. *PLoS ONE [Electronic Resource]*. 2017;12(11):e0188057. doi:https://dx.doi.org/10.1371/journal.pone.0188057

395. Tonosu J, Oka H, Matsudaira K, Higashikawa A, Okazaki H, Tanaka S. The Relationship between Findings on Magnetic Resonance Imaging and Previous History of Low Back Pain. *Journal of pain research*. 2017;10:47-52. doi:https://dx.doi.org/10.2147/JPR.S122380

396. Toren L, Hebelka H, Kasperska I, Brisby H, Lagerstrand K. With Axial Loading During Mri Diurnal T2-Value Changes in Lumbar Discs Are Neglectable: A Cross Sectional Study. *BMC Musculoskeletal Disorders*. 22 Jan 2018;19(1) (no pagination)25. doi:https://dx.doi.org/10.1186/s12891-018-1930-0

397. Torrie PaG, Mckay G, Bryne R, Morris SJ, Harding IJ. The Influence of Lumbar Spine Subtype on Lumbar Intervertebral Disc Degeneration in Young and Middle-Aged Adults. Conference Abstract. *European Spine Journal*. April 2014;1):S131. doi:https://dx.doi.org/10.1007/s00586-014-3199-9

398. Udby PM, Ohrt-Nissen S, Bendix T, Brorson S, Carreon LY, Andersen MO. The Association of Mri Findings and Long-Term Disability in Patients with Chronic Low Back Pain. *Global Spine Journal*. Jun 2021;11(5):633-639. doi:https://dx.doi.org/10.1177/2192568220921391

399. Urrutia J, Besa P, Campos M, Cikutovic P, Cabezon M, Molina M, et al. The Pfirrmann Classification of Lumbar Intervertebral Disc Degeneration: An Independent Inter- and Intra-Observer Agreement Assessment. *European Spine Journal*. 09 2016;25(9):2728-33. doi:https://dx.doi.org/10.1007/s00586-016-4438-z

400. Urrutia J, Besa P, Lobos D, Campos M, Arrieta C, Andia M, et al. Lumbar Paraspinal Muscle Fat Infiltration Is Independently Associated with Sex, Age, and Inter-Vertebral Disc Degeneration in Symptomatic Patients. *Skeletal Radiology*. Jul 2018;47(7):955-961. doi:https://dx.doi.org/10.1007/s00256-018-2880-1

401. Urrutia J, Zamora T, Prada C. The Prevalence of Degenerative or Incidental Findings in the Lumbar Spine of Pediatric Patients: A Study Using Magnetic Resonance Imaging as a Screening Tool. *European Spine Journal*. Feb 2016;25(2):596-601. doi:https://dx.doi.org/10.1007/s00586-015-4099-3

402. Vadala G, Russo F, Battisti S, Stellato L, Martina F, Del Vescovo R, et al. Early Intervertebral Disc Degeneration Changes in Asymptomatic Weightlifters Assessed by T1rho-Magnetic Resonance Imaging. *Spine*. Oct 15 2014;39(22):1881-6. doi:https://dx.doi.org/10.1097/BRS.0000000000000554

403. Vaga S, Brayda-Bruno M, Perona F, Fornari M, Raimondi MT, Petruzzi M, et al. Molecular Mr Imaging for the Evaluation of the Effect of Dynamic Stabilization on Lumbar Intervertebral Discs. Evaluation Study Research Support, Non-U.S. Gov't. *European Spine Journal*. Jun 2009;18 Suppl 1:40-8. doi:https://dx.doi.org/10.1007/s00586-009-0996-7

404. Van Den Heuvel MM, Oei EHG, Renkens JJM, Bierma-Zeinstra SMA, Van Middelkoop M. Structural Spinal Abnormalities on Mri and Associations with Weight Status in a General Pediatric Population. *The spine journal : official journal of the North American Spine Society.* 2020;09doi:https://dx.doi.org/10.1016/j.spinee.2020.10.003

405. Van Den Heuvel MM, Oei EHG, Renkens JJM, Bierma-Zeinstra SMA, Van Middelkoop M. Structural Spinal Abnormalities on Mri and Associations with Weight Status in a General Pediatric Population. Research Support, Non-U.S. Gov't. *Spine Journal: Official Journal of the North American Spine Society*. 03 2021;21(3):465-476. doi:https://dx.doi.org/10.1016/j.spinee.2020.10.003

406. Videbaek TS, Egund N, Christensen FB, Grethe Jurik A, Bunger CE. Adjacent Segment Degeneration after Lumbar Spinal Fusion: The Impact of Anterior Column Support: A Randomized Clinical Trial with an Eight- to Thirteen-Year Magnetic Resonance Imaging Follow-Up. Randomized Controlled Trial. *Spine*. Oct 15 2010;35(22):1955-64. doi:https://dx.doi.org/10.1097/BRS.0b013e3181e57269

407. Videman T, Battie MC, Gibbons LE, Gill K. A New Quantitative Measure of Disc Degeneration. *Spine Journal: Official Journal of the North American Spine Society*. 05 2017;17(5):746-753. doi:https://dx.doi.org/10.1016/j.spinee.2017.02.002

408. Violante FS, Zompatori M, Lovreglio P, Apostoli P, Marinelli F, Bonfiglioli R. Is Age More Than Manual Material Handling Associated with Lumbar Vertebral Body and Disc Changes? A Cross-Sectional Multicentre Mri Study. Multicenter Study Observational Study. *BMJ Open*. 09 18 2019;9(9):e029657. doi:https://dx.doi.org/10.1136/bmjopen-2019-029657

409. Waldenberg C, Hebelka H, Brisby H, Lagerstrand KM. Mri Histogram Analysis Enables Objective and Continuous Classification of Intervertebral Disc Degeneration. Research Support, Non-U.S. Gov't. *European Spine Journal*. 05 2018;27(5):1042-1048. doi:https://dx.doi.org/10.1007/s00586-017-5264-7

410. Walter BA, Mageswaran P, Mo X, Boulter DJ, Mashaly H, Nguyen XV, et al. Mr Elastography-Derived Stiffness: A Biomarker for Intervertebral Disc Degeneration. Research Support, N.I.H., Extramural Research Support, Non-U.S. Gov't. *Radiology*. 10 2017;285(1):167-175. doi:https://dx.doi.org/10.1148/radiol.2017162287

411. Walter SS, Lorbeer R, Hefferman G, Schlett CL, Peters A, Rospleszcz S, et al. Correlation between Thoracolumbar Disc Degeneration and Anatomical Spinopelvic Parameters in Supine Position on Mri. *PLoS ONE [Electronic Resource]*. 2021;16(6):e0252385. doi:https://dx.doi.org/10.1371/journal.pone.0252385

412. Wan ZY, Zhang J, Shan H, Liu TF, Song F, Samartzis D, et al. Epidemiology of Lumbar Degenerative Phenotypes of Children and Adolescents: A Large-Scale Imaging Study. *Global Spine Journal*. Apr 2023;13(3):599-608. doi:https://dx.doi.org/10.1177/21925682211000707

413. Wang J, Zhou Y, Zhang ZF, Li CQ, Zheng WJ, Liu J. Radiological Study on Disc Degeneration of Thoracolumbar Burst Fractures Treated by Percutaneous Pedicle Screw Fixation. *European Spine Journal*. Mar 2013;22(3):489-94. doi:https://dx.doi.org/10.1007/s00586-012-2462-1

414. Wang S, Yang D, Zheng G, Cao J, Zhao F, Shi J, et al. Mri Changes of Adjacent Segments after Transforaminal Lumbar Interbody Fusion (Tlif) and Foraminal Endoscopy: A Case-Control Study. *Medicine*. Oct 14 2022;101(41):e31093. doi:https://dx.doi.org/10.1097/MD.0000000000031093

415. Wang Y, Wang H, Lv F, Ma X, Xia X, Jiang J. Asymmetry between the Superior and Inferior Endplates Is a Risk Factor for Lumbar Disc Degeneration. Research Support, Non-U.S. Gov't. *Journal of Orthopaedic Research*. 09 2018;36(9):2469-2475. doi:https://dx.doi.org/10.1002/jor.23906

416. Wang ZX, An P, Li Y, Kim SH. Analysis of Lumbar Spine Mri in Asymptomatic Chinese Adults. [Chinese]. *Chinese Journal of Interventional Imaging and Therapy*. May 2012;9(5):371-375.

417. Wedatilake T, Palmer A, Fernquest S, Redgrave A, Arnold L, Kluzek S, et al. Association between Hip Joint Impingement and Lumbar Disc Disease in Elite Rowers. *BMJ Open Sport & Exercise Medicine*. 2021;7(4):e001063. doi:https://dx.doi.org/10.1136/bmjsem-2021-001063

418. Welsch GH, Trattnig S, Paternostro-Sluga T, Bohndorf K, Goed S, Stelzeneder D, et al. Parametric T2 and T2 Mapping Techniques to Visualize Intervertebral Disc Degeneration in Patients with Low Back Pain: Initial Results on the Clinical Use of 3.0 Tesla Mri. Clinical Trial Research Support, Non-U.S. Gov't. *Skeletal Radiology*. May 2011;40(5):543-51. doi:https://dx.doi.org/10.1007/s00256-010-1036-8

419. Wollschlager LM, Nebelung S, Schleich C, Muller-Lutz A, Radke KL, Frenken M, et al. Evaluating Lumbar Intervertebral Disc Degeneration on a Compositional Level Using Chemical Exchange Saturation Transfer: Preliminary Results in Patients with Adolescent Idiopathic Scoliosis. *Diagnostics*. June 2021;11(6) (no pagination)934. doi:https://dx.doi.org/10.3390/diagnostics11060934

420. Wu LL, Liu LH, Rao SX, Wu PY, Zhou JJ. Ultrashort Time-to-Echo T2 and T2 Relaxometry for Evaluation of Lumbar Disc Degeneration: A Comparative Study. *BMC Musculoskeletal Disorders*. Jun 01 2022;23(1):524. doi:https://dx.doi.org/10.1186/s12891-022-05481-9

421. Xiao L, Ni C, Shi J, Wang Z, Wang S, Zhang J, et al. Analysis of Correlation between Vertebral Endplate Change and Lumbar Disc Degeneration. *Medical Science Monitor*. Oct 15 2017;23:4932-4938.

422. Xingwang Y, Fei C, Chuning D, Jeffrey W, Yanlin T, Yao X, et al. Kinetic Magnetic Resonance Imaging Analysis of Thoracolumbar Segmental Mobility in Patients without Significant Spondylosis. *Medicine*. 2020;99(2):1-6. doi:10.1097/MD.0000000000018202

423. Xiong X, Zhou Z, Figini M, Shangguan J, Zhang Z, Chen W. Multi-Parameter Evaluation of Lumbar Intervertebral Disc Degeneration Using Quantitative Magnetic Resonance Imaging Techniques. *American Journal Of Translational Research*. 2018;10(2):444-454.

424. Yabe Y, Hagiwara Y, Tsuchiya M, Onoda Y, Yoshida S, Onoki T, et al. Factors Associated with Thickening of the Ligamentum Flavum on Magnetic Resonance Imaging in Patients with Lumbar Spinal Canal Stenosis. *Spine*. Jul 15 2022;47(14):1036-1041. doi:https://dx.doi.org/10.1097/BRS.0000000000004341

425. Yang H, Liu H, Li Z, Zhang K, Wang J, Wang H, et al. Low Back Pain Associated with Lumbar Disc Herniation: Role of Moderately Degenerative Disc and Annulus Fibrous Tears. *International journal of clinical and experimental medicine*. 2015;8(2):1634-44.

426. Yang L, Sun C, Gong T, Li Q, Chen X, Zhang X. T1rho, T2 and T2 Mapping of Lumbar Intervertebral Disc Degeneration: A Comparison Study. *BMC Musculoskeletal Disorders*. Dec 27 2022;23(1):1135. doi:https://dx.doi.org/10.1186/s12891-022-06040-y

427. Yang S, Lassalle L, Mekki A, Appert G, Rannou F, Nguyen C, et al. Can T2-Weighted Dixon Fat-Only Images Replace T1-Weighted Images in Degenerative Disc Disease with Modic Changes on Lumbar Spine Mri? *European Radiology*. Dec 2021;31(12):9380-9389. doi:https://dx.doi.org/10.1007/s00330-021-07946-2

428. Yang Z, Griffith JF, Leung PC, Lee R, Yang Z, Griffith JF, et al. Effect of Osteoporosis on Morphology and Mobility of the Lumbar Spine. *Spine (03622436)*. 2009;34(3):E115-21. doi:10.1097/BRS.0b013e3181895aca

429. Yin R, Lord EL, Cohen JR, Buser Z, Lao L, Zhong G, et al. Distribution of Schmorl Nodes in the Lumbar Spine and Their Relationship with Lumbar Disk Degeneration and Range of Motion. *Spine*. Jan 01 2015;40(1):E49-53. doi:https://dx.doi.org/10.1097/BRS.0000000000000658

430. Yin R, Wang JC, Lord EL, Cohen JR, Takahashi S. Distribution of Schmorl's Nodes in the Lumbar Spine and Their Relationship with Lumbar Disc Degeneration and Range of Motion. Conference Abstract. *Spine Journal*. 01 Nov 2014;1):S108. doi:https://dx.doi.org/10.1016/j.spinee.2014.08.272

431. Yoon MA, Hong SJ, Kang CH, Ahn KS, Kim BH. T1rho and T2 Mapping of Lumbar Intervertebral Disc: Correlation with Degeneration and Morphologic Changes in Different Disc Regions. *Magnetic Resonance Imaging*. Sep 2016;34(7):932-9. doi:https://dx.doi.org/10.1016/j.mri.2016.04.024

432. Young-Min O, Jong-Pil E, Oh Y-M, Eun J-P. Clinical Impact of Sagittal Spinopelvic Parameters on Disc Degeneration in Young Adults. *Medicine*. 2015;94(42):1-5. doi:10.1097/MD.0000000000001833

433. Yu HJ, Bahri S, Gardner V, Muftuler LT. In Vivo Quantification of Lumbar Disc Degeneration: Assessment of Adc Value Using a Degenerative Scoring System Based on Pfirrmann Framework. Research Support, Non-U.S. Gov't. *European Spine Journal*. Nov 2015;24(11):2442-8. doi:https://dx.doi.org/10.1007/s00586-014-3721-0

434. Yu LP, Qian WW, Yin GY, Ren YX, Hu ZY. Mri Assessment of Lumbar Intervertebral Disc Degeneration with Lumbar Degenerative Disease Using the Pfirrmann Grading Systems. Research Support, Non-U.S. Gov't. *PLoS ONE [Electronic Resource]*. 2012;7(12):e48074. doi:https://dx.doi.org/10.1371/journal.pone.0048074

435. Yucekul A, Akpunarli B, Durbas A, Zulemyan T, Havlucu I, Ergene G, et al. Does Vertebral Body Tethering Cause Disc and Facet Joint Degeneration? A Preliminary Mri Study with Minimum Two Years Follow-Up. Research Support, Non-U.S. Gov't. *Spine Journal: Official Journal of the North American Spine Society*. 11 2021;21(11):1793-1801. doi:https://dx.doi.org/10.1016/j.spinee.2021.05.020

436. Zehra U, Cheung JPY, Bow C, Crawford RJ, Luk KDK, Lu W, et al. Spinopelvic Alignment Predicts Disc Calcification, Displacement, and Modic Changes: Evidence of an Evolutionary Etiology for Clinically-Relevant Spinal Phenotypes. *JOR Spine*. Mar 2020;3(1):e1083. doi:https://dx.doi.org/10.1002/jsp2.1083

437. Zeng F, Zha Y, Li L, Xing D, Gong W, Hu L, et al. A Comparative Study of Diffusion Kurtosis Imaging and T2 Mapping in Quantitative Detection of Lumbar Intervertebral Disk Degeneration. Comparative Study

Research Support, Non-U.S. Gov't. *European Spine Journal*. 09 2019;28(9):2169-2178. doi:https://dx.doi.org/10.1007/s00586-019-06007-z

438. Zhang F, Wang H, Xu H, Shao M, Lu F, Jiang J, et al. Radiologic Analysis of Kinematic Characteristics of Modic Changes Based on Lumbar Disc Degeneration Grade. *World Neurosurgery*. Jun 2018;114:e851-e856. doi:https://dx.doi.org/10.1016/j.wneu.2018.03.098

439. Zhang J, Zhao F, Wang FL, Yang YF, Zhang C, Cao Y, et al. Identification of Lumbar Disc Disease Hallmarks: A Large Cross-Sectional Study. *Springerplus*. 2016;5(1):1973. doi:https://dx.doi.org/10.1186/s40064-016-3662-7

440. Zhang K, Li M, Pei X, Yuan H. Regression between Mr Findings of Lumbar Elements and Chronic Low Back Pain. [Chinese]. *Chinese Journal of Radiology (China)*. 10 Dec 2014;48(12):1019-1023. doi:https://dx.doi.org/10.3760/cma.j.issn.1005-1201.2014.12.013

441. Zhang W, Ma X, Wang Y, Zhao J, Zhang X, Gao Y, et al. Assessment of Apparent Diffusion Coefficient in Lumbar Intervertebral Disc Degeneration. *European Spine Journal*. Sep 2014;23(9):1830-6. doi:https://dx.doi.org/10.1007/s00586-014-3285-z

442. Zhang X, Yang L, Gao F, Yuan Z, Lin X, Yao B, et al. Comparison of T1rho and T2* Relaxation Mapping in Patients with Different Grades of Disc Degeneration at 3t Mr. *Medical Science Monitor*. 03 Jul 2015;21:1934-1941. doi:https://dx.doi.org/10.12659/MSM.894406

443. Zhao B, Huang W, Lu X, Ma X, Wang H, Lu F, et al. Association between Roussouly Classification and Characteristics of Lumbar Degeneration. Research Support, Non-U.S. Gov't. *World Neurosurgery*. 07 2022;163:e565-e572. doi:https://dx.doi.org/10.1016/j.wneu.2022.04.032

444. Zhou L, Li C, Zhang H. Correlation between Bone Mineral Density of Different Sites and Lumbar Disc Degeneration in Postmenopausal Women. Apr 01 2022;1(13):e28947.

445. Zobel BB, Vadalà G, Del Vescovo R, Battisti S, Martina FM, Stellato L, et al. T1ρ Magnetic Resonance Imaging Quantification of Early Lumbar Intervertebral Disc Degeneration in Healthy Young Adults. *Spine (03622436)*. 2012;37(14):1224-1230. doi:10.1097/BRS.0b013e31824b2450

446. Zou J, Yang H, Miyazaki M, Morishita Y, Wei F, Mcgovern S, et al. Dynamic Bulging of Intervertebral Discs in the Degenerative Lumbar Spine. Nov 01 2009;1(23):2545-50.

447. Munir S, Freidin MB, Rade M, Maatta J, Livshits G, Williams FMK. Endplate Defect Is Heritable, Associated with Low Back Pain and Triggers Intervertebral Disc Degeneration: A Longitudinal Study from Twinsuk. Twin Study. *Spine*. Nov 01 2018;43(21):1496-1501. doi:https://dx.doi.org/10.1097/BRS.0000000000002721

448. Perry J, Haughton V, Anderson PA, Wu Y, Fine J, Mistretta C. The Value of T2 Relaxation Times to Characterize Lumbar Intervertebral Disks: Preliminary Results. *Ajnr: American Journal of Neuroradiology*. Feb 2006;27(2):337-42.

449. Rade M, Maatta JH, Freidin MB, Airaksinen O, Karppinen J, Williams FMK. Vertebral Endplate Defect as Initiating Factor in Intervertebral Disc Degeneration. *Spine*. 15 Mar 2018;43(6):412-419. doi:https://dx.doi.org/10.1097/BRS.0000000000002352

450. Togao O, Hiwatashi A, Wada T, Yamashita K, Kikuchi K, Tokunaga C, et al. A Qualitative and Quantitative Correlation Study of Lumbar Intervertebral Disc Degeneration Using Glycosaminoglycan Chemical Exchange Saturation Transfer, Pfirrmann Grade, and T1-Rho. Research Support, Non-U.S. Gov't. *Ajnr: American Journal of Neuroradiology*. 07 2018;39(7):1369-1375. doi:https://dx.doi.org/10.3174/ajnr.A5657

451. Wang YX, Zhao F, Griffith JF, Mok GS, Leung JC, Ahuja AT, et al. T1rho and T2 Relaxation Times for Lumbar Disc Degeneration: An in Vivo Comparative Study at 3.0-Tesla Mri. *European Radiology*. 2013;23(1):228-234. doi:10.1007/s00330-012-2591-2

452. Huang J, Shen H, Wu J, Hu X, Zhu Z, Lv X, et al. Spine Explorer: A Deep Learning Based Fully Automated Program for Efficient and Reliable Quantifications of the Vertebrae and Discs on Sagittal Lumbar Spine Mr Images. Research Support, Non-U.S. Gov't. *Spine Journal: Official Journal of the North American Spine Society*. 04 2020;20(4):590-599. doi:https://dx.doi.org/10.1016/j.spinee.2019.11.010

453. Chadha M, Srivastava A, Kumar V, Tandon A. Disc Degeneration in Lumbar Spine of Asymptomatic Young Adults: A Descriptive Cross-Sectional Study. *Indian Journal of Orthopaedics*. Jun 2022;56(6):1083-1089. doi:https://dx.doi.org/10.1007/s43465-022-00619-2

454. Chao L, Hongliang C, Liangwei M, Weiyang Y, Kejun Z, Feijun L, et al. Association between Menopause and Lumbar Disc Degeneration: An Mri Study of 1,566 Women and 1,382 Men. *Menopause (10723714)*. 2017;24(10):1136-1144. doi:10.1097/GME.0000000000000902

455. Coppock JA, Zimmer NE, Englander ZA, Danyluk ST, Kosinski AS, Spritzer CE, et al. In Vivo Intervertebral Disc Mechanical Deformation Following a Treadmill Walking "Stress Test" Is Inversely Related to T1rho Relaxation Time. Research Support, N.I.H., Extramural. *Osteoarthritis & Cartilage*. 01 2023;31(1):126-133. doi:https://dx.doi.org/10.1016/j.joca.2022.09.008

456. Coskun H, Turan A, Kaplanoglu H, Kaplanoglu V. Frequency of Hypoplasia of the Vertebral Body at L5, and Its Relationship with Degeneration in Patients with Low Back Pain. *Turkish Neurosurgery*. 2022;32(4):641-648. doi:https://dx.doi.org/10.5137/1019-5149.JTN.34728-21.2

457. Deane JA, Lim AKP, Mcgregor AH, Strutton PH. Understanding the Impact of Lumbar Disc Degeneration and Chronic Low Back Pain: A Cross-Sectional Electromyographic Analysis of Postural Strategy During Predicted and Unpredicted Postural Perturbations. Research Support, Non-U.S. Gov't. *PLoS ONE [Electronic Resource]*. 2021;16(4):e0249308. doi:https://dx.doi.org/10.1371/journal.pone.0249308

458. Dujic MK, Recnik G, Milcic M, Bosnjak E, Rupreht M. Mri Assessment of the Early Disc Degeneration Two Levels above Fused Lumbar Spine Segment: A Comparison after Unilateral and Bilateral Transforaminal Lumbar Interbody Fusion (Tlif) Procedure. *Journal of Clinical Medicine*. Jul 07 2022;11(14):07. doi:https://dx.doi.org/10.3390/jcm11143952

459. Garcia Isidro M, Ferreiro Perez A, Fernandez Lopez-Pelaez MS, Moeinvaziri M, Fernandez Garcia P. Differences in Mri Measurements of Lateral Recesses and Foramina in Degenerative Lumbar Segments in Upright Versus Decubitus Symptomatic Patients. *Radiologia*. Mar 01 2021;01:01. Cambios en recesos laterales y foramenes en segmentos degenerativos lumbares de pacientes sintomaticos estudiados mediante resonancia magnetica en decubito y bipedestacion. doi:https://dx.doi.org/10.1016/j.rx.2021.01.003

460. Guan J, Liu T, Yu X, Feng N, Jiang G, Li W, et al. Isobar Hybrid Dynamic Stabilization with Posterolateral Fusion in Mild and Moderate Lumbar Degenerative Disease. *BMC Musculoskeletal Disorders*. Mar 23 2023;24(1):217. doi:https://dx.doi.org/10.1186/s12891-023-06329-6

461. Guo R, Yang X, Zhong Y, Lai Q, Gao T, Lai F, et al. Correlations between Modic Change and Degeneration in 3-Joint Complex of the Lower Lumbar Spine: A Retrospective Study. Evaluation Study. *Medicine*. Sep 2018;97(38):e12496. doi:https://dx.doi.org/10.1097/MD.0000000000012496

462. Hafeez R, Memon I. Mri Grading of Lumbar Spine Degenerative Disc Disease Using a Modified Pfirrmann Grading System. *Journal of the Liaquat University of Medical and Health Sciences*. October-December 2022;21(4):281-284. doi:https://dx.doi.org/10.22442/jlumhs.2022.00956

463. Huang L, Liu Y, Ding Y, Wu X, Zhang N, Lai Q, et al. Quantitative Evaluation of Lumbar Intervertebral Disc Degeneration by Axial T2 Mapping. Observational Study. *Medicine*. Dec 2017;96(51):e9393. doi:https://dx.doi.org/10.1097/MD.0000000000009393

464. Jang HJ, Park JY, Parkkuh SU, Chin DK, Kim KS, Cho YE, et al. The Fate of Proximal Junctional Vertebral Fractures after Long-Segment Spinal Fixation: Are There Predictable Radiologic Characteristics for Revision Surgery? *Journal of Korean Neurosurgical Society*. 2021;64(3):437-446. doi:https://dx.doi.org/10.3340/jkns.2020.0236

465. Karadag MK, Akinci AT, Basak AT, Hekimoglu M, Yildirim H, Akyoldas G, et al. Preoperative Magnetic Resonance Imaging Abnormalities Predictive of Lumbar Herniation Recurrence after Surgical Repair. *World Neurosurgery*. 09 2022;165:e750-e756. doi:https://dx.doi.org/10.1016/j.wneu.2022.06.143

466. Kim KT, Lee DH, Cho DC, Sung JK, Kim YB. Preoperative Risk Factors for Recurrent Lumbar Disk Herniation in L5-S1. *Journal of Spinal Disorders & Techniques*. Dec 2015;28(10):E571-7. doi:https://dx.doi.org/10.1097/BSD.0000000000000041

467. Kim SJ, Lee TH, Lim SM. Prevalence of Disc Degeneration in Asymptomatic Korean Subjects. Part 1 : Lumbar Spine. *Journal of Korean Neurosurgical Society*. Jan 2013;53(1):31-8. doi:https://dx.doi.org/10.3340/jkns.2013.53.1.31

468. Lee JW, Kim HC, Kim SI, Min HK, Ha KY, Park HY, et al. Effects of Bone Cement Augmentation for Uppermost Instrumented Vertebra on Adjacent Disc Segment Degeneration in Lumbar Fusions. *World Neurosurgery*. Mar 2023;171:e31-e37. doi:https://dx.doi.org/10.1016/j.wneu.2022.11.014

469. Li L, Zhou Z, Xiong W, Fang J, Li Y, Jiao Z, et al. Characterization of the Microstructure of the Intervertebral Disc in Patients with Chronic Low Back Pain by Diffusion Kurtosis Imaging. *European Spine Journal*. Nov 2019;28(11):2517-2525. doi:https://dx.doi.org/10.1007/s00586-019-06095-x

470. Li R, Wang Z, Ma L, Yang D, Xie D, Zhang B, et al. Lumbar Vertebral Endplate Defects on Magnetic Resonance Imaging in Degenerative Spondylolisthesis: Novel Classification, Characteristics, and Correlative Factor Analysis. *World Neurosurgery*. September 2020;141:e423-e430. doi:https://dx.doi.org/10.1016/j.wneu.2020.05.163

471. Li X, Zhao R, Rudd S, Ding W, Yang S. Correlation Analysis between Tamoxifen and Lumbar Intervertebral Disc Degeneration: A Retrospective Case-Control Study. *Pain Research and Management*. 2022;2022 (no pagination)3330260. doi:https://dx.doi.org/10.1155/2022/3330260

472. Lin RH, Chen HC, Pan HC, Chen HT, Chang CC, Tzeng CY, et al. Efficacy of Percutaneous Endoscopic Lumbar Discectomy for Pediatric Lumbar Disc Herniation and Degeneration on Magnetic Resonance Imaging: Case Series and Literature Review. Review. *Journal of International Medical Research*. Jan 2021;49(1):300060520986685. doi:https://dx.doi.org/10.1177/0300060520986685

473. Liu HY, Zhou J, Wang B, Wang HM, Jin ZH, Zhu ZG, et al. Comparison of Topping-Off and Posterior Lumbar Interbody Fusion Surgery in Lumbar Degenerative Disease: A Retrospective Study. *Chinese Medical Journal*. 20 Nov 2012;125(22):3942-3946. doi:https://dx.doi.org/10.3760/cma.j.issn.0366-6999.2012.22.005

474. Lou C, Chen HL, Feng XZ, Xiang GH, Zhu SP, Tian NF, et al. Menopause Is Associated with Lumbar Disc Degeneration: A Review of 4230 Intervertebral Discs. *Climacteric*. Dec 2014;17(6):700-4. doi:https://dx.doi.org/10.3109/13697137.2014.933409

475. Luo Y, Wang J, Zhang H, Yue M, Lu Z, Sun B. Feasibility of Dual Energy Ct Virtual Non-Calcium Imaging for Evaluation on Lumbar Intervertebral Disc Degeneration. [Chinese]. Ct. *Chinese Journal of Medical Imaging Technology*. 20 Jul 2021;37(7):1064-1068. doi:https://dx.doi.org/10.13929/j.issn.1003-3289.2021.07.023

476. Pan W, Wang J, Liu J, Lu Y, Huang B. [Modified Mri Short Time Inversion Recovery Sequence Grading System for Lumbar Intervertebral Disc Degeneration]. *Chung-Kuo Hsiu Fu Chung Chien Wai Ko Tsa Chih/Chinese Journal of Reparative & Reconstructive Surgery*. Dec 2012;26(12):1430-4.

477. Pandit P, Talbott JF, Pedoia V, Dillon W, Majumdar S. T1rho and T2 -Based Characterization of Regional Variations in Intervertebral Discs to Detect Early Degenerative Changes. Controlled Clinical Trial

Research Support, Non-U.S. Gov't. *Journal of Orthopaedic Research*. 08 2016;34(8):1373-81. doi:https://dx.doi.org/10.1002/jor.23311

478. Rahmani MS, Takahashi S, Hoshino M, Takayama K, Sasaoka R, Tsujio T, et al. The Degeneration of Adjacent Intervertebral Discs Negatively Influence Union Rate of Osteoporotic Vertebral Fracture: A Multicenter Cohort Study. Multicenter Study. *Journal of Orthopaedic Science*. Jul 2018;23(4):627-634. doi:https://dx.doi.org/10.1016/j.jos.2018.03.008

479. Saifuddin A, Rajakulasingam R, Santiago R, Siddiqui M, Khoo M, Pressney I. Comparison of Lumbar Degenerative Disc Disease Using Conventional Fast Spin Echo T<Sub>2</Sub>W Mri and T<Sub>2</Sub> Fast Spin Echo Dixon Sequences. Comparative Study. *British Journal of Radiology*. May 01 2021;94(1121):20201438. doi:https://dx.doi.org/10.1259/bjr.20201438

480. Sezer C, Acikalin R. Unilateral Dynamic Stabilization in Recurrent Lumbar Disc Herniation. *Turkish Neurosurgery*. 2023;33(2):334-340. doi:https://dx.doi.org/10.5137/1019-5149.JTN.42533-22.2

481. Shinohara Y, Sasaki F, Ohmura T, Itoh T, Endo T, Kinoshita T. Evaluation of Lumbar Intervertebral Disc Degeneration Using Dual Energy Ct Virtual Non-Calcium Imaging. *European Journal of Radiology*. Mar 2020;124:108817. doi:https://dx.doi.org/10.1016/j.ejrad.2020.108817

482. Sun D, Liu P, Cheng J, Ma Z, Liu J, Qin T. Correlation between Intervertebral Disc Degeneration, Paraspinal Muscle Atrophy, and Lumbar Facet Joints Degeneration in Patients with Lumbar Disc Herniation. *BMC Musculoskeletal Disorders*. 04 20 2017;18(1):167. doi:https://dx.doi.org/10.1186/s12891-017-1522-4

483. Wang YL, Wang XY, Fang BD, Chi YL, Xu HZ, Wu LJ, et al. L5-S1 Disc Degeneration and the Anatomic Parameters of the Iliac Crest: Imaging Study. Research Support, Non-U.S. Gov't. *European Spine Journal*. Nov 2015;24(11):2481-7. doi:https://dx.doi.org/10.1007/s00586-015-4076-x

484. Wang YX, Griffith JF, Ma HT, Kwok AW, Leung JC, Yeung DK, et al. Relationship between Gender, Bone Mineral Density, and Disc Degeneration in the Lumbar Spine: A Study in Elderly Subjects Using an Eight-Level Mri-Based Disc Degeneration Grading System. *Osteoporosis International*. Jan 2011;22(1):91-6. doi:https://dx.doi.org/10.1007/s00198-010-1200-y

485. Wang YX, Kwok AW, Griffith JF, Leung JC, Ma HT, Ahuja AT, et al. Relationship between Hip Bone Mineral Density and Lumbar Disc Degeneration: A Study in Elderly Subjects Using an Eight-Level Mri-Based Disc Degeneration Grading System. Research Support, Non-U.S. Gov't. *Journal of Magnetic Resonance Imaging*. Apr 2011;33(4):916-20. doi:https://dx.doi.org/10.1002/jmri.22518

486. Wang YXJ, Griffith JF, Ma HT, Kwok AWL, Leung JCS, Yeung DKW, et al. Relationship between Gender, Bone Mineral Density, and Disc Degeneration in the Lumbar Spine: A Study in Elderly Subjects Using an Eight-Level Mri-Based Disc Degeneration Grading System. *Osteoporosis International*. January 2011;22(1):91-96. doi:https://dx.doi.org/10.1007/s00198-010-1200-y

487. Wei Z, Lombardi AF, Lee RR, Wallace M, Masuda K, Chang EY, et al. Comprehensive Assessment of in Vivo Lumbar Spine Intervertebral Discs Using a 3d Adiabatic T<Inf>1rho</Inf> Prepared Ultrashort Echo Time (Ute-Adiab-T<Inf>1rho</Inf>) Pulse Sequence. *Quantitative Imaging in Medicine and Surgery*. January 2022;12(1):269-280. doi:https://dx.doi.org/10.21037/qims-21-308

488. Wu J, Liu YY, Jin HJ, Wang Z, Liu MY, Liu P. Fate of the Intervertebral Disc and Analysis of Its Risk Factors Following High-Energy Traumatic Thoracic and Lumbar Fractures: Mri Results of Minimum Five Years after Injury. Research Support, Non-U.S. Gov't. *European Spine Journal*. 06 2022;31(6):1468-1478. doi:https://dx.doi.org/10.1007/s00586-022-07114-0

489. Yang L, Mu L, Huang K, Zhang T, Mei Z, Zeng W, et al. Abdominal Adipose Tissue Thickness Measured Using Magnetic Resonance Imaging Is Associated with Lumbar Disc Degeneration in a Chinese Patient Population. Comparative Study. *Oncotarget*. Dec 13 2016;7(50):82055-82062. doi:https://dx.doi.org/10.18632/oncotarget.13255

490. Zhang Y, Patiman, Liu B, Zhang R, Ma X, Guo H. Correlation between Intervertebral Disc Degeneration and Bone Mineral Density Difference: A Retrospective Study of Postmenopausal Women Using an Eight-Level Mri-Based Disc Degeneration Grading System. *BMC Musculoskeletal Disorders*. Sep 03 2022;23(1):833. doi:https://dx.doi.org/10.1186/s12891-022-05793-w

491. Edmondston SJ, Song S, Bricknell RV, Davies PA, Fersum K, Humphries P, et al. Mri Evaluation of Lumbar Spine Flexion and Extension in Asymptomatic Individuals. Clinical Trial. *Manual Therapy*. Aug 2000;5(3):158-64.

492. Fujiwara A, Tamai K, An HS, Kurihashi A, Lim TH, Yoshida H, et al. The Relationship between Disc Degeneration, Facet Joint Osteoarthritis, and Stability of the Degenerative Lumbar Spine. *Journal of Spinal Disorders*. 2000;13(5):444-450. doi:https://dx.doi.org/10.1097/00002517-200010000-00013

493. Fujiwara A, Tamai K, Kurihashi A, Yoshida H, Saotome K. Relationship between Morphology of Iliolumbar Ligament and Lower Lumbar Disc Degeneration. *Journal of Spinal Disorders*. Aug 1999;12(4):348-52.

494. Fujiwara A, Tamai K, Yamato M, An HS, Yoshida H, Saotome K, et al. The Relationship between Facet Joint Osteoarthritis and Disc Degeneration of the Lumbar Spine: An Mri Study. *European Spine Journal*. 1999;8(5):396-401.

495. Fukuta S, Miyamoto K, Suzuki K, Maehara H, Inoue T, Hara A, et al. Abundance of Calpain and Aggrecan-Cleavage Products of Calpain in Degenerated Human Intervertebral Discs. *Osteoarthritis & Cartilage*. Oct 2011;19(10):1254-62. doi:https://dx.doi.org/10.1016/j.joca.2011.07.010

496. Il Youp C, Si Young P, Jong Hoon P, Seung Woo S, Soon Hyuck L, Cho IY, et al. Mri Findings of Lumbar Spine Instability in Degenerative Spondylolisthesis. *Journal of Orthopaedic Surgery (10225536)*. 2017;25(2):1-5. doi:10.1177/2309499017718907

497. Murata M, Morio Y, Kuranobu K. Lumbar Disc Degeneration and Segmental Instability: A Comparison of Magnetic Resonance Images and Plain Radiographs of Patients with Low Back Pain. *Archives of Orthopaedic & Trauma Surgery*. 1994;113(6):297-301.

498. Nanjo Y, Morio Y, Nagashima H, Hagino H, Teshima R. Correlation between Bone Mineral Density and Intervertebral Disk Degeneration in Pre- and Postmenopausal Women. Comparative Study. *Journal of Bone & Mineral Metabolism*. 2003;21(1):22-7.

499. Ochia RS, Inoue N, Takatori R, Andersson GB, An HS. In Vivo Measurements of Lumbar Segmental Motion During Axial Rotation in Asymptomatic and Chronic Low Back Pain Male Subjects. Controlled Clinical Trial Research Support, N.I.H., Extramural. *Spine*. Jun 01 2007;32(13):1394-9.

500. Buirski G, Silberstein M, Buirski G, Silberstein M. The Symptomatic Lumbar Disc in Patients with Low-Back Pain. Magnetic Resonance Imaging Appearances in Both a Symptomatic and Control Population. *Spine (03622436)*. 1993;18(13):1808-1811.

501. Ishida Y, Ohmori K, Inoue H, Suzuki K. Delayed Vertebral Slip and Adjacent Disc Degeneration with an Isthmic Defect of the Fifth Lumbar Vertebra. *Journal of Bone & Joint Surgery - British Volume*. Mar 1999;81(2):240-4.

502. Omair A, Holden M, Lie BA, Reikeras O, Brox JI. Treatment Outcome of Chronic Low Back Pain and Radiographic Lumbar Disc Degeneration Are Associated with Inflammatory and Matrix Degrading Gene Variants: A Prospective Genetic Association Study. Randomized Controlled Trial Research Support, Non-U.S. Gov't. *BMC Musculoskeletal Disorders*. Mar 22 2013;14:105. doi:https://dx.doi.org/10.1186/1471-2474-14-105

503. Chen JY, Ding Y, Lv RY, Liu QY, Huang JB, Yang ZH, et al. Correlation between Mr Imaging and Discography with Provocative Concordant Pain in Patients with Low Back Pain. *Clinical Journal of Pain*. 2011;27(2):125-130. doi:10.1097/AJP.0b013e3181fb2203

504. Lim CH, Jee WH, Son BC, Kim DH, Ha KY, Park CK. Discogenic Lumbar Pain: Association with Mr Imaging and Ct Discography. *European Journal of Radiology*. Jun 2005;54(3):431-7.

505. Lei D, Rege A, Koti M, Smith FW, Wardlaw D. Painful Disc Lesion: Can Modern Biplanar Magnetic Resonance Imaging Replace Discography? Comparative Study. *Journal of Spinal Disorders & Techniques*. Aug 2008;21(6):430-5. doi:https://dx.doi.org/10.1097/BSD.0b013e318153f7e4

506. Karadimas EJ, Siddiqui M, Smith FW, Wardlaw D. Positional Mri Changes in Supine Versus Sitting Postures in Patients with Degenerative Lumbar Spine. *Journal of Spinal Disorders and Techniques*. October 2006;19(7):495-500. doi:https://dx.doi.org/10.1097/01.bsd.0000211213.98070.c2

507. Butler D, Trafimow JH, Andersson GB, Mcneill TW, Huckman MS. Discs Degenerate before Facets. *Spine (Phila Pa 1976)*. Feb 1990;15(2):111-3. doi:10.1097/00007632-199002000-00012

508. Kealey SM, Aho T, Delong D, Barboriak DP, Provenzale JM, Eastwood JD. Assessment of Apparent Diffusion Coefficient in Normal and Degenerated Intervertebral Lumbar Disks: Initial Experience. Research Support, U.S. Gov't, Non-P.H.S. *Radiology*. May 2005;235(2):569-74.

509. Kjaer P, Leboeuf-Yde C, Sorensen JS, Bendix T. An Epidemiologic Study of Mri and Low Back Pain in 13-Year-Old Children. Research Support, Non-U.S. Gov't. *Spine*. Apr 01 2005;30(7):798-806.

510. Fabiane SM, Ward KJ, Iatridis JC, Williams FMK. Does Type 2 Diabetes Mellitus Promote Intervertebral Disc Degeneration? *European Spine Journal*. 01 Sep 2016;25(9):2716-2720. doi:https://dx.doi.org/10.1007/s00586-016-4612-3

511. Livshits G, Ermakov S, Popham M, Macgregor AJ, Sambrook PN, Spector TD, et al. Evidence That Bone Mineral Density Plays a Role in Degenerative Disc Disease: The Uk Twin Spine Study. Research Support, Non-U.S. Gov't Twin Study. *Annals of the Rheumatic Diseases*. Dec 2010;69(12):2102-6. doi:https://dx.doi.org/10.1136/ard.2010.131441

512. Livshits G, Ermakov S, Popham M, Macgregor AJ, Sambrook PN, Spector TD, et al. Lumbar Disc Degeneration and Genetic Factors Are the Main Risk Factors for Low Back Pain: The Uk Twin Spine Study. Conference Abstract. *Osteoarthritis and Cartilage*. October 2010;2):S42. doi:https://dx.doi.org/10.1016/S1063-4584%2810%2960107-0

513. Maatta JH, Wadge S, Macgregor A, Karppinen J, Williams FMK. Issls Prize Winner: Vertebral Endplate (Modic) Change Is an Independent Risk Factor for Episodes of Severe and Disabling Low Back Pain. *Spine*. 01 Aug 2015;40(15):1187-1193. doi:https://dx.doi.org/10.1097/BRS.0000000000000937

514. Macgregor AJ, Andrew T, Sambrook PN, Spector TD. Structural, Psychological, and Genetic Influences on Low Back and Neck Pain: A Study of Adult Female Twins. Research Support, Non-U.S. Gov't Twin Study. *Arthritis & Rheumatism*. Apr 15 2004;51(2):160-7.

515. Mellor F, Morris A, Breen A. An in Vivo Study Exploring Correlations between Early-to-Moderate Disc Degeneration and Flexion Mobility in the Lumbar Spine. *European Spine Journal*. 01 Oct 2020;29(10):2619-2627. doi:https://dx.doi.org/10.1007/s00586-020-06526-0

516. Sambrook PN, Macgregor AJ, Spector TD. Genetic Influences on Cervical and Lumbar Disc Degeneration: A Magnetic Resonance Imaging Study in Twins. Comparative Study Research Support, Non-U.S. Gov't Twin Study. *Arthritis & Rheumatism*. Feb 1999;42(2):366-72.

517. Shambrook J, Mcnee P, Clare Harris E, Kim M, Sampson M, Palmer KT, et al. Clinical Presentation of Low Back Pain and Association with Risk Factors According to Findings on Magnetic Resonance Imaging. *Pain*. July 2011;152(7):1659-1665. doi:https://dx.doi.org/10.1016/j.pain.2011.03.011

518. Williams FM, Manek NJ, Sambrook PN, Spector TD, Macgregor AJ. Schmorl's Nodes: Common, Highly Heritable, and Related to Lumbar Disc Disease. Research Support, Non-U.S. Gov't. *Arthritis & Rheumatism*. Jun 15 2007;57(5):855-60.

519. Williams FMK, Popham M, Sambrook PN, Jones AF, Spector TD, Macgregor AJ. Progression of Lumbar Disc Degeneration over a Decade: A Heritability Study. *Annals of the Rheumatic Diseases*. July 2011;70(7):1203-1207. doi:https://dx.doi.org/10.1136/ard.2010.146001

520. Boos N, Dreier D, Hilfiker E, Schade V, Kreis R, Hora J, et al. Tissue Characterization of Symptomatic and Asymptomatic Disc Herniations by Quantitative Magnetic Resonance Imaging. Comparative Study Research Support, Non-U.S. Gov't. *Journal of Orthopaedic Research*. Jan 1997;15(1):141-9.

521. Danielsson AJ, Cederlund CG, Ekholm S, Nachemson AL. The Prevalence of Disc Aging and Back Pain after Fusion Extending into the Lower Lumbar Spine. A Matched Mr Study Twenty-Five Years after Surgery for Adolescent Idiopathic Scoliosis. Research Support, Non-U.S. Gov't. *Acta Radiologica*. Mar 2001;42(2):187-97.

522. Elfering A, Semmer N, Birkhofer D, Zanetti M, Hodler J, Boos N. Risk Factors for Lumbar Disc Degeneration: A 5-Year Prospective Mri Study in Asymptomatic Individuals. Research Support, Non-U.S. Gov't. *Spine*. Jan 15 2002;27(2):125-34.

523. Masui T, Yukawa Y, Nakamura S, Kajino G, Matsubara Y, Kato F, et al. Natural History of Patients with Lumbar Disc Herniation Observed by Magnetic Resonance Imaging for Minimum 7 Years. Comparative Study. *Journal of Spinal Disorders & Techniques*. Apr 2005;18(2):121-6.

524. Oishi Y, Shimizu K, Katoh T, Nakao H, Yamaura M, Furuko T, et al. Lack of Association between Lumbar Disc Degeneration and Osteophyte Formation in Elderly Japanese Women with Back Pain. Research Support, Non-U.S. Gov't. *Bone*. Apr 2003;32(4):405-11.

525. Weishaupt D, Zanetti M, Hodler J, Min K, Fuchs B, Pfirrmann CWA, et al. Painful Lumbar Disk Derangement: Relevance of Endplate Abnormalities at Mr Imaging. *Radiology*. 2001;218(2):420-427. doi:http://dx.doi.org/10.1148/radiology.218.2.r01fe15420

526. Battie MC, Videman T, Levalahti E, Gill K, Kaprio J. Heritability of Low Back Pain and the Role of Disc Degeneration. Research Support, N.I.H., Extramural Research Support, Non-U.S. Gov't Twin Study. *Pain*. Oct 2007;131(3):272-280. doi:https://dx.doi.org/10.1016/j.pain.2007.01.010

527. Battie MC, Videman T, Gibbons LE, Manninen H, Gill K, Pope M, et al. Occupational Driving and Lumbar Disc Degeneration: A Case-Control Study. Research Support, Non-U.S. Gov't Research Support, U.S. Gov't, P.H.S. *Lancet*. Nov 02 2002;360(9343):1369-74.

528. Bechara BP, Agarwal V, Boardman J, Perera S, Weiner DK, Vo N, et al. Correlation of Pain with Objective Quantification of Magnetic Resonance Images in Older Adults with Chronic Low Back Pain. Research Support, N.I.H., Extramural Research Support, Non-U.S. Gov't. *Spine*. Mar 15 2014;39(6):469-75. doi:https://dx.doi.org/10.1097/BRS.0000000000000181

529. Mariconda M, Galasso O, Imbimbo L, Lotti G, Milano C. Relationship between Alterations of the Lumbar Spine, Visualized with Magnetic Resonance Imaging, and Occupational Variables. *European Spine Journal*. Feb 2007;16(2):255-66.

530. Burke SM, Hwang SW, Mehan WA, Jr., Bedi HS, Ogbuji R, Riesenburger RI. Reliability of the Modified Tufts Lumbar Degenerative Disc Classification between Neurosurgeons and Neuroradiologists. *Journal of Clinical Neuroscience*. Jul 2016;29:111-6. doi:https://dx.doi.org/10.1016/j.jocn.2015.10.040

531. Riesenburger RI, Safain MG, Ogbuji R, Hayes J, Hwang SW. A Novel Classification System of Lumbar Disc Degeneration. *Journal of Clinical Neuroscience*. Feb 2015;22(2):346-51. doi:https://dx.doi.org/10.1016/j.jocn.2014.05.052

532. Battie MC, Levalahti E, Videman T, Burton K, Kaprio J. Heritability of Lumbar Flexibility and the Role of Disc Degeneration and Body Weight. Research Support, N.I.H., Extramural Research Support, Non-U.S. Gov't Twin Study. *Journal of Applied Physiology*. Feb 2008;104(2):379-85.

533. Djurasovic M, Carreon LY, Crawford CH, 3rd, Zook JD, Bratcher KR, Glassman SD. The Influence of Preoperative Mri Findings on Lumbar Fusion Clinical Outcomes. *European Spine Journal*. Aug 2012;21(8):1616-23. doi:https://dx.doi.org/10.1007/s00586-012-2244-9

534. Frobin W, Brinckmann P, Kramer M, Hartwig E. Height of Lumbar Discs Measured from Radiographs Compared with Degeneration and Height Classified from Mr Images. Clinical Trial Comparative Study Randomized Controlled Trial. *European Radiology*. 2001;11(2):263-9.

535. Kilitci A, Asan Z, Yuceer A, Aykanat O, Durna F. Comparison of the Histopathological Differences between the Spinal Material and Posterior Longitudinal Ligament in Patients with Lumbar Disc Herniation: A Focus on the Etiopathogenesis. *Annals of Saudi Medicine*. Mar-Apr 2021;41(2):115-120. doi:https://dx.doi.org/10.5144/0256-4947.2021.115

536. Luoma K, Vehmas T, Raininko R, Luukkonen R, Riihimaki H. Lumbosacral Transitional Vertebra: Relation to Disc Degeneration and Low Back Pain. Research Support, Non-U.S. Gov't. *Spine*. Jan 15 2004;29(2):200-5.

537. Thalgott JS, Albert TJ, Vaccaro AR, Aprill CN, Giuffre JM, Drake JS, et al. A New Classification System for Degenerative Disc Disease of the Lumbar Spine Based on Magnetic Resonance Imaging, Provocative Discography, Plain Radiographs and Anatomic Considerations. Review. *Spine Journal: Official Journal of the North American Spine Society*. Nov-Dec 2004;4(6 Suppl):167S-172S.

538. Videman T, Battie MC, Gibbons LE, Kaprio J, Koskenvuo M, Kannus P, et al. Disc Degeneration and Bone Density in Monozygotic Twins Discordant for Insulin-Dependent Diabetes Mellitus. Research Support, Non-U.S. Gov't Research Support, U.S. Gov't, P.H.S. Twin Study. *Journal of Orthopaedic Research*. Sep 2000;18(5):768-72.

539. Videman T, Battié MC, Ripatti S, Gill K, Manninen H, Kaprio J, et al. Determinants of the Progression in Lumbar Degeneration: A 5-Year Follow-up Study of Adult Male Monozygotic Twins. *Spine (03622436)*. 2006;31(6):671-678. doi:10.1097/01.brs.0000202558.86309.ea

540. Jiang X, Chen D, Li Z, Lou Y. Correlation between Lumbar Spine Facet Joint Orientation and Intervertebral Disk Degeneration: A Positional Mri Analysis. *Journal of Neurological Surgery*. Jul 2019;80(4):255-261. doi:https://dx.doi.org/10.1055/s-0039-1683450

541. Videman T, Levalahti E, Battie MC. The Effects of Anthropometrics, Lifting Strength, and Physical Activities in Disc Degeneration. Research Support, N.I.H., Extramural Research Support, Non-U.S. Gov't Twin Study. *Spine*. Jun 01 2007;32(13):1406-13.

542. Videman T, Gibbons LE, Kaprio J, Battie MC. Challenging the Cumulative Injury Model: Positive Effects of Greater Body Mass on Disc Degeneration. Research Support, N.I.H., Extramural Research Support, Non-U.S. Gov't Twin Study. *Spine Journal: Official Journal of the North American Spine Society*. Jan 2010;10(1):26-31. doi:https://dx.doi.org/10.1016/j.spinee.2009.10.005

543. Videman T, Gibbons LE, Battie MC. Age-and Pathology-Specific Measures of Disc Degeneration. *Spine (Philadelphia, Pa. 1976)*. 2008;33(25):2781-2788. doi:10.1097/brs.0b013e31817e1d11

544. Paajanen H, Haapasalo H, Kotilainen E, Aunapuu M, Kettunen J. Proliferation Potential of Human Lumbar Disc after Herniation. *Journal of Spinal Disorders*. Feb 1999;12(1):57-60.

545. Saaksjarvi S, Kerttula L, Luoma K, Paajanen H, Waris E. Disc Degeneration of Young Low Back Pain Patients: A Prospective 30-Year Follow-up Mri Study. *Spine*. Oct 01 2020;45(19):1341-1347. doi:https://dx.doi.org/10.1097/BRS.0000000000003548

546. Tertti MO, Salminen JJ, Paajanen HE, Terho PH, Kormano MJ. Low-Back Pain and Disk Degeneration in Children: A Case-Control Mr Imaging Study. Research Support, Non-U.S. Gov't. *Radiology*. Aug 1991;180(2):503-7.

547. Waris E, Eskelin M, Hermunen H, Kiviluoto O, Paajanen H. Disc Degeneration in Low Back Pain: A 17-Year Follow-up Study Using Magnetic Resonance Imaging. *Spine*. Mar 15 2007;32(6):681-4.

548. Hancock MJ, Battie MC, Videman T, Gibbons L. The Role of Back Injury or Trauma in Lumbar Disc Degeneration: An Exposure-Discordant Twin Study. Comparative Study Research Support, N.I.H., Extramural Research Support, Non-U.S. Gov't Twin Study. *Spine*. Oct 01 2010;35(21):1925-9. doi:https://dx.doi.org/10.1097/BRS.0b013e3181d60598

549. Luoma K, Vehmas T, Riihimäki H, Raininko R, Luoma K, Vehmas T, et al. Disc Height and Signal Intensity of the Nucleus Pulposus on Magnetic Resonance Imaging as Indicators of Lumbar Disc Degeneration. *Spine (03622436)*. 2001;26(6):680-686.

550. Nagashima M, Abe H, Amaya K, Matsumoto H, Yanaihara H, Nishiwaki Y, et al. Risk Factors for Lumbar Disc Degeneration in High School American Football Players: A Prospective 2-Year Follow-up Study. *American Journal of Sports Medicine*. Sep 2013;41(9):2059-64. doi:https://dx.doi.org/10.1177/0363546513495173

551. Ding WY, Yang DL, Cao LZ, Sun YP, Zhang W, Xu JX, et al. Intervertebral Disc Degeneration and Bone Density in Degenerative Lumbar Scoliosis: A Comparative Study between Patients with Degenerative Lumbar Scoliosis and Patients with Lumbar Stenosis. *Chinese Medical Journal*. 2011;124(23):3875-3878. doi:https://dx.doi.org/10.3760/cma.j.issn.0366-6999.2011.23.008

552. Harada A, Okuizumi H, Miyagi N, Genda E. Correlation between Bone Mineral Density and Intervertebral Disc Degeneration. Research Support, Non-U.S. Gov't. *Spine*. Apr 15 1998;23(8):857-61; discussion 862.

553. Su Y, Ren D, Chen Y, Geng L, Yao S, Wu H, et al. Effect of Endplate Reduction on Endplate Healing Morphology and Intervertebral Disc Degeneration in Patients with Thoracolumbar Vertebral Fracture. *European Spine Journal*. 01 2023;32(1):55-67. doi:https://dx.doi.org/10.1007/s00586-022-07215-w

554. Hu X, Chen M, Pan J, Liang L, Wang Y. Is It Appropriate to Measure Age-Related Lumbar Disc Degeneration on the Mid-Sagittal Mr Image? A Quantitative Image Study. Research Support, Non-U.S. Gov't. *European Spine Journal*. 05 2018;27(5):1073-1081. doi:https://dx.doi.org/10.1007/s00586-017-5357-3

555. Feng Z, Liu Y, Wei W, Hu S, Wang Y. Type Ii Modic Changes May Not Always Represent Fat Degeneration: A Study Using Mr Fat Suppression Sequence. *Spine*. Aug 15 2016;41(16):E987-E994. doi:https://dx.doi.org/10.1097/BRS.0000000000001526

556. Feng Z, Liu Y, Yang G, Battie MC, Wang Y. Lumbar Vertebral Endplate Defects on Magnetic Resonance Images: Classification, Distribution Patterns, and Associations with Modic Changes and Disc Degeneration. *Spine*. 07 01 2018;43(13):919-927. doi:https://dx.doi.org/10.1097/BRS.0000000000002450

557. Lv B, Yuan J, Ding H, Wan B, Jiang Q, Luo Y, et al. Relationship between Endplate Defects, Modic Change, Disc Degeneration, and Facet Joint Degeneration in Patients with Low Back Pain. *BioMed Research International*. 2019;2019:9369853. doi:https://dx.doi.org/10.1155/2019/9369853

558. Lu X, Zhu Z, Pan J, Feng Z, Lv X, Battie MC, et al. Traumatic Vertebra and Endplate Fractures Promote Adjacent Disc Degeneration: Evidence from a Clinical Mr Follow-up Study. *Skeletal Radiology*. May 2022;51(5):1017-1026. doi:https://dx.doi.org/10.1007/s00256-021-03846-0

559. Oktay AB, Albayrak NB, Akgul YS. Computer Aided Diagnosis of Degenerative Intervertebral Disc Diseases from Lumbar Mr Images. *Computerized Medical Imaging & Graphics*. Oct 2014;38(7):613-9. doi:https://dx.doi.org/10.1016/j.compmedimag.2014.04.006

560. Byvaltsev VA, Stepanov IA, Kalinin AA, Belykh EG. Quantitative Assessment of the Degree of Degenerative Change in Intervertebral Disks Using Diffusion-Weighted Images. *Biomedical Engineering*. 01 Nov 2017;51(4):275-279. doi:https://dx.doi.org/10.1007/s10527-017-9730-7

561. Cavusoglu M, Pazahr S, Ciritsis AP, Rossi C. Quantitative <Sup>23</Sup> Na-Mri of the Intervertebral Disk at 3 T. Research Support, Non-U.S. Gov't. *NMR in Biomedicine*. 08 2022;35(8):e4733. doi:https://dx.doi.org/10.1002/nbm.4733

562. Meadows KD, Johnson CL, Peloquin JM, Spencer RG, Vresilovic EJ, Elliott DM. Impact of Pulse Sequence, Analysis Method, and Signal to Noise Ratio on the Accuracy of Intervertebral Disc T <Sub>2</Sub> Measurement. *JOR Spine*. Sep 2020;3(3):e1102. doi:https://dx.doi.org/10.1002/jsp2.1102

563. Shen S, Wang H, Shi CZ, Guan SY, Liu SR. Mr T2<Sup>*</Sup>Mapping in Lumbar Intervertebral Discs of Young Volunteers. [Chinese]. *Chinese Journal of Medical Imaging Technology*. November 2010;26(11):2164-2167.

564. Vaga S, Raimondi MT, Caiani EG, Costa F, Giordano C, Perona F, et al. Quantitative Assessment of Intervertebral Disc Glycosaminoglycan Distribution by Gadolinium-Enhanced Mri in Orthopedic Patients. *Magnetic Resonance in Medicine*. Jan 2008;59(1):85-95.

565. Park JB, Chang H, Kim KW, Park SJ. Facet Tropism: A Comparison between Far Lateral and Posterolateral Lumbar Disc Herniations. Comparative Study. *Spine*. Mar 15 2001;26(6):677-9.

566. Bajpai J, Saini S, Singh R. Clinical Correlation of Magnetic Resonance Imaging with Symptom Complex in Prolapsed Intervertebral Disc Disease: A Cross-Sectional Double Blind Analysis. *Journal of Craniovertebral Junction & Spine*. Jan 2013;4(1):16-20. doi:https://dx.doi.org/10.4103/0974-8237.121619

567. Manav V, Ilhan D, Mercan H, Kilic A, Polat AK, Aksu AEK. Association between Intervertebral Disc Degeneration and Behcet's Disease. *Dermatologic Therapy*. 07 2022;35(7):e15585. doi:https://dx.doi.org/10.1111/dth.15585

568. Sivas FA, Ciliz D, Erel U, Inal EE, Özoran K, Sakman B. Abnormal Lumbar Magnetic Resonance Imaging in Asymptomatic Individuals. *Turkish Journal of Physical Medicine & Rehabilitation / Turkiye Fiziksel Tip ve Rehabilitasyon Dergisi*. 2009;55(2):73-77.

569. Hupli M, Heinonen R, Vanharanta H. Height Changes among Chronic Low Back Pain Patients During Intense Physical Exercise. Research Support, Non-U.S. Gov't. *Scandinavian Journal of Medicine & Science in Sports*. Feb 1997;7(1):32-7.
