# Supplementary material for: MRI‐Based Grading Systems for Assessing Lumbar Disc Degeneration: A Scoping Review
Source: JOR Spine. 2025 Sep 15;8(3):e70113. doi: 10.1002/jsp2.70113 (PMC12435304; doi:10.1002/jsp2.70113)
Supplement: Supplementary file 3 — Data S3: Supporting Information. [file JSP2-8-e70113-s002.docx]

**Online Resource 3.**

Subjective grading systems for lumbar degenerative disc disease on MRI

*MRI-based grading systems that used disc signal intensity alone in the assessment of degenerative disc disease in the lumbar spine*

| **Grading components** | **Grade** | **Description (visual brightness of the disc)** |
| --- | --- | --- |
| Visual brightness of the disc | 1 | Normal |
|  | 2 | Mild loss* |
|  | 3 | Moderate loss* |
|  | 4 | Severe |

**Table 1.** Grading system for lumbar disc degeneration proposed by Decandido [1]

*Mild and moderate loss were described as intermediate signal intensities between the two extremes

| **Grading components** | **Description** |
| --- | --- |
| Disc signal intensity | Any form of reduction was considered disc degeneration |

**Table 2.** Grading system for lumbar disc degeneration proposed by Dimar [2]

| **Grading components** | **Grade** | **Description** |
| --- | --- | --- |
| Disc signal intensity | Normal | A normal disc signal was described as high or bright signal |
|  | Abnormal | A decreased, black, or gray signal was interpreted as evidence of degeneration or dehydration |

**Table 3.** Grading system for lumbar disc degeneration proposed by Evans [3]

| **Grading components** | **Grade** | **Description** |
| --- | --- | --- |
| Disc signal intensity | 0 | Homogenously hyperintense |
|  | 1 | Hyperintense with visible intranuclear cleft |
|  | 2 | Intermediate signal intensity |
|  | 3 | Hypointense |

**Table 4.** Grading system for lumbar disc degeneration as reported in Dragsbaek [4]

Proposed by Eyre [5]

| **Grading Components** | **Grade** | **Description** |
| --- | --- | --- |
| Disc hydration | 0 | Normal |
|  | 1 | Partially reduced |
|  | 2 | Completely black disc |

**Table 5.** Grading system for lumbar disc degeneration proposed by Fu [6]

| **Grade** | **Description*** |
| --- | --- |
| 0 | Pure, hyperintense signal (normal) |
| 1 | Early degeneration |
| 2 | Moderate degeneration |
| 3 | Severe degeneration |
| 4 | Total loss of nuclear |
|  | signal/hypointense (dark) |

**Table 6.** Grading system for lumbar disc degeneration proposed by Gibson [7]

*Degeneration was graded according to a 5-point scale, ranging from 0 = normal to 4 = total loss of nuclear signal.

| **Grading Component** | **Description** |
| --- | --- |
| Disc dehydration | Presence of lumbar degenerative disc disease as manifested by greater than 50% degenerative disc dehydration compared with normal discs |

**Table 7.** Grading system for lumbar disc degeneration proposed by Heithoff [8]

| **Grading components** | **Description** |
| --- | --- |
| Nuclear signal intensity | Normal |
|  | Moderate loss |
|  | Severe |

**Table 8.** Grading system for lumbar disc degeneration proposed by Ito [9]

| **Grading components** | **Grade** | **Description** |
| --- | --- | --- |
| Visual brightness of the discs on a T2-weighted image in comparison to the signal intensity of the lumbar vertebrae | No degeneration | Not specified |
|  | Mild | Not specified |
|  | Severe | Not specified |

**Table 9.** Grading system for lumbar disc degeneration proposed by Kotilainen [10]

| **Grading components** | **Grade** | **Description** |
| --- | --- | --- |
| Disc signal intensity* | Mild, moderate, or marked | Mild, moderate, and marked decrease in signal intensity were classified as being abnormal with no differentiation as to the degree of abnormality |

**Table 10.** Grading system for lumbar disc degeneration proposed by Linson [11]

***Signal intensity was compared with the adjacent disc spaces in the same patient

| **Grading components** | **Grade** | **Description** |
| --- | --- | --- |
| Disc signal intensity | Bright | Discs with a nucleus pulposus brighter or as bright as CSF were classified as having normal intensity |
|  | Dark | Discs with a nucleus pulposus darker than CSF were classified as having decreased signal intensity |

**Table 11.** Grading system for lumbar disc degeneration proposed by Liuke [12]

| **Grading components** | **Grade** | **Description** |
| --- | --- | --- |
| Signal intensity of the nucleus pulposus* | 1 | Bright |
|  | 2 | Grey |
|  | 3 | Dark |
|  | 4 | Black |

**Table 12.** Grading system for lumbar disc degeneration proposed by Luoma [13]

*Signal intensity was visually estimated using CSF in the adjacent dural sac as an intensity reference

| **Grading components** | **Grade** | **Description** |
| --- | --- | --- |
| Disc signal intensity | Bright (1) | High signal intensity appearance (bright) normal |
|  | Gray (2) | Intermediate intensity appearance for early degenerative change |
|  | Dark (3) | Low signal appearance for well-established degenerative change |

**Table 13.** Grading system for lumbar disc degeneration as reported in Madan [14]

Proposed by Marchiori [15].

| **Grading components** | **Description** |
| --- | --- |
| Disc signal intensity | Disc degeneration/desiccation was diagnosed when there was a decrease in disc signal intensity on T2-weighted images |

**Table 14.** Grading system for lumbar disc degeneration proposed by Maurer [16]

| **Grading Components** | **Grade** | **Description** |
| --- | --- | --- |
| Disc signal intensity | Healthy/well hydrated | High signal intensity |
|  | Abnormal/degenerated | Over 50% decrease of MR signal intensity was detected when compared to the maximal signal intensity of the lumbar discs |
|  |  |  |

**Table 15.** Grading system for lumbar disc degeneration proposed by Tertti [17]

*Grading systems that used disc height alone in the assessment of degenerative disc disease*

| **Grading Components** | **Grade** | **Description** |  |
| --- | --- | --- | --- |
| Disc space height | 0 | Normal |  |
|  | 1 | Mild, reduced <50% |  |
|  | 2 | Moderate/severe, reduced ≥50% |  |

**Table 16.** Grading system for lumbar disc degeneration proposed by Fu [6]

| **Grading components** | **Description** |
| --- | --- |
| Disc narrowing | Normal |
|  | Moderate narrowing |
|  | Severe narrowing |

**Table 17.** Grading system for lumbar disc degeneration proposed by Ito [9]

| **Grading components** | **Grade** | **Description** |
| --- | --- | --- |
| Disc height | 0 | Disc higher than the disc above |
|  | 1 | Disc as high as the disc above (if normal) |
|  | 2 | Disc narrower than the disc above (if normal) |
|  | 3 | Endplates almost in contact |

**Table 18.** Grading system for lumbar disc degeneration proposed by Raininko [18]

| **Grading components** | **Grade** | **Description** |
| --- | --- | --- |
| Disc height | 0-4 | Disc height narrowing was determined from qualitative evaluations of films using a 4-point scale which was not specified |

**Table 19.** Grading system for lumbar disc degeneration proposed by Videman [19]

*Grading systems that used disc signal intensity and disc height in the assessment of degenerative disc disease*

| **Grade** | **Description** |
| --- | --- |
| 0 | Normal |
| 1 | Mild (slight dehydration of the disc on T2-weighted images) |
| 2 | Moderate (disc dehydration and mild loss of disc height |
| 3 | Severe (total disc dehydration with nearly complete loss of disc height) |

**Table 20.** Grading system for lumbar disc degeneration proposed by Borenstein [20]

| **Grading components** | **Description** |
| --- | --- |
| Disc dehydration | Description not specified |
| Disc narrowing | Description not specified |

**Table 21.** Grading system for lumbar disc degeneration proposed by Butterman [21]

| **Grading components** | **Grade** | **Description** |
| --- | --- | --- |
| Disc signal intensity | 1 | Hyper-intense with visible intra-nuclear cleft |
|  | 2 | Intermediate signal intensity |
|  | 3 | Hypo-intense |
| Disc height* | 1 | Disc higher than the one above |
|  | 2 | Disc as high as the disc above (if normal) |
|  | 3 | Disc narrower than the disc above (if normal) |
|  | 4 | Endplates almost in contact |

**Table 22.** Grading system for lumbar disc degeneration proposed by Jensen [22]

Used in a latent class analysis. Intervertebral disc was categorised as being degenerated if its disc signal intensity was grade 3, or its disc height was graded as 3 or 4 *Disc height measured using the system by Raininko [18]

| **Grading component** | **Description** |
| --- | --- |
| Signal intensity | Considered as any loss of signal intensity of the disc |
| Disc height | Considered as any disc height loss of the disc |

**Table 23.** Grading system for lumbar disc degeneration proposed by Lakadamyali [23]

| **Grading components** | **Description** |
| --- | --- |
| Disc height | Grade 2 or 3 |
| Disc signal | Grade 3 |

**Table 24.** Grading system for lumbar disc degeneration proposed by Leboeuf-Yde [24] Disc degeneration was defined as either reduced disc height or signal intensity

| **Grading components** | **Grade** | **Description** |
| --- | --- | --- |
| Signal intensity | 0-5 | If the signal intensity of both nucleus pulposus and annulus fibrosis was very dark like that of cortical bone, the disc signal intensity was classified as severely decreased. If signal intensity in nucleus pulposus was bright (normal or increased signal) but that of annulus very dark in a disc with a decreased height, the disc signal intensity was classified as increased |
| Disc height | 0-4 | Disc height (anterior, posterior, and middle) was visually estimated as normal (higher than, or as high as the upper not degenerated disc space), slightly decreased (<33% lower than the upper disc space), clearly decreased (34–66% lower), or strongly decreased (>66% or lower) |

**Table 25.** Grading system for lumbar disc degeneration proposed by Luoma [25]

| **Grading components** | **Grade** | **Description** |
| --- | --- | --- |
| Disc signal intensity and disc height | 0 | Normal disc with a bright homogeneous centre and disc height preserved |
|  | 1 | Mildly inhomogeneous disc but disc height preserved |
|  | 2 | Mildly homogeneous disc with a disc height loss of <50% |
|  | 3 | Black disc with a disc height loss of <50% |
|  | 4 | Black disc with a disc height loss of >50% |

**Table 26.** Grading system for lumbar disc degeneration proposed by Sabnis [26]

Each disc was assigned weight per the following protocols; (i) No points, if the disc was normal (bright homogenous centre and normal disc height compared with adjacent level discs); (ii) One point when the disc was inhomogeneous (but not entirely black), and two points when the disc was entirely black; (iii) One point for a disc height loss of <50% (compared with the cephalad disc height), and two points for a disc height loss of >50%. Points were added to give an overall grade (0-4) to each disc

**Table 27.** Grading system for lumbar disc degeneration proposed by Schneidermann [27]

| **Grade** | **Description** |
| --- | --- |
| Normal | No signal changes |
| 1 | Slight decrease in signal intensity of the nucleus pulposis |
| 2 | Hypointense nucleus pulposis with normal disc height |
| 3 | Hypointense nucleus pulposus with disc space narrowing |

| **Grading components** | **Grade** | **Description** |
| --- | --- | --- |
| Disc signal intensity and disc height | Mild | A decrease in signal intensity of the nucleus pulposus on T2-weighted images |
|  | Moderate | Hypo-intense nucleus pulposus on T2-weighted images |
|  | Severe | Hypointense nucleus pulposus with narrowing of the disc space |

**Table 28.** Grading system for lumbar disc degeneration as reported in Karppinen [28]

Proposed by Stadnik [29]

| **Grading components** | **Grade** | **Description** |
| --- | --- | --- |
| Hydration status and disc height | Normal | The hydration status and height of the intervertebral disc was evaluated. Discs with normal hydration and height were considered normal |
|  | Degenerated | Endplates were considered degenerated if there was either a significant increase or decrease in signal intensities. If either the discs or the endplates were diagnosed as degenerated, the disc was classified as degenerated |

**Table 29.** Grading system for lumbar disc degeneration proposed by Throckmorton [30]

*MRI-based grading systems that used any combination of disc signal intensity and bulge, disc height and bulge, and disc signal intensity, disc height and bulge in the assessment of degenerative disc disease in the lumbar spine*

| **Grading components** | **Grade** | **Description** |
| --- | --- | --- |
| Disc height | 0 | Normal |
|  | 1 | Mild* |
|  | 2 | Moderate* |
|  | 3 | Severe* |
| Disc Bulging | 0 | Normal |
|  | 1 | Mild |
|  | 2 | Moderate |
|  | 3 | Severe |

**Table 30.** Grading system for lumbar disc degeneration proposed by Battie [31]

*Mild, moderate, and severe were described as progressive degrees of abnormality.

| **Grading components** | **Grade** | **Description** |
| --- | --- | --- |
| Disc signal intensity | 0 | Normal |
|  | 1 | Mild* |
|  | 2 | Moderate* |
|  | 3 | Severe* |
| Disc height narrowing | 0 | Normal |
|  | 1 | Mild |
|  | 2 | Moderate |
|  | 3 | Severe |
| Disc Bulging | 0 | Normal |
|  | 1 | Mild |
|  | 2 | Moderate |
|  | 3 | Severe |

**Table 31.** Grading system for lumbar disc degeneration proposed by Battie [32]

*Mild, moderate, and severe were described as progressive degrees of abnormality.

| **Grading components** | **Description** |
| --- | --- |
| Disc signal intensity | Low |
| Herniation | Present |

**Table 32.** Grading system for lumbar disc degeneration proposed by Deng [33]

| **Grading components** | **Grade** | **Description** |
| --- | --- | --- |
| MRI scoring for disc appearance | 0 | Normal appearance |
|  | 1 | Some loss of signal but disc structure still visible |
|  | 2 | Significant signal loss with loss of structure but no loss of disc height or loss of disc height with normal structure |
|  | 3 | Loss of disc height and signal |
|  | 4 | Loss of disc height and signal with annular disruption |
| MRI scoring for disc protrusion | 0 | Normal |
|  | 1 | Annular disc bulge |
|  | 2 | Focal disc protrusion |
|  | 3 | Disc extrusion |

**Table 33.** Grading system for lumbar disc degeneration proposed by Desigan [34]

| **Grading description** | **Grade** | **Description** |
| --- | --- | --- |
| Disc herniation and signal intensity changes* | 0 | No sign of disc degeneration or herniation |
|  | 1 | Loss of water content and/or disc height |
|  | 2 | Disc protrusion |
|  | 3 | Disc extrusion |

**Table 34.** Grading system for lumbar disc degeneration proposed by Fardon [35]

As reported in Kiil [36]

| **Grading components** | **Grade** | **Description** |
| --- | --- | --- |
| Nuclear intensity | White | Homogenous, hypointense signal was defined |
|  | Speckled | A speckled pattern consisted of dark signal and two or more areas of light signal |
| Bulge | Flat | A straight or minimally convex posterior annulus |
|  | Bulged | A convex annulus that encroached the anterior thecal sac |
|  | Torn | Definite discontinuity in the signal of the posterior annulus or posterior longitudinal ligament |

**Table 35.** Grading system for lumbar disc degeneration proposed by Horton and Daftari [37]

When the two grading components were combined, each disc could be classified into nine possible patterns

| **Grade** | **I** | **II** | **III** | **IV** |
| --- | --- | --- | --- | --- |
| Signal intensity | High | High-moderate | Moderate-low | Absent |
| Disc bulging | Normal | Rupture of the posterior annulus | Protrusion or extrusions of the disc materials | |

**Table 36.** Grading system for lumbar disc degeneration proposed by Kanamori [38]

| **Quantitative components** | **Description** |
| --- | --- |
| Disc signal intensity | Cerebrospinal fluid at the corresponding disc level was used as a signal intensity reference. Intensity lower than that of the adjacent cerebrospinal fluid was considered a positive finding and was called a dark nucleus pulposus |
| Disc height | Graded on a 4-point scale (0 = normal, 1 = slightly decreased, 2 = distinctly decreased, and 3 = severely decreased) |
| Disc bulging | Bulge anterior or posteriorly |

**Table 37.** Grading system for lumbar disc degeneration proposed by Solovieva [39]

| **Grading components** | **Grade** | **Description** |
| --- | --- | --- |
| Disc height narrowing | 0 | Normal- typically disc higher than the upper disc |
|  | 1 | Slight- disc as high as the upper disc if it is normal |
|  | 2 | Moderate- disc narrower than the upper disc if it is normal |
|  | 3 | Severe- endplates almost in contact |
| Disc bulging | 0 | None- normal contour of the disc |
|  | 1 | Slight- approximately 1.51 ± 1mm |
|  | 2 | Moderate- approximately 3.5 ± 1mm |
|  | 3 | Severe- ≥4.5 mm |

**Table 38.** Grading system for lumbar disc degeneration proposed by Videman [40]

| **Grading components** | **Grade** | **Description** |
| --- | --- | --- |
| Disc height | 0-3 | 0 equaling normal and 1 through 3 representing progressive degrees of abnormality |
| Disc bulging (anteriorly and posteriorly) | 0-3 | 0 equaling normal and 1 through 3 representing progressive degrees of abnormality (if bulging was detected both anteriorly and posteriorly, the larger of the ratings was used |
| Signal Intensity | 0-3 | 0 equaling normal and 1 through 3 representing progressive degrees of abnormality |

**Table 39.** Grading system for lumbar disc degeneration proposed by Videman [41]

| **Grading components** | **Grade** | **Description** |
| --- | --- | --- |
| Disc signal reduction/ degeneration | 0 | Normal |
|  | 1 | Slight reduction as compared to normal appearing adjacent discs |
|  | 2 | Moderate reduction |
|  | 3 | Severe reduction (complete or near complete lack of signal) |
| Disc height | 0 | Normal |
|  | 1 | Reduction ≤ 50% |
|  | 2 | Reduction 50%-90% |
|  | 3 | Reduction > 90% |
| Disc bulging | 0 | Normal |
|  | 1 | Bulging disc |

**Table 40.** Grading system for lumbar disc degeneration proposed by WitWit [42].

Disc degeneration as a separate category was defined as a combination and/or either of reduced disc signal, reduced disc height and disc bulging

*MRI-based grading systems that used disc signal intensity and/or disc height, herniation, structural changes of the disc and the distinction between the annulus fibrosis and nucleus pulposus in the assessment of disc degeneration in the lumbar spine*

| **Buirski pattern** | **Description** |
| --- | --- |
| 1 | Thickened cleft with no prolapse/bulge, and normal disc intensity and disc height |
| 2 | Thickened cleft with no prolapse/bulge, reduced disc intensity and normal disc height |
| 3 | Normal cleft, disc intensity and disc height, with prolapse/bulge |
| 4 | Thickened cleft, prolapsed/bulge, and reduced disc intensity and disc height |
| 5 | Thickened or incomplete cleft, with prolapse/bulge with focal signal voids disc intensity and moderately reduced disc |
| 6 | Cleft not visible, with prolapse/bulge, and significant disc signal intensity and severe disc height |

**Table 41.** Grading system for lumbar disc degeneration proposed by Buirski [43]

| **Grading components** | **Grade** | **Description** |
| --- | --- | --- |
| Nuclear intensity, disc height, distinction between the annulus fibrosis and nucleus pulposus and herniation | Normal | Well-preserved disc space without evidence of collapse, Smooth borders of both annulus and nucleus pulposus, no evidence of disc herniation, and a clear white signal of the disc on the T2-weighted image |
|  | Degenerated | Discs not fulfilling this criterion were considered degenerated |

**Table 42.** Grading system for lumbar disc degeneration proposed by Butler [44]

| **Grade** | **Signal From Nucleus and Inner Fibers of Annulus** | **Distinction Between Inner and Outer Fibers of Annulus at Posterior Aspect of Disc** | **Height of Disc** |
| --- | --- | --- | --- |
| 1 | Uniformly hyperintense, equal to CSF | Distinct | Normal |
| 2 | Hyperintense (>presacral fat and <CSF) ± Hypointense intranuclear cleft | Distinct | Normal |
| 3 | Hyperintense though < Presacral fat | Distinct | Normal |
| 4 | Mildly hyperintense (slightly>outer fivers of annulus) | Indistinct | Normal |
| 5 | Hypointense (= outer fivers of annulus) | Indistinct | Normal |
| 6 | Hypointense | Indistinct | <30% reduction in disc height |
| 7 | Hypointense | Indistinct | 30%-60% reduction in disc height |
| 8 | Hypointense | Indistinct | >60% reduction in disc height |

**Table 43.** Grading system for lumbar disc degeneration proposed by Griffith [45]

More commonly referred to as the modified Pfirrmann

| **Grading components** | **Description** |
| --- | --- |
| Loss of disc height  Reduction in signal intensity on a T2-weighted image  Loss of distinctness of the intranuclear cleft | Discs with only one of these findings were defined as mildly degenerated, while those with at least two findings were defined as severely degenerated |

**Table 44.** Grading system for lumbar disc degeneration proposed by Kealey [46]

| **Grading components** | **Grade** | **Description** |
| --- | --- | --- |
| Signal intensity | 0 | Homogenous hyperintense |
|  | 1 | Hyperintense with visible intranuclear cleft |
|  | 2 | Intermediate signal intensity |
|  | 3 | Hypointense |
| Nuclear shape | 0 | Round or kidney shaped, 0<60% of sagittal or coronal diameter of the disc |
|  | 1 | Slightly lobulated or irregular |
|  | 2 | Severely irregular shape and small, less than 25% of the area of the disc |
|  | 3 | Not seen in a disc of low signal intensity |
| Disc height | 0 | Disc higher than the upper disc |
|  | 1 | Disc as high as the upper disc (if normal) |
|  | 2 | Disc narrower than the upper disc (if normal) |
|  | 3 | Endplates almost in contact |

**Table 45.** Grading system for lumbar disc degeneration proposed by Kjaer [47]

| **Grade** | **Description** |
| --- | --- |
| Grade 1 | White nuclear signal, normal height bean shape nucleus, annular margins well defined, no tears |
| Grade 2 | Speckled nuclear signal, height reduced <10%, distortion of nuclear shape, small radial tears not reaching the PLL on axial views |
| Grade 3 | Speckled or dark nucleus, height reduced by 10%-50%, radial tears extending up to or torn PLL on sagittal/axial views |
| Grade 4 | Dark nucleus, height reduced by >50%, no difference between appearance of annulus and nucleus ± complex tears |

**Table 46.** Grading system for lumbar disc degeneration proposed by Lei [48].

Described as the Woodend classification

| **Grading component** | **Grade** | **Description** |
| --- | --- | --- |
| Disc signal intensity and distinction between the annulus and nucleus | I | Preserved differentiation of the nucleus pulposus from the annulus, homogeneously hyperintense signal of the nucleus pulposus |
|  | II | Preserved differentiation of the nucleus pulposus from the annulus, hyperintense signal of the nucleus pulposus with a horizontal dark band |
|  | III | Mild degeneration, blurred differentiation of the nucleus pulposus from the annulus, slightly decreased signal of the nucleus pulposus with minor irregularities |
|  | IV | Moderate degeneration, a loss of differentiation of the nucleus pulposus from the annulus, moderately decreased signal of the nucleus pulposus with hypointense zones |
|  | V | Severe degeneration, a loss of differentiation of the nucleus pulposus from the annulus, hypointense signal of the nucleus pulposus with or without horizontal hyperintense band |

**Table 47.** Grading system for lumbar disc degeneration as reported in Chen [49] and Lim [50]. Proposed by Pearce [5]

| **Grade** | **Distinction of nucleus and annulus** | **Signal intensity** | **Height of intervertebral disc** |
| --- | --- | --- | --- |
| I | Clear | Hyperintense, isointense to cerebrospinal fluid | Normal |
| II | Clear | Hyperintense, isointense to cerebrospinal fluid | Normal |
| III | Unclear | Intermediate | Normal to slightly decreased |
| IV | Lost | Intermediate to hypointense | Normal to moderately decreased |
| V | Lost | Hypointense | Collapsed disc space |

**Table 48.** Grading system for lumbar disc degeneration proposed by Pfirrmann [51]

| **Grading components** | **Grade** | **Description** |
| --- | --- | --- |
| Disc signal intensity, Disc height, and distinction between the annulus fibrosis and nucleus pulposis | 1 | Homogeneous, bright nucleus pulposus and homogenous dark gray annulus fibrosis |
|  | 2 | Horizontal dark bands extended across the annulus fibrosis |
|  | 3 | Diminished signal intensity of annulus fibrosis and nucleus pulposus indistinguishable from the annulus fibrosis |
|  | 4 | Further reduced signal intensity of the nucleus pulposus and some bright and dark signals |
|  | 5 | Diminished disc height |

**Table 49.** Grading system for lumbar disc degeneration proposed by Thompson [5]

*MRI-based grading systems that used disc signal intensity and/or disc height, in combination with osteophytes, end-plate changes, modic changes and high intensity zones in the assessment of disc degeneration in the lumbar spine*

| **Grading components** | **Grade** | **Description** |
| --- | --- | --- |
| Disc height reduction | 0 | Normal |
|  | 1 | Mild* |
|  | 2 | Moderate* |
|  | 3 | Severe* |
| Disc signal intensity | 1 | Normal |
|  | 2 | Mild* |
|  | 3 | Severe* |
| Disc bulging | 0 | Normal |
|  | 1 | Mild |
|  | 2 | Moderate |
|  | 3 | Severe |
| Anterior osteophytes | 0 | Normal |
|  | 1 | Mild |
|  | 2 | Moderate |
|  | 3 | Severe |
| Schmorl’s Nodes | 0 | Normal |
|  | 1 | Mild |
|  | 2 | Moderate |
|  | 3 | Severe |

**Table 50.** Grading system for lumbar disc degeneration proposed by Battie [52]

As reported in Videman [53].

| **Grading components** | **Grade** | **Description** |
| --- | --- | --- |
| Disc height narrowing | 0 | Normal |
|  | 1 | Mild* |
|  | 2 | Moderate* |
|  | 3 | Severe* |
| Disc Bulging | 0 | Normal |
|  | 1 | Mild |
|  | 2 | Moderate |
|  | 3 | Severe |
| Osteophytes | 0 | Normal |
|  | 1 | Mild |
|  | 2 | Moderate |
|  | 3 | Severe |

**Table 51.** Grading system for lumbar disc degeneration proposed by Battie [54]

*Mild, moderate, and severe were described as progressive degrees of abnormality

| **Grading components** | **Grade** | **Description** |
| --- | --- | --- |
| Disc height reduction | 0 | Normal |
|  | 1 | Mild* |
|  | 2 | Moderate* |
|  | 3 | Severe* |
| Disc bulging | 0 | Normal |
|  | 1 | Mild |
|  | 2 | Moderate |
|  | 3 | Severe |
| Anterior osteophytes | 0 | Normal |
|  | 1 | Mild |
|  | 2 | Moderate |
|  | 3 | Severe |
| Schmorl’s Nodes | 0 | Normal |
|  | 1 | Mild |
|  | 2 | Moderate |
|  | 3 | Severe |

**Table 52.** Grading system for lumbar disc degeneration proposed by Battie [55]

*Mild, moderate, and severe were described as progressive degrees of abnormality.

| **Grading components** | **Grade** | **Description** |
| --- | --- | --- |
| T2 Signal Intensity Loss | 0 (Healthy)- 3 (Pathologic) | Decreased signal intensity |
| Nucleus Pulposus Shape | 0 (Healthy)- 3 (Pathologic) | Abnormal shape of the nucleus pulposis |
| Modic Changes | 0 (Healthy)- 3 (Pathologic) | Abnormalities in vertebral endplates indicating degeneration |
| Osteophytes | 0 (Healthy)- 3 (Pathologic) | Formation of bone spurs, often associated with degeneration |

**Table 53.** Grading system for lumbar disc degeneration as reported in Bechara [56]

Proposed by Benneker [57].

| **Grading Components** | **Grade** | **Description** |
| --- | --- | --- |
| Disc desiccation | Present | Not specified |
|  |  |  |
| High intensity zone | Present | Area of increased T2 signal, isointense to CSF |
| Modic changes | Type I | Hypointensity on T1-weighted images and hyperintensity on T2-weighted images |
|  | Type II | Hyperintensity on T1-weighted images and isointensity or slight hyperintensity on T2-weighted images |
|  | Type III | Hypointensity on both T1 and T2-weighted images |

**Table 54.** Grading system for lumbar disc degeneration proposed by Djurasovic [58]

|  | **Nucleus signal** | **Prolapse detected** | **Bone marrow signal** |
| --- | --- | --- | --- |
| A | No signal loss | No prolapse | No intensity change |
| B | No signal loss | Prolapse | No intensity change |
| C | No signal loss | No prolapse | Intensity change |
| D | No signal loss | Prolapse | Intensity change |
| E | Moderate signal loss | No prolapse | No intensity change |
| F | Moderate signal loss | Prolapse | No intensity change |
| G | Moderate signal loss | No prolapse | Intensity change |
| H | Moderate signal loss | Prolapse | Intensity change |
| I | Total signal loss | No prolapse | No intensity change |
| J | Total signal loss | Prolapse | No intensity change |
| K | Total signal loss | No prolapse | Intensity change |
| L | Total signal loss | Prolapse | Intensity change |

**Table 55.** Grading system for lumbar disc degeneration proposed by Frobin [59]

| **Grading components** | **Grade** | **Description** |
| --- | --- | --- |
| Disc signal intensity | 0 | Normal |
|  | 1 | Mild* |
|  | 2 | Moderate* |
|  | 3 | Severe* |
| Disc height narrowing | 0 | Normal |
|  | 1 | Mild |
|  | 2 | Moderate |
|  | 3 | Severe |
| Disc bulging | 0 | Normal |
|  | 1 | Mild |
|  | 2 | Moderate |
|  | 3 | Severe |
| Anterior osteophytes | 0 | Normal |
|  | 1 | Mild |
|  | 2 | Moderate |
|  | 3 | Severe |

**Table 56.** Grading system for lumbar disc degeneration as reported in Sambrook [60] Proposed by Jarosz [61] *Mild, moderate, and severe were described as progressive degrees of abnormality

| **Grading Components** | **Grade** | **Description** |
| --- | --- | --- |
| Disc signal intensity, disc height, herniation and osteophytic change | 1 | Hyperintense signal in the nucleus, with normal disc height |
|  |  |  |
|  | 2 | Intermediate signal in the nucleus, with a slight decrease in disc height |
|  | 3 | Hypointense signal in the nucleus, with a decrease in disc height and evidence of disc herniation/osteophyte |
|  | 4 | Hypointense signal in the nucleus, with a collapsed disc height and disc herniation/osteophyte |

**Table 57.** Grading system for lumbar disc degeneration proposed by Jiang [62]

| **Radiological (MRI) parameters** | **Score 0** | **Score 1** | **Score 2** |
| --- | --- | --- | --- |
| Height loss | 0 (none) | 1 (mild/moderate) | 2 (severe) |
| Osteophyte formation | 0 (none) | 1 (mild/moderate) | 2 (severe) |
| Endplate sclerosis | 0 (none) | 1 (mild/moderate) | 3 (severe) |

**Table 58.** Grading system for lumbar disc degeneration proposed by Kilitchi [63]

| **Grading components** | | **Grade** | **Description** |
| --- | --- | --- | --- |
| Size and shape of the nuclear complex | 1 | | Regular shape, size< 60% of the sagittal diameter of the disc |
|  | 2 | | Regular shape, size >60% of the sagittal diameter of the disc |
|  | 3 | | Irregular shaggy borders, size <60% of the sagittal diameter of the disc |
|  | 4 | | Dark, no clear border with annulus fibrosis |
| Border between the outer annulus fibrosis and nuclear complex | 1 | | Smooth, concave border anteriorly and posteriorly |
|  | 2 | | Shaggy or irregular border anteriorly and/or posteriorly |
|  | 3 | | Not distinguishable anteriorly or posteriorly; dark nuclear complex |
| Homogeneity of annulus fibrosis | 1 | | Homogeneous, regular, dark lamellar structure anteriorly and posteriorly |
|  | 2 | | Inhomogeneous, irregular structure anteriorly and/or posteriorly |
|  | 3 | | No clear border to nuclear complex; dark nuclear complex |
| Homogeneity of nuclear complex | 1 | | Normal, homogeneous, bright, or light grey |
|  | 2 | | Inhomogeneous |
|  | 3 | | Homogeneous, dark |
| Regularity of the horizontal intranuclear cleft (INC) | 1 | | None |
|  | 2 | | Regular thin, grey |
|  | 3 | | Regular thick, dark |
|  | 4 | | Irregular thick, dark |
|  | 5 | | Not distinguishable; dark nuclear complex |
| Dark dot in the INC | 1 | | No dot |
|  | 2 | | Clear dot in the INC |
|  | 3 | | Not distinguishable; dark nuclear complex |
| Defect in the vertebral endplates | 1 | | No clear endplate defect |
|  | 2 | | Distinct indentation on the vertebral endplate with clear-cut edges |
|  |  | |  |

**Table 59.** Grading system for lumbar disc degeneration proposed by Luoma [64]

| **Grading components** | **Grade** | **Description** |
| --- | --- | --- |
| Disc signal intensity, morphological aspects of the nucleus pulposis, annulus fibrosis and vertebral bodies | I-V | Grade I represents a normal adolescent disc; grade II, a normal adult disc; grades III-V represent increasing degenerative changes |

**Table 60.** Grading system for lumbar disc degeneration as reported in Boos [65]

Proposed by Eyre [5]

**Table 61.** Grading system for lumbar disc degeneration proposed by Thalgott [66]

| **Classification of lumbar degenerative disc disease** |
| --- |
| **A, anterior column** |
| Normal T2-weighted signal on MRI |
| Lordotic in sagittal plane |
| Domed end plates |
| Normal density of endplates |
| No internal disc disruption/not painful |
| No herniation |
| No intersegmental motion |
| No loss of disc height |
| **B, anterior column** |
| Dehydration in T2-weighted signal on MRI, otherwise, normal anatomy |
| May have loss of lordosis in sagittal plane |
| May have slight sclerosis of endplates |
| May have internal disc disruption/may be painful |
| May have herniation |
| Slight increase in intersegmental motion |
| No loss of disc height |
| **C, anterior column** |
| Severe dehydration in T2-weighted signal on MRI nonlordotic in sagittal plane |
| May have sclerosis of endplates |
| Loss of endplate domed shape with irregularity of endplate surface |
| Internal disc disruption/painful |
| May have herniation |
| Increased intersegmental motion |
| Loss of disc height |
| **D, anterior column** |
| Severe dehydration in T2-weighted signal on MRI |
| Neutral to kyphotic in sagittal plane |
| Sclerosis of endplates |
| Total loss of end plate anatomy |
| Total internal disc disruption/painful |
| Herniation likely |
| No intersegmental motion |
| Total collapse of disc space with loss of posterior arch |
| May have anterior osteophytes |
| **E, anterior column** |
| Sagittal plane translational deformity |
| Isthmic/Lytic spondylolisthesis, Grades I-V |
| Subcategory of disc A-D |
| Motion of segment resulting from pars defect |
| Degenerative spondylolisthesis, Grades I-II |
| All have Grade C or D discs |
| May have end plate-on-end plate contact |
| **F, coronal plane deformity** |
| End plate irregularity |
| Degenerative aetiology |
| All C and D discs |
| Osteophytes |
| **Posterior column** |
| 1 No facet joint degeneration |
| 2 Facet joint degeneration/no stenosis |
| 3 Facet joint degeneration with stenosis |
| A Presence of central stenosis |
| B Presence of lateral stenosis |
| C Presence of foraminal stenosis |

| **Radiographic criteria** | **Description** | **Points assigned by original classification** | **Modified classification** |
| --- | --- | --- | --- |
| Disc structure and brightness | Presence of a distinct annulus fibrosis and nucleus; nucleus T2-weighted signal isointense to CSF | 0 | 0 |
|  | Lack of a distinction of annulus fibrosis and nucleus pulposus; nucleus pulposus T2-weighted signal completely hypointense to CSF but not completely black | 1 | 1 |
|  | Lack of a distinction of annulus fibrosis and nucleus pulposus; nucleus pulposus T2-weighted signal completely hypointense (black or dark disc) | 2 | 2 |
| Modic changes | No Type I or Type II changes | 0 | 0 |
|  | Type I or Type II changes present | 1 | 1 |
| Disc height | Greater or equal to 5mm | 0 | 0 |
|  | Less than 5 mm | 1 | 1 |
| High intensity zone | Absent | 0 | Removed |
|  | Present | 1 | Removed |

**Table 62.** Grading system for lumbar disc degeneration proposed by Tufts [67]

This includes both the original and modified system as reported in Burke [68]

| **Grading components** | **Grade** | **Description** |
| --- | --- | --- |
| Disc height | 0-3 | 0 equaling normal and 1 through 3 representing progressive degrees of abnormality |
| Disc bulging (anteriorly and posteriorly) | 0-3 | 0 equaling normal and 1 through 3 representing progressive degrees of abnormality (if bulging was detected both anteriorly and posteriorly, the larger of the ratings was used |
| Disc herniation, high intensity zones, osteophytes, upper endplate irregularities and fatty degeneration of the vertebrae | 0-3 | 0 equaling normal and 1 through 3 representing progressive degrees of abnormality |

**Table 63.** Grading system for lumbar disc degeneration proposed by Videman [69]

*MRI-based grading systems that did not specify the grading components used to measure disc degeneration*

| **Grading components** | **Grade** |
| --- | --- |
| Not specified | 1-5 |

**Table 64.** Grading system for lumbar disc degeneration proposed by Bajpai [70]

| **Grading components** | **Grade** |
| --- | --- |
| Unspecified | Degenerative status was classified into five grades according to the criteria of Frymoyer and Moskowitz |

**Table 65.** Grading system for lumbar disc degeneration as reported in Park [71]

| **Grading components** | **Description** |
| --- | --- |
| Single level disc degeneration | Not specified |
| Multi-level disc degeneration | Not specified |

**Table 66.** Grading system for lumbar disc degeneration proposed by Manev [72]

| **Grading components** | **Description** |
| --- | --- |
| Disc degeneration | Not specified |
|  |  |

**Table 67.** Grading system for lumbar disc degeneration proposed by Hupli [73]

| **Grading components** | **Grade** |
| --- | --- |
| Unspecified | Disc degeneration was graded between 1-5 whereby grade 1 and 2 were considered normal, and grade 3-5 was accepted as the presence of degeneration |

**Table 68.** Grading system for lumbar disc degeneration proposed by Sivas [74]

**Reference List**

1. Decandido P, Reinig JW, Dwyer AJ, Thompson KJ, Ducker TB. Magnetic Resonance Assessment of the Distribution of Lumbar Spine Disc Degenerative Changes. *J Spinal Disord*. 1988;1(1):9-15.

2. Dimar JR, 2nd, Glassman SD, Carreon LY. Juvenile Degenerative Disc Disease: A Report of 76 Cases Identified by Magnetic Resonance Imaging. Research Support, Non-U.S. Gov't. *Spine Journal: Official Journal of the North American Spine Society*. May-Jun 2007;7(3):332-7.

3. Evans W, Jobe W, Seibert C, Evans W, Jobe W, Seibert C. A Cross-Sectional Prevalence Study of Lumbar Disc Degeneration in a Working Population. *Spine (03622436)*. 1989;14(1):60-64.

4. Dragsbaek L, Kjaer P, Hancock M, Jensen TS. An Exploratory Study of Different Definitions and Thresholds for Lumbar Disc Degeneration Assessed by Mri and Their Associations with Low Back Pain Using Data from a Cohort Study of a General Population. *BMC Musculoskeletal Disorders*. Apr 17 2020;21(1):253. doi:https://dx.doi.org/10.1186/s12891-020-03268-4

5. Eyre D, ; Nemya P.; Buckwalter. Intervertebral Disk: Basic Science Perspectives. *New perspectives on low back pain*. 1989;

6. Fu MC, Buerba RA, Long WD, 3rd, Blizzard DJ, Lischuk AW, Haims AH, et al. Interrater and Intrarater Agreements of Magnetic Resonance Imaging Findings in the Lumbar Spine: Significant Variability across Degenerative Conditions. *Spine Journal: Official Journal of the North American Spine Society*. Oct 01 2014;14(10):2442-8. doi:https://dx.doi.org/10.1016/j.spinee.2014.03.010

7. Gibson MJ, Buckley J, Mawhinney R, Mulholland RC, Worthington BS. Magnetic Resonance Imaging and Discography in the Diagnosis of Disc Degeneration. A Comparative Study of 50 Discs. *Journal of Bone and Joint Surgery - Series B*. 1986;68(3):369-373. doi:https://dx.doi.org/10.1302/0301-620x.68b3.3733797

8. Heithoff KB, Gundry CR, Burton CV, Winter RB, Heithoff KB, Gundry CR, et al. Juvenile Discogenic Disease. *Spine (03622436)*. 1994;19(3):335-340.

9. Ito M, Incorvaia KM, Yu SF, Fredrickson BE, Yuan HA, Rosenbaum AE. Predictive Signs of Discogenic Lumbar Pain on Magnetic Resonance Imaging with Discography Correlation. Comparative Study. *Spine*. Jun 01 1998;23(11):1252-8; discussion 1259-60.

10. Kotilainen E, Alanen A, Erkintalo M, Valtonen S, Kormano M. Association between Decreased Disc Signal Intensity in Preoperative T2-Weighted Mri and a 5-Year Outcome after Lumbar Minimally Invasive Discectomy. Research Support, Non-U.S. Gov't. *Minimally Invasive Neurosurgery*. Mar 2001;44(1):31-6.

11. Linson MA, Crowe CH. Comparison of Magnetic Resonance Imaging and Lumbar Discography in the Diagnosis of Disc Degeneration. Comparative Study. *Clinical Orthopaedics & Related Research*. Jan 1990;(250):160-3.

12. Liuke M, Solovieva S, Lamminen A, Luoma K, Leino-Arjas P, Luukkonen R, et al. Disc Degeneration of the Lumbar Spine in Relation to Overweight. Research Support, Non-U.S. Gov't. *International Journal of Obesity*. Aug 2005;29(8):903-8.

13. Luoma K, Riihimaki H, Luukkonen R, Raininko R, Viikari-Juntura E, Lamminen A. Low Back Pain in Relation to Lumbar Disc Degeneration. Research Support, Non-U.S. Gov't. *Spine*. Feb 15 2000;25(4):487-92.

14. Madan SS, Rai A, Harley JM. Interobserver Error in Interpretation of the Radiographs for Degeneration of the Lumbar Spine. Comparative Study. *Iowa Orthopaedic Journal*. 2003;23:51-6.

15. Marchiori DM, Mclean I, Firth R, Tatum R. A Comparison of Radiographic Signs of Degeneration to Corresponding Mri Signal Intensities in the Lumbar Spine. Comparative Study. *Journal of Manipulative & Physiological Therapeutics*. May 1994;17(4):238-45.

16. Maurer M, Soder RB, Baldisserotto M. Spine Abnormalities Depicted by Magnetic Resonance Imaging in Adolescent Rowers. Comparative Study. *American Journal of Sports Medicine*. Feb 2011;39(2):392-7. doi:https://dx.doi.org/10.1177/0363546510381365

17. Tertti M, Paajanen H, Kujala UM, Alanen A, Salmi TT, Kormano M. Disc Degeneration in Young Gymnasts. A Magnetic Resonance Imaging Study. *American Journal of Sports Medicine*. Mar-Apr 1990;18(2):206-8.

18. Raininko R, Manninen H, Battie MC, Gibbons LE, Gill K, Fisher LD. Observer Variability in the Assessment of Disc Degeneration on Magnetic Resonance Images of the Lumbar and Thoracic Spine. Research Support, U.S. Gov't, P.H.S.Twin Study. *Spine*. May 01 1995;20(9):1029-35.

19. Videman T, Levalahti E, Battie MC. The Effects of Anthropometrics, Lifting Strength, and Physical Activities in Disc Degeneration. Research Support, N.I.H., ExtramuralResearch Support, Non-U.S. Gov't Twin Study. *Spine*. Jun 01 2007;32(13):1406-13.

20. Borenstein DG, O'mara JW, Jr., Boden SD, Lauerman WC, Jacobson A, Platenberg C, et al. The Value of Magnetic Resonance Imaging of the Lumbar Spine to Predict Low-Back Pain in Asymptomatic Subjects : A Seven-Year Follow-up Study. *Journal of Bone & Joint Surgery - American Volume*. Sep 2001;83(9):1306-11.

21. Buttermann GR, Mullin WJ, Buttermann GR, Mullin WJ. Pain and Disability Correlated with Disc Degeneration Via Magnetic Resonance Imaging in Scoliosis Patients. *European Spine Journal*. 2008;17(2):240-249. doi:10.1007/s00586-007-0530-8

22. Jensen RK, Kent P, Jensen TS, Kjaer P. The Association between Subgroups of Mri Findings Identified with Latent Class Analysis and Low Back Pain in 40-Year-Old Danes. Observational Study Research Support, Non-U.S. Gov't. *BMC Musculoskeletal Disorders*. 02 20 2018;19(1):62. doi:https://dx.doi.org/10.1186/s12891-018-1978-x

23. Lakadamyali H, Tarhan NC, Ergun T, Cakir B, Agildere AM. Stir Sequence for Depiction of Degenerative Changes in Posterior Stabilizing Elements in Patients with Lower Back Pain. *AJR. American Journal of Roentgenology*. Oct 2008;191(4):973-9. doi:https://dx.doi.org/10.2214/AJR.07.2829

24. Leboeuf-Yde C, Kjaer P, Bendix T, Manniche C. Self-Reported Hard Physical Work Combined with Heavy Smoking or Overweight May Result in So-Called Modic Changes. Comparative StudyResearch Support, Non-U.S. Gov't. *BMC Musculoskeletal Disorders*. Jan 14 2008;9:5. doi:https://dx.doi.org/10.1186/1471-2474-9-5

25. Luoma K, Vehmas T, Kerttula L, Gronblad M, Rinne E. Chronic Low Back Pain in Relation to Modic Changes, Bony Endplate Lesions, and Disc Degeneration in a Prospective Mri Study. *European Spine Journal*. 09 2016;25(9):2873-81. doi:https://dx.doi.org/10.1007/s00586-016-4715-x

26. Sabnis AB, Chamoli U, Diwan AD. Is L5-S1 Motion Segment Different from the Rest? A Radiographic Kinematic Assessment of 72 Patients with Chronic Low Back Pain. Research Support, Non-U.S. Gov't. *European Spine Journal*. 05 2018;27(5):1127-1135. doi:https://dx.doi.org/10.1007/s00586-017-5400-4

27. Schneiderman G, Flannigan B, Kingston S, Thomas J, Dillin WH, Watkins RG, et al. Magnetic Resonance Imaging in the Diagnosis of Disc Degeneration: Correlation with Discography. *Spine (03622436)*. 1987;12(3):276-281.

28. Karppinen J, Paakko E, Paassilta P, Lohiniva J, Kurunlahti M, Tervonen O, et al. Radiologic Phenotypes in Lumbar Mr Imaging for a Gene Defect in the Col9a3 Gene of Type Ix Collagen. *Radiology*. 01 Apr 2003;227(1):143-148. doi:https://dx.doi.org/10.1148/radiol.2271011821

29. Stadnik TW, Lee RR, Coen HL, Neirynck EC, Buisseret TS, Osteaux MJC. Annular Tears and Disk Herniation: Prevalence and Contrast Enhancement on Mr Images in the Absence of Low Back Pain or Sciatica. *Radiology*. January 1998;206(1):49-55. doi:http://dx.doi.org/10.1148/radiology.206.1.9423651

30. Throckmorton TW, Hilibrand AS, Mencio GA, Hodge A, Spengler DM. The Impact of Adjacent Level Disc Degeneration on Health Status Outcomes Following Lumbar Fusion. *Spine*. Nov 15 2003;28(22):2546-50.

31. Battie MC, Videman T, Levalahti E, Gill K, Kaprio J. Genetic and Environmental Effects on Disc Degeneration by Phenotype and Spinal Level: A Multivariate Twin Study. Comparative Study Research Support, N.I.H., Extramural Research Support, Non-U.S. Gov't Twin Study. *Spine*. Dec 01 2008;33(25):2801-8. doi:https://dx.doi.org/10.1097/BRS.0b013e31818043b7

32. Battie MC, Videman T, Gibbons LE, Fisher LD, Manninen H, Gill K. Determinants of Lumbar Disc Degeneration: A Study Relating Lifetime Exposures and Magnetic Resonance Imaging Findings in Identical Twins. *Spine*. 1995;20(24):2601-2612.

33. Deng C, Xia W. Effect of Tai Chi Chuan on Degeneration of Lumbar Vertebrae and Lumbar Discs in Middle-Aged and Aged People: A Cross-Sectional Study Based on Magnetic Resonance Images. *Journal of International Medical Research*. Feb 2018;46(2):578-585. doi:https://dx.doi.org/10.1177/0300060517734115

34. Desigan S, Hall-Craggs MA, Ho CP, Eliahoo J, Porter JB. Degenerative Disc Disease as a Cause of Back Pain in the Thalassaemic Population: A Case-Control Study Using Mri and Plain Radiographs. Controlled Clinical Trial. *Skeletal Radiology*. Feb 2006;35(2):95-102.

35. Fardon DF, Milette PC. Nomenclature and Classification of Lumbar Disc Pathology. Recommendations of the Combined Task Forces of the North American Spine Society, American Society of Spine Radiology, and American Society of Neuroradiology. *Spine (Philadelphia, Pa. 1976)*. 2001;26(5):E93-E113. doi:10.1097/00007632-200103010-00006

36. Kiil RM, Mistegaard CE, Loft AG, Zejden A, Hendricks O, Jurik AG. Differences in Topographical Location of Sacroiliac Joint Mri Lesions in Patients with Early Axial Spondyloarthritis and Mechanical Back Pain. *Arthritis Research & Therapy*. 03 24 2022;24(1):75. doi:https://dx.doi.org/10.1186/s13075-022-02760-7

37. Horton WC, Daftari TK. Which Disc as Visualized by Magnetic Resonance Imaging Is Actually a Source of Pain? A Correlation between Magnetic Resonance Imaging and Discography. *Spine (Phila Pa 1976)*. Jun 1992;17(6 Suppl):S164-71. doi:10.1097/00007632-199206001-00018

38. Kanamori M, Nobukiyo M, Suzuki K, Yasuda T, Hori T. Clinical Validity of a New T2-Weighted Mri-Based Grading System for Lumbar Disc Degeneration. *International Medical Journal*. August 2013;20(4):466-469.

39. Solovieva S, Lohiniva J, Leino-Arjas P, Raininko R, Luoma K, Ala-Kokko L, et al. Col9a3 Gene Polymorphism and Obesity in Intervertebral Disc Degeneration of the Lumbar Spine: Evidence of Gene-Environment Interaction. *Spine (03622436)*. 2002;27(23):2691-2696.

40. Videman T, Gibbons LE, Battie MC. Age-and Pathology-Specific Measures of Disc Degeneration. *Spine (Philadelphia, Pa. 1976)*. 2008;33(25):2781-2788. doi:10.1097/brs.0b013e31817e1d11

41. Videman T, Battie MC, Gibbons LE, Manninen H, Gill K, Fisher LD, et al. Lifetime Exercise and Disk Degeneration: An Mri Study of Monozygotic Twins. Research Support, Non-U.S. Gov't Research Support, U.S. Gov't, P.H.S. Twin Study. *Medicine & Science in Sports & Exercise*. Oct 1997;29(10):1350-6.

42. Witwit WA, Kovac P, Sward A, Agnvall C, Todd C, Thoreson O, et al. Disc Degeneration on Mri Is More Prevalent in Young Elite Skiers Compared to Controls. Observational Study. *Knee Surgery, Sports Traumatology, Arthroscopy*. Jan 2018;26(1):325-332. doi:https://dx.doi.org/10.1007/s00167-017-4545-3

43. Buirski G, Silberstein M, Buirski G, Silberstein M. The Symptomatic Lumbar Disc in Patients with Low-Back Pain. Magnetic Resonance Imaging Appearances in Both a Symptomatic and Control Population. *Spine (03622436)*. 1993;18(13):1808-1811.

44. Butler D, Trafimow JH, Andersson GB, Mcneill TW, Huckman MS. Discs Degenerate before Facets. *Spine (Phila Pa 1976)*. Feb 1990;15(2):111-3. doi:10.1097/00007632-199002000-00012

45. Griffith JF, Wang YX, Antonio GE, Choi KC, Yu A, Ahuja AT, et al. Modified Pfirrmann Grading System for Lumbar Intervertebral Disc Degeneration. Clinical Trial Validation Study. *Spine*. Nov 15 2007;32(24):E708-12.

46. Kealey SM, Aho T, Delong D, Barboriak DP, Provenzale JM, Eastwood JD. Assessment of Apparent Diffusion Coefficient in Normal and Degenerated Intervertebral Lumbar Disks: Initial Experience. Research Support, U.S. Gov't, Non-P.H.S. *Radiology*. May 2005;235(2):569-74.

47. Kjaer P, Leboeuf-Yde C, Sorensen JS, Bendix T. An Epidemiologic Study of Mri and Low Back Pain in 13-Year-Old Children. Research Support, Non-U.S. Gov't. *Spine*. Apr 01 2005;30(7):798-806.

48. Lei D, Rege A, Koti M, Smith FW, Wardlaw D. Painful Disc Lesion: Can Modern Biplanar Magnetic Resonance Imaging Replace Discography? Comparative Study. *Journal of Spinal Disorders & Techniques*. Aug 2008;21(6):430-5. doi:https://dx.doi.org/10.1097/BSD.0b013e318153f7e4

49. Chen JY, Ding Y, Lv RY, Liu QY, Huang JB, Yang ZH, et al. Correlation between Mr Imaging and Discography with Provocative Concordant Pain in Patients with Low Back Pain. *Clinical Journal of Pain*. 2011;27(2):125-130. doi:10.1097/AJP.0b013e3181fb2203

50. Lim CH, Jee WH, Son BC, Kim DH, Ha KY, Park CK. Discogenic Lumbar Pain: Association with Mr Imaging and Ct Discography. *European Journal of Radiology*. Jun 2005;54(3):431-7.

51. Pfirrmann CW, Metzdorf A, Zanetti M, Hodler J, Boos N. Magnetic Resonance Classification of Lumbar Intervertebral Disc Degeneration. Research Support, Non-U.S. Gov't Validation Study. *Spine*. Sep 01 2001;26(17):1873-8.

52. Battie MC, Videman T, Gibbons LE, Manninen H, Gill K, Pope M, et al. Occupational Driving and Lumbar Disc Degeneration: A Case-Control Study. Research Support, Non-U.S. Gov't

Research Support, U.S. Gov't, P.H.S. *Lancet*. Nov 02 2002;360(9343):1369-74.

53. Videman T, Battie MC, Gibbons LE, Kaprio J, Koskenvuo M, Kannus P, et al. Disc Degeneration and Bone Density in Monozygotic Twins Discordant for Insulin-Dependent Diabetes Mellitus. Research Support, Non-U.S. Gov't Research Support, U.S. Gov't, P.H.S. Twin Study. *Journal of Orthopaedic Research*. Sep 2000;18(5):768-72.

54. Battie MC, Levalahti E, Videman T, Burton K, Kaprio J. Heritability of Lumbar Flexibility and the Role of Disc Degeneration and Body Weight. Research Support, N.I.H., Extramural Research Support, Non-U.S. Gov't Twin Study. *Journal of Applied Physiology*. Feb 2008;104(2):379-85.

55. Battie MC, Videman T, Levalahti E, Gill K, Kaprio J. Heritability of Low Back Pain and the Role of Disc Degeneration. Research Support, N.I.H., Extramural Research Support, Non-U.S. Gov't Twin Study. *Pain*. Oct 2007;131(3):272-280. doi:https://dx.doi.org/10.1016/j.pain.2007.01.010

56. Bechara BP, Agarwal V, Boardman J, Perera S, Weiner DK, Vo N, et al. Correlation of Pain with Objective Quantification of Magnetic Resonance Images in Older Adults with Chronic Low Back Pain. Research Support, N.I.H., Extramural Research Support, Non-U.S. Gov't. *Spine*. Mar 15 2014;39(6):469-75. doi:https://dx.doi.org/10.1097/BRS.0000000000000181

57. Benneker LM, Heini PF, Anderson SE, Alini M, Ito K. Correlation of Radiographic and Mri Parameters to Morphological and Biochemical Assessment of Intervertebral Disc Degeneration. Comparative Study Research Support, Non-U.S. Gov't Validation Study. *European Spine Journal*. Feb 2005;14(1):27-35.

58. Djurasovic M, Carreon LY, Crawford CH, 3rd, Zook JD, Bratcher KR, Glassman SD. The Influence of Preoperative Mri Findings on Lumbar Fusion Clinical Outcomes. *European Spine Journal*. Aug 2012;21(8):1616-23. doi:https://dx.doi.org/10.1007/s00586-012-2244-9

59. Frobin W, Brinckmann P, Kramer M, Hartwig E. Height of Lumbar Discs Measured from Radiographs Compared with Degeneration and Height Classified from Mr Images. Clinical Trial Comparative Study Randomized Controlled Trial. *European Radiology*. 2001;11(2):263-9.

60. Sambrook PN, Macgregor AJ, Spector TD. Genetic Influences on Cervical and Lumbar Disc Degeneration: A Magnetic Resonance Imaging Study in Twins. Comparative Study Research Support, Non-U.S. Gov't Twin Study. *Arthritis & Rheumatism*. Feb 1999;42(2):366-72.

61. Jarosz J, Bingham J, Pemberton J, Sambrook P, Spector T. An Atlas for Scoring Cervical and Lumbar Disc Degeneration. London: Springer Verlag; 1997.

62. Jiang X, Chen D, Li Z, Lou Y. Correlation between Lumbar Spine Facet Joint Orientation and Intervertebral Disk Degeneration: A Positional Mri Analysis. *Journal of Neurological Surgery*. Jul 2019;80(4):255-261. doi:https://dx.doi.org/10.1055/s-0039-1683450

63. Kilitci A, Asan Z, Yuceer A, Aykanat O, Durna F. Comparison of the Histopathological Differences between the Spinal Material and Posterior Longitudinal Ligament in Patients with Lumbar Disc Herniation: A Focus on the Etiopathogenesis. *Annals of Saudi Medicine*. Mar-Apr 2021;41(2):115-120. doi:https://dx.doi.org/10.5144/0256-4947.2021.115

64. Luoma K, Vehmas T, Raininko R, Luukkonen R, Riihimaki H. Lumbosacral Transitional Vertebra: Relation to Disc Degeneration and Low Back Pain. Research Support, Non-U.S. Gov't. *Spine*. Jan 15 2004;29(2):200-5.

65. Boos N, Dreier D, Hilfiker E, Schade V, Kreis R, Hora J, et al. Tissue Characterization of Symptomatic and Asymptomatic Disc Herniations by Quantitative Magnetic Resonance Imaging. Comparative Study Research Support, Non-U.S. Gov't. *Journal of Orthopaedic Research*. Jan 1997;15(1):141-9.

66. Thalgott JS, Albert TJ, Vaccaro AR, Aprill CN, Giuffre JM, Drake JS, et al. A New Classification System for Degenerative Disc Disease of the Lumbar Spine Based on Magnetic Resonance Imaging, Provocative Discography, Plain Radiographs and Anatomic Considerations. Review. *Spine Journal: Official Journal of the North American Spine Society*. Nov-Dec 2004;4(6 Suppl):167S-172S.

67. Riesenburger RI, Safain MG, Ogbuji R, Hayes J, Hwang SW. A Novel Classification System of Lumbar Disc Degeneration. *Journal of Clinical Neuroscience*. Feb 2015;22(2):346-51. doi:https://dx.doi.org/10.1016/j.jocn.2014.05.052

68. Burke SM, Hwang SW, Mehan WA, Jr., Bedi HS, Ogbuji R, Riesenburger RI. Reliability of the Modified Tufts Lumbar Degenerative Disc Classification between Neurosurgeons and Neuroradiologists. *Journal of Clinical Neuroscience*. Jul 2016;29:111-6. doi:https://dx.doi.org/10.1016/j.jocn.2015.10.040

69. Videman T, Battié MC, Ripatti S, Gill K, Manninen H, Kaprio J, et al. Determinants of the Progression in Lumbar Degeneration: A 5-Year Follow-up Study of Adult Male Monozygotic Twins. *Spine (03622436)*. 2006;31(6):671-678. doi:10.1097/01.brs.0000202558.86309.ea

70. Bajpai J, Saini S, Singh R. Clinical Correlation of Magnetic Resonance Imaging with Symptom Complex in Prolapsed Intervertebral Disc Disease: A Cross-Sectional Double Blind Analysis. *Journal of Craniovertebral Junction & Spine*. Jan 2013;4(1):16-20. doi:https://dx.doi.org/10.4103/0974-8237.121619

71. Park JB, Chang H, Kim KW, Park SJ. Facet Tropism: A Comparison between Far Lateral and Posterolateral Lumbar Disc Herniations. Comparative Study. *Spine*. Mar 15 2001;26(6):677-9.

72. Manav V, Ilhan D, Mercan H, Kilic A, Polat AK, Aksu AEK. Association between Intervertebral Disc Degeneration and Behcet's Disease. *Dermatologic Therapy*. 07 2022;35(7):e15585. doi:https://dx.doi.org/10.1111/dth.15585

73. Hupli M, Heinonen R, Vanharanta H. Height Changes among Chronic Low Back Pain Patients During Intense Physical Exercise. Research Support, Non-U.S. Gov't. *Scandinavian Journal of Medicine & Science in Sports*. Feb 1997;7(1):32-7.

74. Sivas FA, Ciliz D, Erel U, Inal EE, Özoran K, Sakman B. Abnormal Lumbar Magnetic Resonance Imaging in Asymptomatic Individuals. *Turkish Journal of Physical Medicine & Rehabilitation / Turkiye Fiziksel Tip ve Rehabilitasyon Dergisi*. 2009;55(2):73-77.
