# Supplementary material for: MRI‐Based Grading Systems for Assessing Lumbar Disc Degeneration: A Scoping Review
Source: JOR Spine. 2025 Sep 15;8(3):e70113. doi: 10.1002/jsp2.70113 (PMC12435304; doi:10.1002/jsp2.70113)
Supplement: Supplementary file 4 — Data S4: Supporting Information. [file JSP2-8-e70113-s005.docx]

**Online Resource 4.**

Quantitative grading systems for lumbar disc degeneration on MRI

*MRI-based grading systems that used CSF-adjusted disc signal intensity alone in the assessment of degenerative disc disease in the lumbar spine*

| **Quantitative components** | **Description** |
| --- | --- |
| CSF-adjusted disc signal intensity | An ellipsoid region of interest (ROI) was digitally marked from each nucleus pulposus. As an internal reference, the SI of the adjacent cerebrospinal fluid was used, resulting in a disc to CSF-SI ratio (SINDAHL) |

**Table 1.** Grading system for lumbar disc degeneration proposed by Aavikko [1]

| **Quantitative components** | **Description** |
| --- | --- |
| CSF-adjusted disc signal intensity | Ratio of the mean signal intensity of the entire disc to the adjacent CSF signal intensity. Calculated by drawing outlines of vertebrae, discs, and the adjacent CSF samples |

**Table 2.** Grading system for lumbar disc degeneration as reported in Battie [2]

Proposed by Battie [3]

| **Grading components** | **Grades** |
| --- | --- |
| Quantitative normalised disc signal intensity  (Gray value and pixels of the nucleus pulposus and gray value of CSF in adjoining domains was measured. The average relative signal intensity was divided into four grades by hierarchical clustering analysis) | 0-4 |

**Table 3.** Grading system for lumbar disc degeneration proposed by Ding [4]

| **Quantitative components** | **Description** |
| --- | --- |
| Normalised disc signal intensity | A semi-quantitative measure was derived by using the mean voxel intensity in each nucleus pulposus. Then another ROI was drawn in the uniform region of the gray matter of the spinal cord and the mean intensity was calculated. Then, the mean intensity in each disc was divided by the mean intensity in the spinal cord gray matter to obtain a metric that can be compared across subjects. |

**Table 4.** Grading system for lumbar disc degeneration proposed by Jarman [5]

| **Quantitative components** | **Description** |
| --- | --- |
| Computerised analysis of the signal intensity of the nucleus pulposus | For signal intensity measurements, the spatially dependent inhomogeneity of the signal intensity in the surface coil images were corrected by a computerised method. Regions of interest above and below the central intranuclear cleft in each nucleus pulposus and in cerebrospinal fluid (CSF) in the anterior part of the adjacent dural sac behind each vertebra were defined, and their signal intensities measured. |

**Table 5.** Grading system for lumbar disc degeneration proposed by Luoma [6]

| **Quantitative components** | **Description** |
| --- | --- |
| CSF-adjusted disc signal intensity | The signal intensity of the disc was assessed quantitatively by a computerised method with a region of interest (ROI) marked digitally from each nucleus pulposus. As an internal reference, the SI of the adjacent CSF was used for a disc to CSF-SI ratio. For the ROI of the CSF at every level, the area in the anterior dural sac immediately posterior to the disc was chosen to exclude the effect of the nerve roots. |

**Table 6.** Grading system for lumbar disc degeneration proposed by Lund [7]

| **Quantitative components** | **Description** |
| --- | --- |
| CSF-adjusted disc signal intensity | The intervertebral area was defined as the quadrangle formed by the anterior and posterior edges of the upper and lower endplates in contact with the intervertebral disc to be measured. A shape similar to the intervertebral area but with one fourth of the area was drawn. The geometric shape center of the shape was matched to the centre of intensity, and this shape was used as the region of interest for measuring intensity of the nucleus pulposus. The signal intensity of the nucleus pulposis was expressed as a percentage of the intensity of the CSF. |

**Table 7.** Grading system for lumbar disc degeneration proposed by Nagashima [8]

The degree of degenerative disc disease was defined as the mean signal intensity of the 6 intervertebral discs from T12-L1 to L5-S1

| **Grading components** | **Description** |
| --- | --- |
| CSF-adjusted disc signal intensity | A region of interest (ROI) was determined in the nucleus pulposus of each disc from T12-L1 to L5-S1. The disc with the highest SI value was regarded as the healthiest in each subject and used as a reference point. A relative SI value was calculated as a percentage of the reference disc. |

**Table 8.** Grading system for lumbar disc degeneration proposed by Paajanen [9]

Disc signal intensity was graded ordinally using a 3-point scale from bright/normal to absent/marked degeneration

| **Grading components** | **Description** |
| --- | --- |
| CSF-adjusted disc signal intensity | Disc signal intensity was measured using a midsagittal disc signal, which was adjusted according to the signal intensity of an adjacent cerebrospinal fluid sample extracted from digital MRI data, using a custom-designed image analysis program (Spine Examiner) |

**Table 9.** Grading system for lumbar disc degeneration proposed by Videman [10]

| **Quantitative components** | **Description** |
| --- | --- |
| Disc signal intensity | Used a ratio of the mean signal strength in the nucleus area to the mean in the annulus region. SpIn (for spine insight) were conducted, which passed through the centre of the disc between the two end plates. Nucleus and annulus regions were defined as preset proportions of the axial disc area. The first axial measure, Axial SpIn1, was based on the ratio of the mean signal of the central 75% of the disc area, including the entire nucleus area, to the outer annulus region, which was the ring along the perimeter of the disc comprising 25% of the total disc area. One was subtracted from the ratio and then multiplied by 10 to create informative scores.  Axial SpIn2 was constructed to determine if weighting regions of the nucleus and inner annulus equally would improve measurement. The central 25% of the total disc area represented the nucleus, the ring along the perimeter of the disc comprising 25% of the total disc area represented the outer annulus, and the intermediate area comprising 50% of the disc represented the inner annulus. The mean signal of each was divided by the mean signal strength of the outer annulus, and one was subtracted. The scores for each region were averaged and then multiplied by 10. |

**Table 10.** Grading system for lumbar disc degeneration proposed by Videman [11]

*MRI-based grading systems that used quantitative disc height alone in the assessment of degenerative disc disease in the lumbar spine*

| **Quantitative components** | **Description** |
| --- | --- |
| Disc height | The anterior and posterior heights of the intervertebral discs were measured in the middle line of the disc from the proton density-weighted sagittal images. The shortest distance between the anterior and posterior edge of the neighboring end plates was measured with MRI software. Their mean-distance was considered to represent the disc height |

**Table 11.** Grading system for lumbar disc degeneration proposed by Luoma [6]

| **Quantitative components** | **Description** |
| --- | --- |
| Disc height | Quantitative height was obtained from the midsagittal section by dividing the disc area contained between the theoretical vertebral borders (corners) by the diameter of the area |

**Table 12.** Grading system for lumbar disc degeneration as reported in Hancock [12]

Proposed by Battie [2]

**Table 13.** Grading system for lumbar disc degeneration proposed by Jarman [5]

| **Quantitative components** | **Description** |
| --- | --- |
| Disc height index | The proximal and distal vertebral body height and intervertebral height were measured from the anterior, middle, and posterior portions of each respective disc level on T2 images. The measurements were performed on the midsagittal slice. The corners of the vertebral bodies and the midpoints of the endplates were marked. The measurement lines were drawn between those landmarks and distance measurements were taken |

*MRI-based grading systems that used quantitative measurements of disc bulging in the assessment of degenerative disc disease in the lumbar spine*

| **Quantitative components** | **Description** |
| --- | --- |
| Disc bulge ratio using the disc area | Disc areas were measured and the average of the four-disc areas were used as a disc area. Two lines connecting the middle points of the anterior and posterior borders of two adjacent vertebral bodies were drawn. The anterior and posterior areas protruding from these lines were measured. The average was obtained of the protruding areas in the four discs and used as a bulging disc area. The disc bulge ratio was calculated from the ratio of the bulging disc area and disc area |

**Table 14.** Grading system for lumbar disc degeneration proposed by Harada [13]

| **Quantitative components** | **Description** |
| --- | --- |
| Anterior and posterior bulge of the intervertebral disc | The magnitude of disc bulges was measured in the middle line of the disc from the proton density-weighted sagittal images. A bulge of ≥3.2 mm was considered positive for degenerative disc disease |

**Table 15.** Grading system for lumbar disc degeneration proposed by Luoma [14]

*MRI-based grading systems that used CSF-adjusted disc signal intensity, and quantitative disc height in the assessment of degenerative disc disease in the lumbar spine*

| **Quantitative components** | **Description** |
| --- | --- |
| Disc area | Percent area parameter for each disc was calculated as the area of that disc divided by the sum of all disc areas in that subject |
| Sum of pixel intensities | Calculated as a percentage of the total sum intensity across all discs for each subject respectively |
| MRI index parameter | This was calculated as a product of the disc’s area and sum intensity. Lower values indicate a degenerated disc while higher values indicate a healthy disc |

**Table 16.** Grading system for lumbar disc degeneration proposed by Bechara [15]

Discs were segmented according to atlas-based segmentation using fuzzy c-means algorithm

| **Quantitative components** | **Description** |
| --- | --- |
| Disc signal intensity | 3 different measures of disc signal intensity were used. A raw disc signal intensity measure, a ratio adjusted for brightness of CSF at the same level, and a ratio adjusted for brightest level of CSF at any of the 5 spinal levels. Raw signal intensity was recorded for each disc area as defined above for measurement of disc height. Ratio 1 was calculated by dividing the raw disc signal intensity for each vertebral level by the signal intensity of the CSF at the adjacent level. Ratio 2 was calculated by dividing the raw disc signal intensity by the signal intensity of the most intense CSF at any of the 5 spinal levels. |
| Disc height | 3 different measures of disc height were used. A raw disc height measure, a ratio adjusted for each person’s height, and a ratio adjusted for height of the vertebral body about the disc. Raw disc height was measured by dividing the disc area by horizontal length. Disc area was defined by using the freehand region of interest measurement tool and tracing around the disc starting along the anterior longitudinal ligament. Ratio 1 was calculated by dividing the raw disc height for each vertebral level by the total body height of the participant. Ratio 2 was calculated by dividing the raw disc height by the height of the vertebral body above the disc. The height of the vertebral body above was calculated in a similar matter to disc height |

**Table 17.** Grading system for lumbar disc degeneration as reported by Salamat [16]

CSF adjusted disc signal intensity and disc height as proposed by Battie [3]

| **Quantitative components** | **Description** |
| --- | --- |
| CSF adjusted disc signal intensity | The intervertebral disc signals in the anterior, middle, and posterior regions of the nucleus pulposus were measured and averaged and further adjusted by the adjacent CSF signal |
| Disc height* | Disc height was defined as the average of the anterior, middle, and posterior heights of the nonconvex portion of the disc |

**Table 18.** Grading system for lumbar disc degeneration proposed by Su[17]

*MRI-based grading systems that used CSF-adjusted disc signal intensity, quantitative disc height, and quantitative measurements of disc bulging in the assessment of degenerative disc disease in the lumbar spine*

| **Quantitative components** | **Description** |
| --- | --- |
| CSF-adjusted disc signal intensity | Ratio of the mean signal intensity of the entire disc to the adjacent CSF signal intensity. Calculated by drawing outlines of vertebrae, discs, and the adjacent CSF samples |
| Disc height | Mean of the four height measurements: 1) Anterior corners of the adjacent vertebrae, 2) posterior corners of the adjacent vertebrae, 3) distances on either side of the nucleus |
| Disc bulging | Relative degree was indicated by measuring disc width at its center point. Computer drawn lines based on a standard algorithm |

**Table 19.** Grading system for lumbar disc degeneration as reported in Hu [18]

Proposed by Battie [3]

| **Quantitative components** | **Description** |
| --- | --- |
| CSF-adjusted disc signal intensity | The mean disc signal intensity was acquired by defining a ROI for the intervertebral disc, which was further adjusted for adjacent CSF signal intensity |
| Disc height | Disc height was defined as the mean of anterior, middle, and posterior height of the intervertebral disc |
| Disc bulging | Disc bulging, which includes anterior and posterior bulging, was measured as the area of the portion of the disc that exceeds the anterior and posterior edges of vertebral bodies |

**Table 20.** Grading system for lumbar disc degeneration proposed by Feng [19]

| **Category** | **Parameter** |
| --- | --- |
| Diameter | Superior vertebra diameter  Middle vertebra diameter  Inferior vertebra diameter  Middle disc diameter |
| Height | Anterior vertebra height  Middle vertebra height  Posterior vertebra height  Anterior disc height  Middle disc height  Posterior disc height |
| Area | Vertebra area  Disc area  Anterior disc bulging area  Posterior disc bulging area |
| Signal | Mean vertebra signal intensity  SD of vertebra signal intensity  Mean disc signal intensity  Mean signal of adjacent CSF |

**Table 21.** Grading system for lumbar disc degeneration proposed by Huang [20]

Signal intensity was measured pixel by pixel in a defined ROI and thus, there are mean and standard deviation for signal intensity measurement

| **Quantitative components** | **Description** |
| --- | --- |
| Disc height | Disc height was defined as the mean of anterior, middle, and posterior heights of the non-convex portion of the disc |
| Disc bulging | Disc bulging included anterior and posterior bulging, which was measured as the area of the disc that exceeded the anterior or posterior edges of the adjacent vertebral bodies |
| CSF-adjusted disc signal intensity | Disc signals were sampled in the anterior, middle, and posterior regions of the nucleus pulposus, which were averaged and further adjusted using adjacent CSF signal |

**Table 22.** Grading system for lumbar disc degeneration proposed by Lu [21]

Acquired on a mid-sagittal T2W MR image

| **Quantitative components** | **Description** |
| --- | --- |
| CSF-adjusted disc signal intensity | The signal intensity of each nucleus pulposus was quantified by comparing it with cerebrospinal fluid. |
| Disc height | The means of the anterior and posterior heights were called disc heights |
| Disc bulging | A bulge measuring ≥3.2mm was considered a positive finding |

**Table 23.** Grading system for lumbar disc degeneration proposed by Luoma [22]

| **Quantitative components** | **Description** |
| --- | --- |
| Automation and computer aided grading for intensity, planar shape, and herniation | A novel method for automated diagnosis of degenerative disc disease using midsagittal MR images. The discs are first localised and segmented. Then, intensity, shape, context, and texture features of the discs are extracted with various techniques. A Support Vector Machine classifier is applied to classify the discs as normal or degeneration. The segmentation used planar shape (disc height), intensity (CSF adjusted signal intensity), and shape (herniation) |

**Table 24.** Grading system for lumbar disc degeneration proposed by Oktay [23]

**Reference List**

1. Aavikko A, Lohman M, Ristolainen L, Kautiainen H, Osterman K, Schlenzka D, et al. Issls Prize in Clinical Science 2022: Accelerated Disc Degeneration after Pubertal Growth Spurt Differentiates Adults with Low Back Pain from Their Asymptomatic Peers. Observational Study Research Support, Non-U.S. Gov't. *European Spine Journal*. 05 2022;31(5):1080-1087. doi:https://dx.doi.org/10.1007/s00586-022-07184-0

2. Battie MC, Videman T, Levalahti E, Gill K, Kaprio J. Genetic and Environmental Effects on Disc Degeneration by Phenotype and Spinal Level: A Multivariate Twin Study. Comparative Study Research Support, N.I.H., Extramural Research Support, Non-U.S. Gov't Twin Study. *Spine*. Dec 01 2008;33(25):2801-8. doi:https://dx.doi.org/10.1097/BRS.0b013e31818043b7

3. Battie MC, Videman T, Gibbons LE, Fisher LD, Manninen H, Gill K. Determinants of Lumbar Disc Degeneration: A Study Relating Lifetime Exposures and Magnetic Resonance Imaging Findings in Identical Twins. *Spine*. 1995;20(24):2601-2612.

4. Ding WY, Yang DL, Cao LZ, Sun YP, Zhang W, Xu JX, et al. Intervertebral Disc Degeneration and Bone Density in Degenerative Lumbar Scoliosis: A Comparative Study between Patients with Degenerative Lumbar Scoliosis and Patients with Lumbar Stenosis. *Chinese Medical Journal*. 2011;124(23):3875-3878. doi:https://dx.doi.org/10.3760/cma.j.issn.0366-6999.2011.23.008

5. Jarman JP, Arpinar VE, Baruah D, Klein AP, Maiman DJ, Muftuler LT. Intervertebral Disc Height Loss Demonstrates the Threshold of Major Pathological Changes During Degeneration. Research Support, Non-U.S. Gov't. *European Spine Journal*. Sep 2015;24(9):1944-50. doi:https://dx.doi.org/10.1007/s00586-014-3564-8

6. Luoma K, Vehmas T, Riihimäki H, Raininko R, Luoma K, Vehmas T, et al. Disc Height and Signal Intensity of the Nucleus Pulposus on Magnetic Resonance Imaging as Indicators of Lumbar Disc Degeneration. *Spine (03622436)*. 2001;26(6):680-686.

7. Lund T, Schlenzka D, Lohman M, Ristolainen L, Kautiainen H, Klemetti E, et al. The Intervertebral Disc During Growth: Signal Intensity Changes on Magnetic Resonance Imaging and Their Relevance to Low Back Pain. Research Support, Non-U.S. Gov't. *PLoS ONE [Electronic Resource]*. 2022;17(10):e0275315. doi:https://dx.doi.org/10.1371/journal.pone.0275315

8. Nagashima M, Abe H, Amaya K, Matsumoto H, Yanaihara H, Nishiwaki Y, et al. Risk Factors for Lumbar Disc Degeneration in High School American Football Players: A Prospective 2-Year Follow-up Study. *American Journal of Sports Medicine*. Sep 2013;41(9):2059-64. doi:https://dx.doi.org/10.1177/0363546513495173

9. Paajanen H, Erkintalo M, Kuusela T, Dahlstrom S, Kormano M. Magnetic Resonance Study of Disc Degeneration in Young Low-Back Pain Patients. Research Support, Non-U.S. Gov't. *Spine*. Sep 1989;14(9):982-5.

10. Videman T, Saarela J, Kaprio J, Nakki A, Levalahti E, Gill K, et al. Associations of 25 Structural, Degradative, and Inflammatory Candidate Genes with Lumbar Disc Desiccation, Bulging, and Height Narrowing. Research Support, N.I.H., Extramural Research Support, Non-U.S. Gov't. *Arthritis & Rheumatism*. Feb 2009;60(2):470-81. doi:https://dx.doi.org/10.1002/art.24268

11. Videman T, Battie MC, Gibbons LE, Gill K. A New Quantitative Measure of Disc Degeneration. *Spine Journal: Official Journal of the North American Spine Society*. 05 2017;17(5):746-753. doi:https://dx.doi.org/10.1016/j.spinee.2017.02.002

12. Hancock MJ, Battie MC, Videman T, Gibbons L. The Role of Back Injury or Trauma in Lumbar Disc Degeneration: An Exposure-Discordant Twin Study. Comparative Study Research Support, N.I.H., Extramural Research Support, Non-U.S. Gov't Twin Study. *Spine*. Oct 01 2010;35(21):1925-9. doi:https://dx.doi.org/10.1097/BRS.0b013e3181d60598

13. Harada A, Okuizumi H, Miyagi N, Genda E. Correlation between Bone Mineral Density and Intervertebral Disc Degeneration. Research Support, Non-U.S. Gov't. *Spine*. Apr 15 1998;23(8):857-61; discussion 862.

14. Luoma K, Riihimaki H, Luukkonen R, Raininko R, Viikari-Juntura E, Lamminen A. Low Back Pain in Relation to Lumbar Disc Degeneration. Research Support, Non-U.S. Gov't. *Spine*. Feb 15 2000;25(4):487-92.

15. Bechara BP, Agarwal V, Boardman J, Perera S, Weiner DK, Vo N, et al. Correlation of Pain with Objective Quantification of Magnetic Resonance Images in Older Adults with Chronic Low Back Pain. Research Support, N.I.H., Extramural Research Support, Non-U.S. Gov't. *Spine*. Mar 15 2014;39(6):469-75. doi:https://dx.doi.org/10.1097/BRS.0000000000000181

16. Salamat S, Hutchings J, Kwong C, Magnussen J, Hancock MJ. The Relationship between Quantitative Measures of Disc Height and Disc Signal Intensity with Pfirrmann Score of Disc Degeneration. *Springerplus*. 2016;5(1):829. doi:https://dx.doi.org/10.1186/s40064-016-2542-5

17. Su Y, Ren D, Chen Y, Geng L, Yao S, Wu H, et al. Effect of Endplate Reduction on Endplate Healing Morphology and Intervertebral Disc Degeneration in Patients with Thoracolumbar Vertebral Fracture. *European Spine Journal*. 01 2023;32(1):55-67. doi:https://dx.doi.org/10.1007/s00586-022-07215-w

18. Hu X, Chen M, Pan J, Liang L, Wang Y. Is It Appropriate to Measure Age-Related Lumbar Disc Degeneration on the Mid-Sagittal Mr Image? A Quantitative Image Study. Research Support, Non-U.S. Gov't. *European Spine Journal*. 05 2018;27(5):1073-1081. doi:https://dx.doi.org/10.1007/s00586-017-5357-3

19. Feng Z, Liu Y, Wei W, Hu S, Wang Y. Type Ii Modic Changes May Not Always Represent Fat Degeneration: A Study Using Mr Fat Suppression Sequence. *Spine*. Aug 15 2016;41(16):E987-E994. doi:https://dx.doi.org/10.1097/BRS.0000000000001526

20. Huang J, Shen H, Wu J, Hu X, Zhu Z, Lv X, et al. Spine Explorer: A Deep Learning Based Fully Automated Program for Efficient and Reliable Quantifications of the Vertebrae and Discs on Sagittal Lumbar Spine Mr Images. Research Support, Non-U.S. Gov't. *Spine Journal: Official Journal of the North American Spine Society*. 04 2020;20(4):590-599. doi:https://dx.doi.org/10.1016/j.spinee.2019.11.010

21. Lu X, Zhu Z, Pan J, Feng Z, Lv X, Battie MC, et al. Traumatic Vertebra and Endplate Fractures Promote Adjacent Disc Degeneration: Evidence from a Clinical Mr Follow-up Study. *Skeletal Radiology*. May 2022;51(5):1017-1026. doi:https://dx.doi.org/10.1007/s00256-021-03846-0

22. Luoma K, Vehmas T, Raininko R, Luukkonen R, Riihimaki H. Lumbosacral Transitional Vertebra: Relation to Disc Degeneration and Low Back Pain. Research Support, Non-U.S. Gov't. *Spine*. Jan 15 2004;29(2):200-5.

23. Oktay AB, Albayrak NB, Akgul YS. Computer Aided Diagnosis of Degenerative Intervertebral Disc Diseases from Lumbar Mr Images. *Computerized Medical Imaging & Graphics*. Oct 2014;38(7):613-9. doi:https://dx.doi.org/10.1016/j.compmedimag.2014.04.006
