# Supplementary material for: MRI‐Based Grading Systems for Assessing Lumbar Disc Degeneration: A Scoping Review
Source: JOR Spine. 2025 Sep 15;8(3):e70113. doi: 10.1002/jsp2.70113 (PMC12435304; doi:10.1002/jsp2.70113)
Supplement: Supplementary file 5 — Data S5: Supporting Information. [file JSP2-8-e70113-s004.docx]

**Online Resource 5.** The proportion of all grading systems reported to be used to assess disc degeneration with different methods of synthesis, stratified by the grading system components used to assess for disc degeneration

|  |  | **DDD grading performed by** | | | | | | | | |  | | | **L-spine levels reported** | | | | | | | **Methods of synthesis I** | | | | | | | |  | | | **Methods of synthesis II** | | | |
| --- | --- | --- | --- | --- | --- | --- | --- | --- | --- | --- | --- | --- | --- | --- | --- | --- | --- | --- | --- | --- | --- | --- | --- | --- | --- | --- | --- | --- | --- | --- | --- | --- | --- | --- | --- |
| Grading system components | Proportion of reported use of grading systems % (n/N) | | Radiologist % (n/N) | | Surgeon % (n/N) | | Not specified % (n/N) | | Lumbar spine reported (T12-S1)* % (n/N) | | | Single level reported % (n/N) | | | Other* % (n/N) | | Each level individually % (n/N) | | Worst level % (n/N) | | | Sum of all levels % (n/N) | | Average across all levels % (n/N) | | Not specified % (n/N) | | Continuous % (n/N) | | | Ordinal % (n/N) | | Collected as ordinal but analysed as dichotomous % (n/N) | Collected and analysed as dichotomous % (n/N) | |
| **Subjective grading systems** | **83.2**  **(556/668)** | | | **42.4 (236/556)** | | **24.1 (134/556)** | | **31.8 (177/556)** | | **53.2 (296/556)** | | | **12.6 (70/556)** | | | **34.4 (191/556)** | | **58.1 (323/556)** | | **6.1 (34/556)** | | | **14.4 (80/556)** | | **3.8 (21/556)** | | **21.8 (121/556)** | | | **9.4 (52/556)** | **58.5 (325/556)** | | **27.5 (153/556)** | | **4.7 (26/556)** |
| **DSI** | **3.8 (21/556)** | | | **57.1 (12/21)** | | **19.0 (4/21)** | | **38.1 (8/21)** | | **33.3 (7/21)** | | | **19.0 (4/21)** | | | **47.7 (10/21)** | | **71.4 (15/21)** | | **4.8 (1/21)** | | | **0.0 (0/21)** | | **0.0 (0/21)** | | **23.8 (5/21)** | | | **0.0 (0/21)** | **42.9 (9/21)** | | **14.3 (3/21)** | | **42.9 (9/21)** |
| Gibson | 19.0 (4/21) | | | 0.0 (0/4) | | 25.0 (1/4) | | 75.0 (3/4) | | 25.0 (1/4) | | | 50.0 (2/4) | | | 25.0 (1/4) | | 75.0 (3/4) | | 0.0 (0/4) | | | 0.0 (0/4) | | 0.0 (0/4) | | 25.0 (1/4) | | | 0.0 (0/4) | 100.0 (4/4) | | 0.0 (0/4) | | 0.0 (0/4) |
| Decandido | 14.3 (3/21) | | | 33.3 (1/3) | | 33.3 (1/3) | | 66.7 (2/3) | | 66.7 (2/3) | | | 33.3 (1/3) | | | 0.0 (0/3) | | 66.7 (2/3) | | 0.0 (0/3) | | | 0.0 (0/3) | | 0.0 (0/3) | | 33.3 (1/3) | | | 0.0 (0/3) | 66.7 (2/3) | | 0.0 (0/3) | | 33.3 (1/3) |
| Luoma | 9.5 (2/21) | | | 100.0 (2/2) | | 0.0 (0/2) | | 0.0 (0/2) | | 0.0 (0/2) | | | 0.0 (0/2) | | | 100.0 (2/2) | | 100.0 (2/2) | | 0.0 (0/2) | | | 0.0 (0/2) | | 0.0 (0/2) | | 0.0 (0/2) | | | 0.0 (0/2) | 0.0 (0/2) | | 50.0 (1/2) | | 50.0 (1/2) |
| Other*** | 57.1 (12/21) | | | 75.0 (9/12) | | 16.7 (2/12) | | 25.0 (3/12) | | 33.3 (4/12) | | | 8.3 (1/12) | | | 58.3 (7/12) | | 66.7 (8/12) | | 8.3 (1/12) | | | 0.0 (0/12) | | 0.0 (0/12) | | 25.0 (3/12) | | | 0.0 (0/12) | 25.0 (3/12) | | 16.7 (2/12) | | 58.3 (7/12) |
| **DH** | **0.7 (4/556)** | | | **75.0 (3/4)** | | **75.0 (3/4)** | | **0.0 (0/4)** | | **75.0 (3/4)** | | | **25.0 (1/4)** | | | **0.0 (0/4)** | | **50.0 (2/4)** | | **25.0 (1/4)** | | | **0.0 (0/4)** | | **25.0 (1/4)** | | **0.0 (0/4)** | | | **25.0 (1/4)** | **75.0 (3/4)** | | **25.0 (1/4)** | | **0.0 (0/4)** |
| **DSI and DH** | **7.9 (44/556)** | | | **43.2 (19/44)** | | **18.2 (8/44)** | | **31.8 (14/44)** | | **75.0 (33/44)** | | | **4.5 (2/44)** | | | **22.7 (10/44)** | | **45.5 (20/44)** | | **4.5 (2/44)** | | | **43.2 (19/44)** | | **2.3 (1/44)** | | **11.4 (5/44)** | | | **25.0 (11/44)** | **43.2 (19/44)** | | **25.0 (11/44)** | | **6.8 (3/44)** |
| Schneidermann | 68.2 (30/44) | | | 23.3 (7/30) | | 26.7 (8/30) | | 40 (12/30) | | 76.7 (23/30) | | | 3.3 (1/30) | | | 20.0 (6/30) | | 33.3 (10/30) | | 3.3 (1/30) | | | 60.0 (18/30) | | 3.3 (1/30) | | 6.7 (2/30) | | | 33.3 (10/30) | 43.3 (13/30) | | 23.3 (7/30) | | 0.0 (0/30) |
| Jensen | 11.4 (5/44) | | | 100.0 (5/5) | | 0.0 (0/5) | | 0.0 (0/5) | | 100.0 (5/5) | | | 0.0 (0/5) | | | 0.0 (0/5) | | 60.0 (3/5) | | 20.0 (1/5) | | | 0.0 (0/5) | | 0.0 (0/5) | | 20.0 (1/5) | | | 20.0 (1/5) | 40.0 (2/5) | | 40.0 (2/5) | | 0.0 (0/5) |
| Luoma | 4.5 (2/44) | | | 100.0 (2/2) | | 0.0 (0/2) | | 0.0 (0/2) | | 50.0 (1/2) | | | 0.0 (0/2) | | | 50.0 (1/2) | | 50.0 (1/2) | | 0.0 (0/2) | | | 0.0 (0/2) | | 0.0 (0/2) | | 50.0 (1/2) | | | 0.0 (0/2) | 100.0 (2/2) | | 0.0 (0/2) | | 0.0 (0/2) |
| Other*** | 15.9 (7/44) | | | 71.4 (5/7) | | 0.0 (0/7) | | 28.6 (2/7) | | 57.1 (4/7) | | | 14.3 (1/7) | | | 42.9 (3/7) | | 85.7 (6/7) | | 0.0 (0/7) | | | 14.3 (1/7) | | 0.0 (0/7) | | 14.3 (1/7) | | | 0.0 (0/7) | 28.6 (2/7) | | 28.6 (2/7) | | 42.9 (3/7) |
| **DSI and/or DH and/or disc bulging and herniation** | **4.1 (23/556)** | | | **60.9 (14/23)** | | **30.4 (7/23)** | | **13.0 (3/23)** | | **43.5 (10/23)** | | | **0.0 (0/23)** | | | **56.5 (13/23)** | | **34.8 (8/23)** | | **8.7 (2/23)** | | | **34.8 (8/23)** | | **8.7 (2/23)** | | **21.7 (5/23)** | | | **17.4 (4/23)** | **60.9 (14/23)** | | **0.0 (0/23)** | | **21.7 (5/23)** |
| Fardon | 17.4 (4/23) | | | 50.0 (2/4) | | 25.0 (1/2) | | 0.0 (0/2) | | 0.0 (0/2) | | | 0.0 (0/2) | | | 100.0 (4/4) | | 50.0 (2/4) | | 0.0 (0/2) | | | 0.0 (0/2) | | 0.0 (0/2) | | 50.0 (2/4) | | | 0.0 (0/2) | 25.0 (1/2) | | 0.0 (0/2) | | 75.0 (3/4) |
| Solovieva | 17.4 (4/23) | | | 100.0 (4/4) | | 0.0 (0/4) | | 0.0 (0/4) | | 0.0 (0/4) | | | 0.0 (0/4) | | | 100.0 (4/4) | | 25.0 (1/4) | | 0.0 (0/4) | | | 100.0 (4/4) | | 0.0 (0/4) | | 0.0 (0/4) | | | 0.0 (0/4) | 100.0 (4/4) | | 0.0 (0/4) | | 0.0 (0/4) |
| Witwit | 13.0 (3/23) | | | 100.0 (3/3) | | 0.0 (0/3) | | 0.0 (0/3) | | 100.0 (3/3) | | | 0.0 (0/3) | | | 0.0 (0/3) | | 66.7 (2/3) | | 0.0 (0/3) | | | 0.0 (0/3) | | 0.0 (0/3) | | 33.3 (1/3) | | | 0.0 (0/3) | 66.7 (2/3) | | 0.0 (0/3) | | 33.3 (1/3) |
| Battie | 8.7 (2/23) | | | 50.0 (1/2) | | 50.0 (1/2) | | 50.0 (1/2) | | 50.0 (1/2) | | | 0.0 (0/2) | | | 50.0 (1/2) | | 50.0 (1/2) | | 0.0 (0/2) | | | 50.0 (1/2) | | 0.0 (0/2) | | 0.0 (0/2) | | | 50.0 (1/2) | 50.0 (1/2) | | 0.0 (0/2) | | 0.0 (0/2) |
| Horton and Daftari | 8.7 (2/23) | | | 50.0 (1/2) | | 0.0 (0/2) | | 50.0 (1/2) | | 0.0 (0/2) | | | 0.0 (0/2) | | | 100.0 (2/2) | | 50.0 (1/2) | | 0.0 (0/2) | | | 50.0 (1/2) | | 0.0 (0/2) | | 0.0 (0/2) | | | 50.0 (1/2) | 50.0 (1/2) | | 0.0 (0/2) | | 0.0 (0/2) |
| Kanamori | 8.7 (2/23) | | | 0.0 (0/2) | | 100.0 (2/2) | | 0.0 (0/2) | | 100.0 (2/2) | | | 0.0 (0/2) | | | 0.0 (0/2) | | 0.0 (0/2) | | 100.0 (2/2) | | | 0.0 (0/2) | | 0.0 (0/2) | | 0.0 (0/2) | | | 0.0 (0/2) | 100.0 (2/2) | | 0.0 (0/2) | | 0.0 (0/2) |
| Videman | 8.7 (2/23) | | | 0.0 (0/2) | | 50.0 (1/2) | | 50.0 (1/2) | | 50.0 (1/2) | | | 0.0 (0/2) | | | 50.0 (1/2) | | 0.0 (0/2) | | 0.0 (0/2) | | | 0.0 (0/2) | | 50.0 (1/2) | | 50.0 (1/2) | | | 0.0 (0/2) | 100.0 (2/2) | | 0.0 (0/2) | | 0.0 (0/2) |
| Other*** | 17.4 (4/23) | | | 75.0 (3/4) | | 50.0 (2/4) | | 0.0 (0/4) | | 75.0 (3/4) | | | 0.0 (0/4) | | | 25.0 (1/4) | | 25.0 (1/4) | | 0.0 (0/4) | | | 50.0 (2/4) | | 25.0 (1/4) | | 25.0 (1/4) | | | 50.0 (2/4) | 25.0 (1/4) | | 0.0 (0/4) | | 25.0 (1/4) |
| **DSI and/or DH and/or herniation, and/or structural changes, and/or distinction between AF and NP** | **77.5 (431/556)** | | | **40.4 (174/431)** | | **23.9 (103/431)** | | **32.9 (142/431)** | | **52.4 (226/431)** | | | **11.1 (60/431)** | | | **33.6 (145/431)** | | **61.3 (264/431)** | | **6.5 (28/431)** | | | **9.0 (39/431)** | | **3.7 (16/431)** | | **23.0 (99/431)** | | | **5.6 (24/431)** | **61.3 (264/431)** | | **31.3 (135/431)** | | **1.9 (8/431)** |
| Pfirrmann | 85.8 (370/431) | | | 40.3 (149/370) | | 23.5 (87/370) | | 32.2 (119/370) | | 54.1 (200/370) | | | 13.0 (48/370) | | | 33.0 (122/370) | | 61.6 (228/370) | | 6.2 (23/370) | | | 9.7 (36/370) | | 4.3 (16/370) | | 22.2 (82/370) | | | 5.9 (22/370) | 59.5 (220/37) | | 30.3 (122/370) | | 1.6 (6/370) |
| Modified Pfirrmann | 9.7 (42/431) | | | 50.0 (21/42) | | 33.3 (14/42) | | 28.6 (12/42) | | 40.5 (17/42) | | | 26.2 (11/42) | | | 33.3 (14/42) | | 54.8 (23/42) | | 9.5 (4/42) | | | 2.4 (1/42) | | 0.0 (0/42) | | 33.3 (14/42) | | | 4.8 (2/42) | 76.2 (32/42) | | 16.7 (7/42) | | 2.4 (1/42) |
| Thompson | 2.1 (9/431) | | | 0.0 (0/9) | | 22.2 (2/9) | | 66.7 (6/9) | | 44.4 (4/9) | | | 0.0 (0/9) | | | 55.6 (5/9) | | 66.7 (6/9) | | 0.0 (0/9) | | | 11.1 (1/9) | | 0.0 (0/9) | | 22.2 (2/9) | | | 0.0 (0/9) | 77.8 (7/9) | | 22.2 (2/9) | | 0.0 (0/9) |
| Buirski | 0.7 (3/431) | | | 0.0 (0/3) | | 0.0 (0/3) | | 66.7 (2/3) | | 33.3 (1/3) | | | 0.0 (0/3) | | | 66.7 (2/3) | | 66.7 (2/3) | | 0.0 (0/3) | | | 0.0 (0/3) | | 0.0 (0/3) | | 33.3 (1/3) | | | 0.0 (0/3) | 33.3 (1/3) | | 66.7 (2/3) | | 0.0 (0/3) |
| Modified Pearce | 0.5 (2/431 | | | 100.0 (2/2) | | 0.0 (0/2) | | 0.0 (0/2) | | 0.0 (0/2) | | | 0.0 (0/2) | | | 100.0 (2/2) | | 100.0 (2/2) | | 0.0 (0/2) | | | 0.0 (0/2) | | 0.0 (0/2) | | 0.0 (0/2) | | | 0.0 (0/2) | 100.0 (2/2) | | 0.0 (0/2) | | 0.0 (0/2) |
| Woodend Classification | 0.5 (2/431 | | | 0.0 (0/2) | | 0.0 (0/2) | | 100.0 (2/2) | | 50.0 (1/2) | | | 50.0 (1/2) | | | 0.0 (0/2) | | 50.0 (1/2) | | 50.0 (1/2) | | | 0.0 (0/2) | | 0.0 (0/2) | | 0.0 (0/2) | | | 0.0 (0/2) | 100.0 (2/2) | | 0.0 (0/2) | | 0.0 (0/2) |
| Other*** | 0.7 (3/431) | | | 66.7 (2/3) | | 0.0 (0/3) | | 33.3 (1/3) | | 100 (3/3) | | | 0.0 (0/3) | | | 0.0 (0/3) | | 66.7 (2/3) | | 0.0 (0/3) | | | 33.3 (1/3) | | 0.0 (0/3) | | 0.0 (0/3) | | | 0.0 (0/3) | 0.0 (0/3) | | 66.7 (2/3) | | 33.3 (1/3) |
| **DSI and/or DH and/or osteophytes, end-plate changes, Modic changes and high intensity zones (HIZ)** | **5.9 (33/556)** | | | **42.4 (14/33)** | | **27.3 (9/33)** | | **30.3 (10/33)** | | **51.5 (17/33)** | | | **9.1 (3/33)** | | | **39.4 (13/33)** | | **42.4 (14/33)** | | **0.0 (0/33)** | | | **42.4 (14/33)** | | **3.0 (1/33)** | | **21.2 (7/33)** | | | **39.4 (13/33)** | **48.5 (16/33)** | | **9.1 (3/33)** | | **3.0 (1/33)** |
| Jarosz Atlas | 36.4 (12/33) | | | 16.7 (2/12) | | 0.0 (0/12) | | 58.3 (7/12) | | 58.3 (7/12) | | | 0.0 (0/12) | | | 41.7 (5/12) | | 33.3 (4/12) | | 0.0 (0/12) | | | 91.7 (11/12) | | 0.0 (0/12) | | 0.0 (0/12) | | | 75.0 (9/12) | 16.7 (2/12) | | 8.3 (1/12) | | 0.0 (0/12) |
| Pearce | 18.2 (6/33) | | | 66.7 (4/6) | | 16.7 (1/6) | | 16.7 (1/6) | | 16.7 (1/6) | | | 16.7 (1/6) | | | 66.7 (4/6) | | 33.3 (2/6) | | 0.0 (0/6) | | | 0.0 (0/6) | | 0.0 (0/6) | | 66.7 (4/6) | | | 0.0 (0/6) | 66.7 (4/6) | | 33.3 (2/6) | | 0.0 (0/6) |
| Battie | 6.1 (2/33) | | | 50.0 (1/2) | | 100.0 (2/2) | | 0.0 (0/2) | | 100.0 (2/2) | | | 0.0 (0/2) | | | 0.0 (0/2) | | 50.0 (1/2) | | 0.0 (0/2) | | | 0.0 (0/2) | | 0.0 (0/2) | | 50.0 (1/2) | | | 0.0 (0/2) | 100.0 (2/2) | | 0.0 (0/2) | | 0.0 (0/2) |
| Benneker | 6.1 (2/33) | | | 100.0 (2/2) | | 0.0 (0/2) | | 0.0 (0/2) | | 100.0 (2/2) | | | 0.0 (0/2) | | | 0.0 (0/2) | | 0.0 (0/2) | | 0.0 (0/2) | | | 100.0 (2/2) | | 0.0 (0/2) | | 0.0 (0/2) | | | 100.0 (2/2) | 0.0 (0/2) | | 0.0 (0/2) | | 0.0 (0/2) |
| Tuft degenerative disc classification | 6.1 (2/33) | | | 50.0 (1/2) | | 100.0 (2/2) | | 0.0 (0/2) | | 100.0 (2/2) | | | 0.0 (0/2) | | | 0.0 (0/2) | | 100.0 (2/2) | | 0.0 (0/2) | | | 0.0 (0/2) | | 0.0 (0/2) | | 0.0 (0/2) | | | 50.0 (1/2) | 50.0 (1/2) | | 0.0 (0/2) | | 0.0 (0/2) |
| Other | 27.3 (9/33) | | | 44.4 (4/9) | | 44.4 (4/9) | | 22.2 (2/9) | | 33.3 (3/9) | | | 22.2 (2/9) | | | 44.4 (4/9) | | 55.6 (5/9) | | 0.0 (0/9) | | | 11.1 (1/9) | | 11.1 (1/9) | | 22.2 (2/9) | | | 11.1 (1/9) | 77.8 (7/9) | | 0.0 (0/9) | | 11.1 (1/9) |
| **Quantitative grading systems** | **16.8 (112/668)** | | | **33.0 (37/112)** | | **11.6 (13/112)** | | **54.5 (61/112)** | | **60.7 (68/112)** | | | **3.6 (4/112)** | | | **35.7 (40/112)** | | **76.8 (86/112)** | | **2.7 (3/112)** | | | **3.6 (4/112)** | | **6.3 (7/112)** | | **13.4 (15/112)** | | | **92.0 (103/112)** | **6.3 (7/112)** | | **0.0 (0/112)** | | **3.6 (4/112)** |
| **DSI** | **17.9 (20/112)** | | | **25.0 (5/20)** | | **20.0 (4/20)** | | **65.0 (13/20)** | | **50.0 (10/20)** | | | **5.0 (1/20)** | | | **45.0 (9/20)** | | **65.0 (13/20)** | | **5.0 (1/20)** | | | **0.0 (0/20)** | | **15.0 (3/20)** | | **25.0 (5/20)** | | | **75.0 (15/20)** | **15.0 (3/20)** | | **0.0 (0/20)** | | **10.0 (2/20)** |
| Videman | 25.0 (5/20) | | | 0.0 (0/5) | | 0.0 (0/5) | | 100.0 (5/5) | | 80.0 (4/5) | | | 0.0 (0/5) | | | 20.0 (1/5) | | 40.0 (2/5) | | 0.0 (0/5) | | | 0.0 (0/5) | | 40.0 (2/5) | | 20.0 (1/5) | | | 100.0 (5/5) | 0.0 (0/5) | | 0.0 (0/5) | | 0.0 (0/5) |
| Paajanen | 20.0 (4/20) | | | 0.0 (0/4) | | 0.0 (0/4) | | 100.0 (4/4) | | 0.0 (0/4) | | | 0.0 (0/4) | | | 100.0 (4/4) | | 25.0 (1/4) | | 0.0 (0/4) | | | 0.0 (0/4) | | 0.0 (0/4) | | 75.0 (3/4) | | | 0.0 (0/4) | 50.0 (2/4) | | 0.0 (0/4) | | 50.0 (2/4) |
| Battie | 10.0 (2/20) | | | 0.0 (0/2) | | 50.0 (1/2) | | 50.0 (1/2) | | 100.0 (2/2) | | | 0.0 (0/2) | | | 0.0 (0/2) | | 100.0 (2/2) | | 50.0 (1/2) | | | 0.0 (0/2) | | 0.0 (0/2) | | 50.0 (1/2) | | | 100.0 (2/2) | 0.0 (0/2) | | 0.0 (0/2) | | 0.0 (0/2) |
| Luoma | 10.0 (2/20) | | | 100.0 (2/2) | | 0.0 (0/2) | | 0.0 (0/2) | | 0.0 (0/2) | | | 50.0 (1/2) | | | 50.0 (1/2) | | 100.0 (2/2) | | 0.0 (0/2) | | | 0.0 (0/2) | | 0.0 (0/2) | | 0.0 (0/2) | | | 100.0 (2/2) | 0.0 (0/2) | | 0.0 (0/2) | | 0.0 (0/2) |
| Nagashima | 10.0 (2/20) | | | 0.0 (0/2) | | 0.0 (0/2) | | 100.0 (2/2) | | 100.0 (2/2) | | | 0.0 (0/2) | | | 0.0 (0/2) | | 50.0 (1/2) | | 0.0 (0/2) | | | 0.0 (0/2) | | 50.0 (1/2) | | 0.0 (0/2) | | | 100.0 (2/2) | 0.0 (0/2) | | 0.0 (0/2) | | 0.0 (0/2) |
| Other*** | 25.0 (5/20) | | | 60.0 (3/5) | | 60.0 (3/5) | | 20.0 (1/5) | | 40.0 (2/5) | | | 0.0 (0/5) | | | 60.0 (3/5) | | 100.0 (5/5) | | 0.0 (0/5) | | | 0.0 (0/5) | | 0.0 (0/5) | | 0.0 (0/5) | | | 80.0 (4/5) | 20.0 (1/5) | | 0.0 (0/5) | | 0.0 (0/5) |
| **DH** | **2.7 (3/112)** | | | **0.0 (0/3)** | | **0.0 (0/3)** | | **100 (3/3)** | | **66.7 (2/3)** | | | **0.0 (0/3)** | | | **33.3 (1/3)** | | **100 (3/3)** | | **0.0 (0/3)** | | | **0.0 (0/3)** | | **33.3 (1/3)** | | **0.0 (0/3)** | | | **100 (3/3)** | **0.0 (0/3)** | | **0.0 (0/3)** | | **0.0 (0/3)** |
| **Disc bulging** | **3.6 (4/112)** | | | **75.0 (3/4)** | | **0.0 (0/4)** | | **25.0 (1/4)** | | **25.0 (1/4)** | | | **0.0 (0/4)** | | | **75.0 (3/4)** | | **50.0 (2/4)** | | **0.0 (0/4)** | | | **0.0 (0/4)** | | **25.0 (1/4)** | | **25.0 (1/4)** | | | **50.0 (2/4)** | **0.0 (0/4)** | | **0.0 (0/4)** | | **50.0 (2/4)** |
| Luoma | 75.0 (3/4) | | | 100 (3/3) | | 0.0 (0/3) | | 0.0 (0/3) | | 0.0 (0/3) | | | 0.0 (0/3) | | | 100 (3/3) | | 66.7 (2/3) | | 0.0 (0/3) | | | 0.0 (0/3) | | 0.0 (0/3) | | 33.3 (1/3) | | | 0.0 (0/3) | 33.3 (1/3) | | 0.0 (0/3) | | 66.7 (2/3) |
| Other*** | 25.0 (1/4) | | | 0.0 (0/1) | | 0.0 (0/1) | | 100.0 (1/1) | | 100.0 (1/1) | | | 0.0 (0/1) | | | 0.0 (0/1) | | 0.0 (0/1) | | 0.0 (0/1) | | | 0.0 (0/1) | | 100.0 (1/1) | | 0.0 (0/1) | | | 100.0 (1/1) | 0.0 (0/1) | | 0.0 (0/1) | | 0.0 (0/1) |
| **DSI and DH** | **2.7 (3/112)** | | | **66.7 (2/3)** | | **33.3 (1/3)** | | **0.0 (0/3)** | | **66.7 (2/3)** | | | **0.0 (0/3)** | | | **33.3 (1/3)** | | **66.7 (2/3)** | | **0.0 (0/3)** | | | **33.3 (1/3)** | | **0.0 (0/3)** | | **0.0 (0/3)** | | | **100 (3/3)** | **0.0 (0/3)** | | **0.0 (0/3)** | | **0.0 (0/3)** |
| **DSI, DH, and disc bulging** | **8.9 (10/112)** | | | **20.0 (2/10)** | | **20.0 (2/10)** | | **30.0 (3/10)** | | **80.0 (8/10)** | | | **10.0 (1/10)** | | | **10.0 (1/10)** | | **70.0 (7/10)** | | **0.0 (0/10)** | | | **10.0 (1/10)** | | **0.0 (0/10)** | | **20.0 (2/10)** | | | **80.0 (8/10)** | **20.0 (2/10)** | | **0.0 (0/10)** | | **0.0 (0/10)** |
| Battie | 30.0 (3/10) | | | 0.0 (0/3) | | 0.0 (0/3) | | 66.7 (2/3) | | 100 (3/3) | | | 0.0 (0/3) | | | 0.0 (0/3) | | 33.3 (1/3) | | 0.0 (0/3) | | | 33.3 (1/3) | | 0.0 (0/3) | | 33.3 (1/3) | | | 66.7 (2/3) | 33.3 (1/3) | | 0.0 (0/3) | | 0.0 (0/3) |
| Feng | 30.0 (3/10) | | | 0.0 (0/3) | | 0.0 (0/3) | | 33.3 (1/3) | | 66.7 (2/3) | | | 0.0 (0/3) | | | 33.3 (1/3) | | 66.7 (2/3) | | 0.0 (0/3) | | | 0.0 (0/3) | | 0.0 (0/3) | | 33.3 (1/3) | | | 100 (3/3) | 0.0 (0/3) | | 0.0 (0/3) | | 0.0 (0/3) |
| Other | 40.0 (4/10) | | | 50.0 (2/4) | | 50.0 (2/4) | | 0.0 (0/4) | | 75.0 (3/4) | | | 25.0 (1/4) | | | 0.0 (0/4) | | 100.0 (4/4) | | 0.0 (0/4) | | | 0.0 (0/4) | | 0.0 (0/4) | | 0.0 (0/4) | | | 75.0 (3/4) | 25.0 (1/4) | | 0.0 (0/4) | | 0.0 (0/4) |
| **Specialized quantitative MRI techniques and sequences** | **64.3 (72/112)** | | | **34.8 (25/72)** | | **8.3 (6/72)** | | **56.9 (41/72)** | | **62.5 (45/72)** | | | **2.8 (2/72)** | | | **34.7 (25/72)** | | **81.9 (59/72)** | | **2.8 (2/72)** | | | **2.8 (2/72)** | | **2.8 (2/72)** | | **9.7 (7/72)** | | | **97.2 (70/76)** | **2.8 (2/72)** | | **0.0 (0/72)** | | **0.0 (0/72)** |
| **Summary of subjective and quantitative grading systems** | **668** | | | **40.9 (273/668)** | | **22.0 (147/668)** | | **35.6 (238/668)** | | **54.5 (364/668)** | | | **11.1 (74/668)** | | | **34.6 (231/668)** | | **61.2 (409/668)** | | **5.5 (37/668)** | | | **12.6 (84/668)** | | **4.2 (28/668)** | | **20.4 (136/668)** | | | **23.2 (155/668)** | **49.7 (332/668)** | | **22.9 (153/668)** | | **4.5 (30/668)** |

DSI: disc signal intensity, DH: disc height, AF: annulus fibrosis, NP: nucleus pulposus, MRI: magnetic resonance imaging

*Included combinations of T12-L5, T12-S1, L1-L5, and L5-S1. ‘Other’ category includes unspecified, and all other combinations reported

** The total number of responses may exceed the number of reports of grading system use due to the possibility of multiple options

*** Grading systems listed into the ‘Other’ category were used in <2 studies
