# Supplementary material for: MRI‐Based Grading Systems for Assessing Lumbar Disc Degeneration: A Scoping Review
Source: JOR Spine. 2025 Sep 15;8(3):e70113. doi: 10.1002/jsp2.70113 (PMC12435304; doi:10.1002/jsp2.70113)
Supplement: Supplementary file 6 — Data S6: Supporting Information. [file JSP2-8-e70113-s006.docx]

**Online Resource 6.** The proportion of grading systems reported to be assessed for measurement properties, stratified by the grading system components used to assess for disc degeneration

|  |  | **Reliability** | | **Sensitivity to change** |  | **Validity** |  |
| --- | --- | --- | --- | --- | --- | --- | --- |
| Grading system components | Proportion of reported use of grading systems % (n/N) | Intra-rater reliability % (n/N) | Inter-rater reliability % (n/N) | Use of a change score % (n/N) | Comparative evaluation with another grading system % (n/N) | Measured associations between DDD and other variables % (n/N) | Measured associations between DDD and LBP % (n/N) |
| Subjective grading system | **83.2 (556/668)** | **28.1 (156/556)** | **34.5 (192/556)** | **11.0 (61/556)** | **14.6 (81/556)** | **46.2 (257/556)** | **18.0 (100/556)** |
| **DSI** | **3.8 (21/556)** | **23.8 (5/21)** | **33.3 (7/21)** | **0.0 (0/21)** | **9.5 (2/21)** | **47.6 (10/21)** | **28.6 (6/21)** |
| Gibson | 19.0 (4/21) | 25.0 (1/4) | 25.0 (1/4) | 0.0 (0/4) | 0.0 (0/4) | 25.0 (1/4) | 25.0 (1/4) |
| Decandido | 14.3 (3/21) | 0.0 (0/3) | 0.0 (0/3) | 0.0 (0/3) | 0.0 (0/3) | 66.7 (2/3) | 0.0 (0/3) |
| Luoma | 9.5 (2/21) | 100.0 (2/2) | 50.0 (1/2) | 0.0 (0/2) | 0.0 (0/2) | 100.0 (2/2) | 50.0 (1/2) |
| Other* | 57.1 (12/21) | 16.7 (2/12) | 41.7 (5/12) | 0.0 (0/12) | 16.7 (2/12) | 41.7 (5/12) | 33.3 (4/12) |
| **DH** | **0.7 (4/556)** | **25.0 (1/4)** | **50.0 (2/4)** | **0.0 (0/4)** | **0.0 (0/4)** | **75.0 (3/4)** | **50.0 (2/4)** |
| **DSI and DH** | **7.9 (44/556)** | **31.8 (14/44)** | **45.5 (20/44)** | **11.4 (5/44)** | **11.4 (5/44)** | **54.5 (24/44)** | **31.8 (14/44)** |
| Schneidermann | 68.2 (30/44) | 20.0 (6/30) | 40.0 (12/30) | 10.0 (3/30) | 16.7 (5/30) | 53.3 (16/30) | 33.3 (10/30) |
| Jensen | 11.4 (5/44) | 80.0 (4/5) | 80.0 (4/5) | 0.0 (0/5) | 0.0 (0/5) | 60.0 (3/5) | 20.0 (1/5) |
| Luoma | 4.5 (2/44) | 100.0 (2/2) | 100.0 (2/2) | 50.0 (1/2) | 0.0 (0/2) | 50.0 (1/2) | 50.0 (1/2) |
| Other* | 15.9 (7/44) | 28.6 (2/7) | 28.6 (2/7) | 14.3 (1/7) | 0.0 (0/7) | 57.1 (4/7) | 28.6 (2/7) |
| **DSI and/or DH and/or disc bulging and herniation** | **4.1 (23/556)** | 30.4 (7/23) | 39.1 (9/23) | 13.0 (3/23) | 17.4 (4/23) | 52.2 (12/23) | 34.8 (8/23) |
| Fardon | 17.4 (4/23) | 0.0 (0/4) | 25.0 (1/4) | 0.0 (0/4) | 25.0 (1/4) | 50.0 (2/4) | 25.0 (1/4) |
| Solovieva | 17.4 (4/23) | 0.0 (0/4) | 100.0 (4/4) | 0.0 (0/4) | 0.0 (0/4) | 0.0 (0/4) | 0.0 (0/4) |
| Witwit | 13.0 (3/23) | 100.0 (3/3) | 33.3 (1/3) | 100.0 (3/3) | 0.0 (0/3) | 100.0 (3/3) | 100.0 (3/3) |
| Battie | 8.7 (2/23) | 50.0 (1/2) | 100.0 (2/2) | 0.0 (0/2) | 0.0 (0/2) | 100.0 (2/2) | 0.0 (0/2) |
| Horton and Daftari | 8.7 (2/23) | 0.0 (0/2) | 0.0 (0/2) | 0.0 (0/2) | 50.0 (1/2) | 50.0 (1/2) | 50.0 (1/2) |
| Kanamori | 8.7 (2/23) | 100.0 (2/2) | 100.0 (2/2) | 0.0 (0/2) | 100.0 (2/2) | 100.0 (2/2) | 100.0 (2/2) |
| Videman | 8.7 (2/23) | 50.0 (1/2) | 0.0 (0/2) | 0.0 (0/2) | 0.0 (0/2) | 0.0 (0/2) | 0.0 (0/2) |
| Other* | 17.4 (4/23) | 0.0 (0/4) | 0.0 (0/4) | 0.0 (0/4) | 0.0 (0/4) | 50.0 (2/4) | 25.0 (1/4) |
| **DSI and/or DH and/or herniation, structural changes, and distinction between AF and NP** | **77.5 (431/556)** | **26.7 (115/431)** | **33.4 (144/431)** | **10.9 (47/431)** | **16.0 (69/431)** | **43.6 (188/431)** | **13.9 (60/431)** |
| Pfirrmann | 85.8 (370/431) | 27.3 (101/370) | 34.6 (128/370) | 11.1 (41/370) | 15.1 (56/370) | 44.3 (164/370) | 14.3 (53/370) |
| Modified Pfirrmann | 9.7 (42/431) | 21.4 (9/42) | 26.2 (11/42) | 14.3 (6/42) | 21.4 (9/42) | 33.3 (14/42) | 0.0 (0/42) |
| Thompson | 2.1 (9/431) | 33.3 (3/9) | 33.3 (3/9) | 0.0 (0/9) | 11.1 (1/9) | 11.1 (1/9) | 11.1 (1/9) |
| Buirski | 0.7 (3/431) | 0.0 (0/3) | 0.0 (0/3) | 0.0 (0/3) | 0.0 (0/3) | 100.0 (3/3) | 33.3 (1/3) |
| Modified Pearce | 0.5 (2/431) | 0.0 (0/2) | 0.0 (0/2) | 0.0 (0/2) | 50.0 (1/2) | 100.0 (2/2) | 100.0 (2/2) |
| Woodend Classification | 0.5 (2/431) | 50.0 (1/2) | 50.0 (1/2) | 0.0 (0/2) | 50.0 (1/2) | 100.0 (2/2) | 100.0 (2/2) |
| Other* | 0.7 (3/431) | 33.3 (1/3) | 33.3 (1/3) | 0.0 (0/3) | 33.3 (1/3) | 66.7 (2/3) | 33.3 (1/3) |
| **DSI and/or DH and/or osteophytes, end-plate changes, Modic changes and high intensity zones** | **5.9 (33/556)** | **42.4 (14/33)** | **30.3 (10/33)** | **18.2 (6/33)** | **3.0 (1/33)** | **60.6 (20/33)** | **30.3 (10/33)** |
| Jarosz Atlas | 36.4 (12/33) | 33.3 (4/12) | 16.7 (2/12) | 25.0 (3/12) | 0.0 (0/12) | 75.0 (9/12) | 33.3 (4/12) |
| Pearce | 18.2 (6/33) | 16.7 (1/6) | 16.7 (1/6) | 33.3 (2/6) | 0.0 (0/6) | 66.7 (4/6) | 33.3 (2/6) |
| Battie | 6.1 (2/33) | 50.0 (1/2) | 0.0 (0/2) | 0.0 (0/2) | 0.0 (0/2) | 100.0 (2/2) | 50.0 (1/2) |
| Benneker | 6.1 (2/33) | 50.0 (1/2) | 50.0 (1/2) | 0.0 (0/2) | 0.0 (0/2) | 100.0 (2/2) | 100.0 (2/2) |
| Tuft degenerative disc classification | 6.1 (2/33) | 100.0 (2/2) | 100.0 (2/2) | 0.0 (0/2) | 0.0 (0/2) | 0.0 (0/2) | 0.0 (0/2) |
| Other* | 27.3 (9/33) | 55.6 (5/9) | 44.4 (4/9) | 11.1 (1/9) | 11.1 (1/9) | 33.3 (3/9) | 11.1 (1/9) |
| Quantitative grading systems | **16.8 (112/668)** | **42.9 (48/112)** | **35.7 (40/112)** | **9.8 (11/112)** | **61.6 (69/112)** | **33.9 (38/112)** | **10.7 (12/112)** |
| **DSI** | **17.9 (20/112)** | **30.0 (6/20)** | **20.0 (4/20)** | **25.0 (5/20)** | **15.0 (3/20)** | **55.0 (11/20)** | **25.0 (5/20)** |
| Videman | 25.0 (5/20) | 20.0 (1/5) | 40.0 (2/5) | 0.0 (0/5) | 0.0 (0/5) | 80.0 (4/5) | 0.0 (0/5) |
| Paajanen | 20.0 (4/20) | 25.0 (1/4) | 0.0 (0/4) | 50.0 (2/4) | 0.0 (0/4) | 50.0 (2/4) | 50.0 (2/4) |
| Battie | 10.0 (2/20) | 0.0 (0/2) | 0.0 (0/2) | 0.0 (0/2) | 0.0 (0/2) | 100.0 (2/2) | 0.0 (0/2) |
| Luoma | 10.0 (2/20) | 50.0 (1/2) | 0.0 (0/2) | 0.0 (0/2) | 0.0 (0/2) | 0.0 (0/2) | 0.0 (0/2) |
| Nagashima | 10.0 (2/20) | 50.0 (1/2) | 50.0 (1/2) | 50.0 (1/2) | 50.0 (1/2) | 50.0 (1/2) | 50.0 (1/2) |
| Other* | 25.0 (5/20) | 40.0 (2/5) | 20.0 (1/5) | 40.0 (2/5) | 40.0 (2/5) | 40.0 (2/5) | 40.0 (2/5) |
| **DH** | **2.7 (3/112)** | **33.3 (1/3)** | **0.0 (0/3)** | **0.0 (0/3)** | **33.3 (1/3)** | **66.7 (2/3)** | **0.0 (0/3)** |
| **Disc bulging** | **3.6 (4/112)** | **75.0 (3/4)** | **50.0 (2/4)** | **0.0 (0/4)** | **0.0 (0/4)** | **100.0 (4/4)** | **50.0 (2/4)** |
| Luoma | 75.0 (3/4) | 100.0 (3/3) | 33.3 (1/3) | 0.0 (0/3) | 0.0 (0/3) | 100.0 (3/3) | 66.7 (2/3) |
| Other* | 25.0 (1/4) | 0.0 (0/1) | 100.0 (1/1) | 0.0 (0/1) | 0.0 (0/1) | 100.0 (1/1) | 0.0 (0/1) |
| **DSI and DH** | **2.7 (3/112)** | **0.0 (0/3)** | **33.3 (1/3)** | **33.3 (1/3)** | **33.3 (1/3)** | **66.7 (2/3)** | **33.3 (1/3)** |
| **DSI, DH, and disc bulging** | **8.9 (10/112)** | **70.0 (7/10)** | **70.0 (7/10)** | **20.0 (2/10)** | **20.0 (2/10)** | **60.0 (6/10)** | **10.0 (1/10)** |
| Battie | 30.0 (3/10) | 66.7 (2/3) | 66.7 (2/3) | 0.0 (0/3) | 33.3 (1/3) | 66.7 (2/3) | 0.0 (0/3) |
| Feng | 30.0 (3/10) | 33.3 (1/3) | 33.3 (1/3) | 33.3 (1/3) | 0.0 (0/3) | 100.0 (3/3) | 33.3 (1/3) |
| Other* | 40.0 (4/10) | 100.0 (4/4) | 100.0 (4/4) | 25.0 (1/4) | 25.0 (1/4) | 25.0 (1/4) | 0.0 (0/4) |
| **Specialized quantitative MRI techniques and sequences** | **64.3 (72/112)** | **43.1 (31/72)** | **36.1 (26/72)** | **4.2 (3/72)** | **86.1 (62/72)** | **18.1 (13/72)** | **4.2 (3/72)** |
| **Summary of subjective and quantitative grading systems** | **668** | **30.5 (204/668)** | **34.7 (232/668)** | **10.8 (72/668)** | **22.5 (150/668)** | **44.2 (295/668)** | **16.8 (112/668)** |

DSI: disc signal intensity, DH: disc height, AF: annulus fibrosis, NP: nucleus pulposus, MRI: magnetic resonance imaging, DD: disc degeneration

*Grading systems listed into the ‘Other’ category were used in <2 studies
